# Constructing conjugate vaccine against *Salmonella* Typhimurium using lipid-A free lipopolysaccharide

**DOI:** 10.1186/s12929-020-00681-8

**Published:** 2020-08-24

**Authors:** Tzu-Wei Chiu, Chi-Jiun Peng, Ming-Cheng Chen, Mei-Hua Hsu, Yi-Hua Liang, Cheng-Hsun Chiu, Jim-Min Fang, Yuan Chuan Lee

**Affiliations:** 1 Department of Chemistry, National Taiwan University, 1, Sec. 4, Roosevelt Rd, Taipei, 10617 Taiwan; 2 Molecular Infectious Disease Research Center, Chang Gung Memorial Hospital, 5, Fuxing St., Guishan Dist, Taoyuan, 33302 Taiwan; 3 Department of Pediatrics, Chang Gung Children’s Hospital, 5, Fuxing St., Guishan Dist, Taoyuan, 33302 Taiwan; 4 Graduate Institute of Biomedical Sciences, College of Medicine, Chang Gung University, 259 Wenhua 1st Road, Guishan Dist, Taoyuan, 33302 Taiwan; 5 The Genomics Research Center, Academia Sinica, 128, Sec. 2, Academia Rd, Taipei, 11529 Taiwan; 6 Department of Biology, Johns Hopkins University, 3400 North Charles St, Baltimore, MD 21218-2685 USA

**Keywords:** *Salmonella* Typhimurium, Lipopolysaccharide, Carbohydrate vaccine, Flagellin

## Abstract

**Background:**

*Salmonella enterica* serotype Typhimurium is a nontyphoidal and common foodborne pathogen that causes serious threat to humans. There is no licensed vaccine to prevent the nontyphoid bacterial infection caused by *S.* Typhimurium.

**Methods:**

To develop conjugate vaccines, the bacterial lipid-A free lipopolysaccharide (LFPS) is prepared as the immunogen and used to synthesize the LFPS–linker–protein conjugates **6a**–**9b**. The designed bifunctional linkers **1**–**5** comprising either an *o*-phenylenediamine or amine moiety are specifically attached to the exposed 3-deoxy-D-manno-octulosonic acid (Kdo), an α-ketoacid saccharide of LFPS, via condensation reaction or decarboxylative amidation. In addition to bovine serum albumin and ovalbumin, the *S.* Typhimurium flagellin (FliC) is also used as a self-adjuvanting protein carrier.

**Results:**

The synthesized conjugate vaccines are characterized by sodium dodecyl sulfate polyacrylamide gel electrophoresis (SDS-PAGE) and fast performance liquid chromatography (FPLC), and their contents of polysaccharides and protein are determined by phenol–sulfuric acid assay and bicinchoninic acid assay, respectively. Enzyme-linked immunosorbent assay (ELISA) shows that immunization of mouse with the LFPS–linker–protein vaccines at a dosage of 2.5 μg is sufficient to elicit serum immunoglobulin G (IgG) specific to *S.* Typhimurium lipopolysaccharide (LPS). The straight-chain amide linkers in conjugates **7a**–**9b** do not interfere with the desired immune response. Vaccines **7a** and **7b** derived from either unfractionated LFPS or the high-mass portion show equal efficacy in induction of IgG antibodies. The challenge experiments are performed by oral gavage of *S*. Typhimurium pathogen, and vaccine **7c** having FliC as the self-adjuvanting protein carrier exhibits a high vaccine efficacy of 74% with 80% mice survival rate at day 28 post the pathogen challenge.

**Conclusions:**

This study demonstrates that lipid-A free lipopolysaccharide prepared from Gram-negative bacteria is an appropriate immunogen, in which the exposed Kdo is connected to bifunctional linkers to form conjugate vaccines. The decarboxylative amidation of Kdo is a novel and useful method to construct a relatively robust and low immunogenic straight-chain amide linkage. The vaccine efficacy is enhanced by using bacterial flagellin as the self-adjuvanting carrier protein.

**Graphical abstract:**

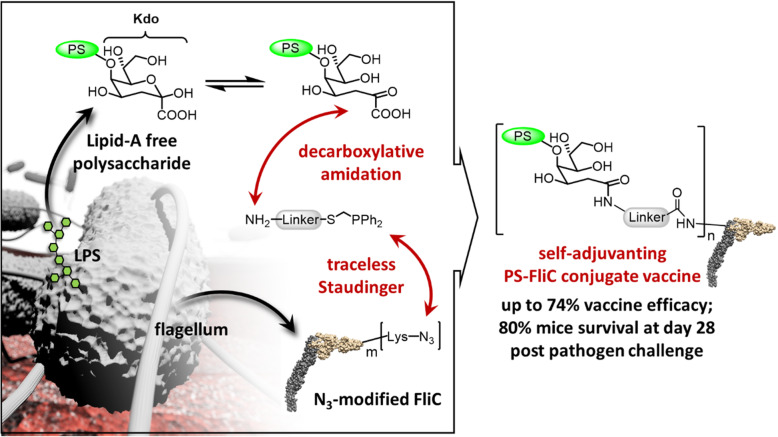

## Background

*Salmonella enterica* serotype Typhimurium (abbreviated here as *S.* Typhimurium) is a rod-shaped, flagellated Gram-negative bacterium. This nontyphoidal *Salmonella* is a common foodborne pathogen that causes numerous diarrheal infections, which are characterized by fever, gastroenteritis, abdominal cramps and excessive watery stool. *S.* Typhimurium also causes fatal invasive diseases such as meningitis, sepsis and bacteremia in countries with inadequate sanitation. The diseases are particularly severe to infants, the elderly and immunocompromised patients. The drug-resistant *S.* Typhimurium is listed as a serious threat to humans [1].

Gram-negative bacteria possess a unique lipopolysaccharide (LPS) component in the outer membrane of cell wall. LPS is the recognition target of immune cells, such as monocytes and macrophages via toll-like receptor 4 (TLR4). The innate immune response to bacterial LPS will trigger proinflammatory mediators, such as TNF-α, IL-6 and IL-1β. Thus, infection by Gram-negative bacteria can be related to the increase of cytokines [2]. The structure of LPS usually consists of O-specific polysaccharide, core polysaccharide and lipid A (Figure S1A in Supporting Information), though the composition and types of monosaccharide vary in different species. The O-specific polysaccharide in the outermost layer of LPS is also called O-antigen, because it is the major target for recognition by host antibodies. Bacterial O-specific polysaccharide usually contains more than 60 monosaccharides and 30 different non-carbohydrate components in varied sequences, chemical linkages, substitution and ring forms [2]. The O-antigen of *S.* Typhimurium contains D-Abe-(α1 → 3)-[D-Man-(α1 → 4)-L-Rha-(α1 → 3)-D-Gal-(α1 → 2)-] tetrasaccharide repeating units, wherein Abe is abequose (i.e., 3,6-dideoxy-D-galactose). O-Antigen may contain various modes of glycosylation and *O*-acetylation, which are responsible for the serological specificity of bacteria.

The O-antigen is attached to the non-reducing end of core polysaccharide, while the 3-deoxy-D-manno-octulosonic acid (Kdo) saccharide at the reducing end of the inner region links to the glucosamine (GlcN) moiety of lipid A, which typically comprises a β-D-GlcN-(1 → 6)-α-D-GlcN disaccharide with varied acyl chains and two phosphoryl substituents. Lipid A is also known as an endotoxin, which may cause shock and death by infection of gram-negative bacteria. The acyl chains in varied numbers and lengths are connected to GlcN–GlcN disaccharide by ester or amide linkage, and manifest different degrees of toxicity. Prior removal of lipid A or the acyl chains is necessary for safe handling of the bacterial surface polysaccharides. On treatment of LPS with alkali, the fatty acyl chains can be removed to give deacylated LPS. As the linkage between Kdo and GlcN is more labile to acidic hydrolysis than other glycosidic bonds in LPS [3], one can treat LPS in mild acidic condition for selective cleavage of the Kdo–GlcN linkage to obtain the lipid-A free polysaccharide (LFPS).

As antibiotics for the treatment of Gram-negative pathogen infections are getting less effective, development of powerful vaccines is an alternative approach to control and prevent *Salmonella* infectious diseases. For prevention of typhoid *Salmonella* infections, whole-cell killed vaccine, live attenuated *Salmonella* vaccine, and subunit vaccine using Vi capsular polysaccharide are available [4, 5]. In addition, the outer membrane protein (porin) [6], O-specific polysaccharide [7, 8] and deacylated LPS [9] can also be used as immunogens to produce typhoid *Salmonella* vaccines. However, no licensed vaccine is currently available to combat the nontyphoid bacterial infection caused by *S.* Typhimurium.

Though O-antigen can exhibit certain immunogenicity [10], the polysaccharide generally only acts as a hapten to activate T-cell-independent host defense mechanisms to induce a short-term, IgM-dependent immune response [11], but fails to trigger memory B cells to produce immunoglobulin G (IgG) for long-term immunity [12]. In contrast, conjugation of the polysaccharide with a proper protein carrier can elicit the immune response through T-cell-dependent pathway to induce maturation of plasma and memory B cells [10]. This event leads to long-term immunological memory and production of high-affinity antibodies to prevent bacterial infection. Conjugated carbohydrate vaccines have proven effective against the infectious diseases caused by antibiotic-resistant bacteria [4, 13].

A suitable carrier protein must be a good immune enhancer and has sufficient number of reactive residues for conjugation with linkers and immunogens. Bovine serum albumin (BSA), ovalbumin (OVA), keyhole limpet hemocyanin (KLH) and myoglobin are generally used in this area of research. BSA (MW ~ 66 kDa) contains 59 lysine residues, among them, 30 lysine residues are exposed outside for facile derivatization. OVA (MW ~ 44 kDa), the main protein in egg white, contains 20 lysine residues that are suitable to chemical modification. Both BSA and OVA have been utilized as carrier proteins in vaccine development, at least in the preliminary tests, because they are readily available. The carrier proteins derived from bacteria, such as diphtheria toxoid (DT), cross-reacting material 197 (CRM197), tetanus toxoid (TT), meningococcal outer-membrane protein complex (OMPC) and the genetically detoxified recombinant *Pseudomonas aeruginosa* exotoxin A, have also been employed to construct conjugate vaccines [14].

In this study, we further investigated the use of bacterial flagellin (FliC) as a self-adjuvanting protein carrier in preparation of the LFPS conjugate vaccine. FliC is the structural protein of bacterial flagellar filament, and regarded as a potent immunomodulatory agent. FliC can also trigger the T cell-dependent immune response via its intrinsic adjuvant property mediated by toll-like receptor 5 (TLR5) [15]. Moreover, the polymeric flagellin can directly stimulate B cell by cross linking with B-cell receptor (BCR) [16]. By co-localization of antigen and adjuvant to antigen presenting cells (APC), such antigen−adjuvant conjugate can elicit a co-delivery effect [17, 18] to convey enhanced immune responses. Therefore, using flagellin as a carrier in conjugate vaccine may have advantages due to its dual function as the antigen and as adjuvant for TLR5 activation [19]. FliC has been utilized to conjugate with low immunogenic antigens, such as O-antigens [20], mucoid exopolysaccharide (MEP) [21] and cocaine analogs [22], for producing the conjugate vaccines against bacterial infection and drug abuse. The immunization with FliC or flagellar filament can induce protective immunity against bacterial infection [23]. The anti-FliC antibodies not only enable the phagocyte-dependent killing but also reduce the motility of bacteria, resulting in a good control of disease. Such vaccine approach by conjugation of bacterial polysaccharide with flagellin protein was previously demonstrated to elicit high opsonophagocytic antibodies and protect mice against lethal challenge with virulent nontyphoidal *Salmonella* [20].

To design glycan vaccine, one should take many factors into consideration, including the linker that connects polysaccharide to protein. Many types of linkers have been used in the glycan−protein conjugation, such as those equipped with succinimide ester, *p*-nitrophenyl ester, maleimide, squarate, glutadialdehyde, amine, oxime, acyl hydrazide and α-haloacetamide [24–26]. Bifunctional linkers can be adapted to connect LFPS and carrier proteins. An appropriate linker should be non-immunogenic that does not elicit strong immune response against itself or suppressing the response to the carbohydrate immunogen. Moreover, the linker should have adequate water solubility for the reaction involving water-soluble carbohydrates and proteins [25, 26]. However, the best linker structure and optimal length are not well defined.

We report here the production of *S.* Typhimurium glycan vaccines and their efficacy in protection of mice. The workflow of this study comprises (i) preparation of LFPS as immunogen, (ii) using the exposed Kdo residue as the unique conjugation site, (iii) comparing the efficacy of several linkers and carrier proteins, and (iv) immunizing mice to evaluate the antibody titers and vaccine efficacy.

## Methods

The general experimental parts and details of syntheses and analyses are described in Supporting Information.

### Preparation of *S.* Typhimurium lipid-A free polysaccharide (LFPS)

*S.* Typhimurium LPS (200 mg) was dissolved in 1% AcOH (10 mL) and stirred at 100 °C for 2 h. The mixture was dialyzed with 3.5 K membrane against H_2_O four times for 1, 2, 4, and 16 h. After ultracentrifugation at 150,000 *g* for 5 h, the pellet of lipid A was removed, and the supernatant was further subjected to ultracentrifugation at 150,000 *g* for 17 h. The supernatant was lyophilized to obtain the desired LFPS. Alternatively, the dialyzed sample was subjected to ultracentrifugation once and purification by gel chromatography to give LFPS.

### Representative procedure for conjugation of LFPS via decarboxylative amidation of the terminal Kdo

A suspension of *S.* Typhimurium LFPS (92.6 mg, 4.0 μmol, based on the average molecular weight of 23 kDa) in DMSO (2.5 mL) was sonicated at room temperature for 10 min to dissolve all solid particles. A solution of *N*-(6-aminohexyl)-2-nitrobenzenesulfonamide, linker A/Ns as the TFA salt, 14.4 mg, 35 μmol) in DMSO (0.5 mL) was added to a vial containing iodine (19.9 mg, 78.3 μmol) and Cs_2_CO_3_ (57.8 mg, 177 μmol). The mixture was added to the above-prepared DMSO solution of LFPS, and stirred at room temperature for 23 h under an atmosphere of argon. The reaction was quenched by addition of Na_2_S_2_O_3_ (24.8 mg, 156.9 μmol) with stirring at room temperature for 10 min to give a crude product of PS–A/Ns (**20**). Sodium thiophenolate (7.1 mg, 53.7 μmol) was added to the crude product **20**, and the mixture was stirred at room temperature for 3 h under an atmosphere of argon to remove the nosyl protecting group. The mixture was dialyzed with a mini-dialysis device (MWCO 3500 Da, Slide-A-Lyzer, ThermoFisher, MA, USA) against MeOH (30 min, 2×), MeOH/dH_2_O = 1:1 (30 min, 2×), and then dH_2_O (16 h). The retentate was lyophilized to give the PS–A/NH_2_ product (**21**) as a yellow oil (122 mg).

### Evaluation of hTLR5 activity by SEAP reporter cellular assay

A suspension of HEK-Blue hTLR5 cells (InvivoGen) was prepared at a concentration of 1.4 × 10^4^ cells/mL in the HEK-Blue Detection medium. Then the cell suspension was added to a 96-well plate (180 μL/well, ~ 25,000 cells per well) containing 50, 10 and 1 ng/mL of an indicated PS–linker–FliC conjugate, and incubated at 37 °C for 24 h; then the TLR5 activation was evaluated by the absorbance at 620 nm due to the SEAP-catalyzed hydrolysis of substrate. The data were presented as mean ± standard deviation (*n* = 5). The comparison of paired samples was performed by using Student’s t test.

### Mice immunization experiment

The animal study was performed in the laboratory animal center of Chang Gung University and compliance with the policy of animal care and use. Each group in the immunization experiments has 5 mice (BALB/c mice aged 6–8 weeks). BALB/c mice were immunized with the LFPS–protein conjugate (e.g. PS–A–B–BSA, **7a**) at a dose of 2.5 or 5 μg for four times on weeks 0, 2, 4 and 6. Sera were collected from immunized mice by eye-bleeding method (bleeding from the retroorbital venous plexus of mice) before immunization and after immunization on week 8. For the first immunization on week 0, the LFPS–protein conjugate was mixed with equal volume of Freund’s complete adjuvant (Sigma). For the rest of immunization, the LFPS–protein conjugate (or LFPS) was mixed with equal volume of Freund’s incomplete adjuvant (Sigma).

Alternatively, BALB/c mice aged 6–8 weeks were randomly assigned to one control group (*n* = 5) and three experimental groups of 10 mice. Mice were immunized with the LFPS–protein conjugate (e.g. PS–A–B–FliC, **7c**) at a dose of 2.5 μg by subcutaneous administration for three times on weeks 0, 2, and 4. No additive adjuvant was used for both initial vaccination and booster immunization. Sera were collected from immunized mice by retro-orbital bleeding method (bleeding from the retro-orbital venous plexus of mice) before immunization and after immunization on week 6.

### Serum antibody titer test

Anti-LPS antibodies in BALB/c mice were determined by ELISA. Mouse sera were taken from immunized BALB/c mice by eye-bleeding method. ELISA plates were coated with 3 μg antigen (e.g. purified *S.* Typhimurium LPS) at 4 °C for 16 h. The coated plates were blocked with 2% BSA at room temperature for 2 h. Plates were washed 3 times in PBS containing 0.05% Tween 20 (PBST-20). Then appropriately diluted solution of mouse serum was added to the well. Plates were incubated for 2 h at room temperature. After washing as described above, horseradish peroxidase (HRP)–conjugated goat anti-mouse antibody (Millipore) was added to each well and the plates were incubated at room temperature for 2 h. Following the washing steps as described above, a 3,3′,5,5′-tetramethylbenzidine (TMB) solution was added to the well for 20 min, and the reaction was stopped with 2 M H_2_SO_4_. The optical density at 450 nm of each well was measured.

### Bacterial challenge test

The immunized mice received oral or intravenous challenge with virulent pathogen (e.g. *S.* Typhimurium SL1344) equivalent to 1 × 10^6^ (LD_50_; oral challenge), 5 × 10^6^ CFU (lethal dose; oral challenge) and 1 × 10^3^ CFU (intravenous challenge) on day 14 after the last immunization. The mortality was recorded daily for 21–28 days.

### Statistical analysis

The differences of antibody titers between pre- and post-immunization were analyzed using Paired t-test. The Kaplan-Meier method and the log-rank test were used to compare the survival of mice in the immunized and control groups. The survival times were also modeled using Cox proportional hazards regression to estimate the hazard ratios and protective factors of vaccination with distinct vaccines compared to the controls. Data analysis was performed using IBM SPSS Statics (version 24).

## Results

### Preparation and fractionation of LFPS

We first removed the lipid-A moiety from LPS and exposed the Kdo as a unique point of linkage. Using a modified Darveau−Hancock method [27], the LPS was isolated from *S.* Typhimurium cells and then heated in 1% AcOH aqueous solution at 100 °C for 2 h to cleave the linkage between Kdo and lipid A (Figure S1B). The hydrolytic product was dialyzed with 3.5 kD MWCO membrane. After ultracentrifugation (150,000 *g*) for 5 h, the supernatant containing polysaccharide was saved and lipid-A pellets were discarded. The supernatant was lyophilized to obtain LFPS with a median molecular weight of 23 kDa [28, 29]. This procedure was applicable to obtain a large amount (e.g. 800 mg) of LFPS sample. The quantity of saccharides was routinely determined by the absorbance at 490 nm in phenol−sulfuric acid assay (PSA) [30] with a modified procedure [31]. In cytotoxicity tests, while LPS induced high levels of cytokines, such as TNF-α and IL-6, the LFPS product only induced very small amounts of cytokines (Figure S2). The length and number of polysaccharide immunogens may influence the efficacy of glycan−protein conjugate vaccines [32, 33]. We thus fractionated *S.* Typhimurium LFPS by size-exclusion chromatography (SEC) to obtain a high-mass portion (fraction 1) in a median molecular weight of 85.8 kDa (Figure S3A).

### Design of LFPS–linker–protein conjugates

**Fig. 1:**
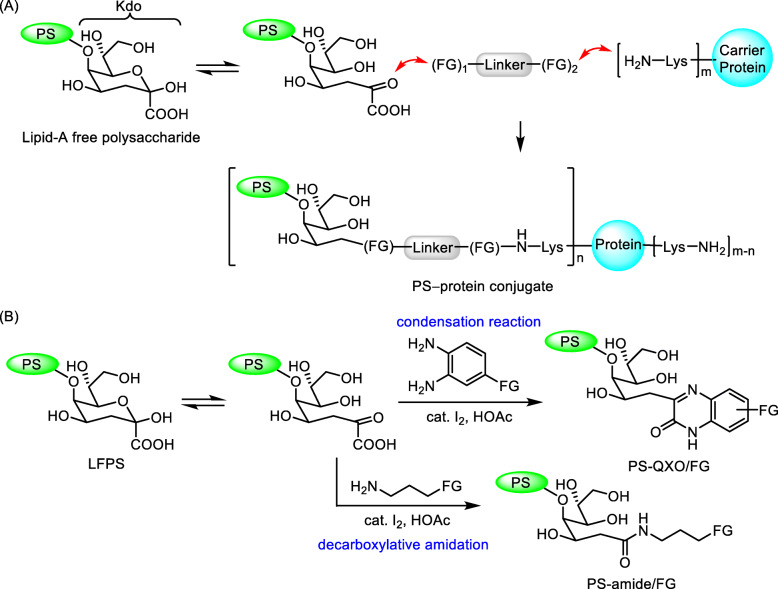
Design of polysaccharide–protein vaccine. **a** Diagram for connecting the terminal Kdo of LFPS and the lysine residues of carrier protein. The drawings of PS, linker and protein are not proportional to their relative sizes. **b** Transformation of Kdo-terminated polysaccharide into quinoxalinone (PS–QXO/FG) and amide (PS–amide/FG) linked products via the condensation reaction with *o*-phenylenediamine and the decarboxylative amidation with amine, respectively

We chose to react Kdo of LFPS with differently designed bifunctional linkers, and then react with the lysine residues of carrier protein to form the LFPS–linker–protein conjugates (Fig. 1a). The α-ketoacid group in the terminal Kdo saccharide was specifically attached to a chosen linker. Figure 1b shows our methods for conjugation of the α-ketoacid type of carbohydrate residues [34–37]. The condensation reaction of α-ketoacid with *ortho*-phenylenediamine yields the quinoxalinone (QXO) derivative [35, 36], whereas the iodine-promoted reaction with amine affords an amide product [34, 37]. The formation of QXO and amide derivatives was irreversible and relatively robust, compared to the conjugation method devised by oxime formation with hydroxylamine-modified BSA [38].

**Fig. 2:**
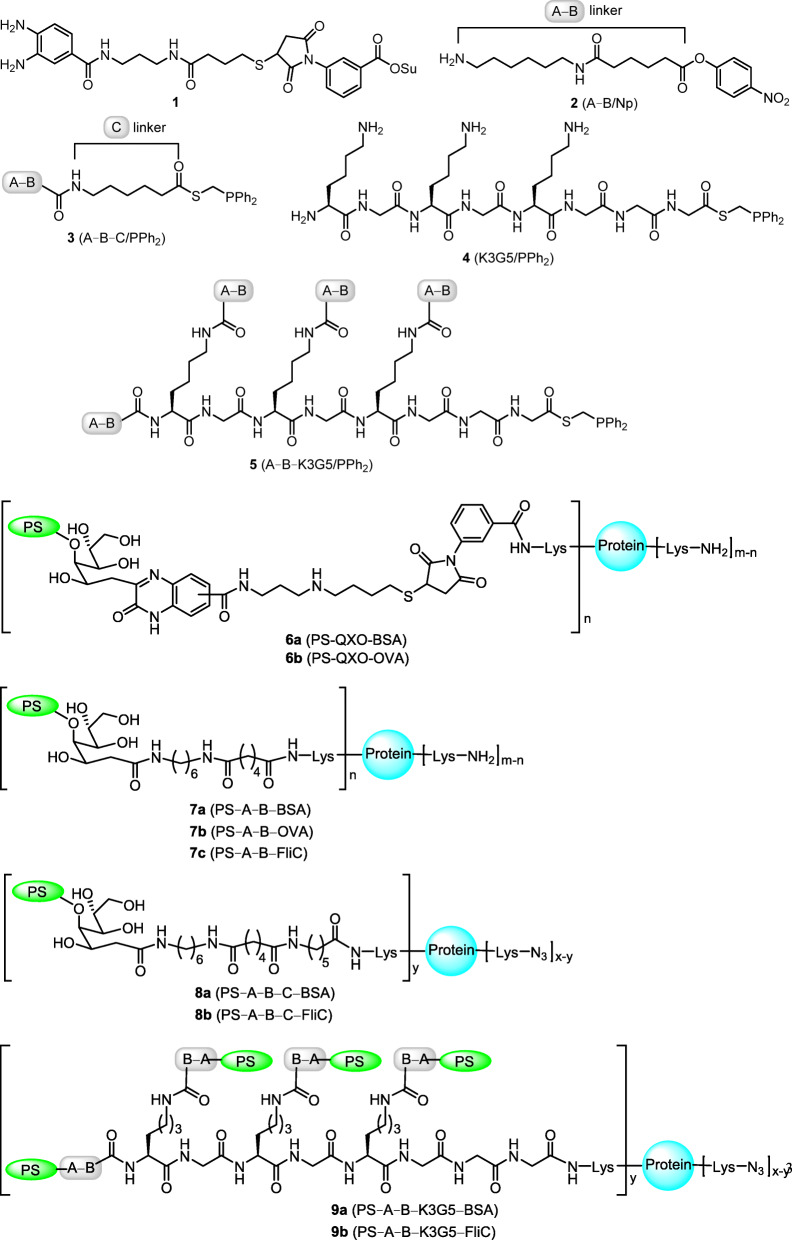
Designed bifunctional linkers 1–5 and polysaccharide–protein conjugates 6a–9b

Figure 2 shows our designed bifunctional linkers **1**–**5**, which bear either amine or *o*-phenylenediamine for conjugation with Kdo of LFPS. At the other terminal are activated esters for conjugation to lysine residues of carrier protein. For example, the carrier protein can be modified via coupling of lysine residues with the hinged *p*-nitrophenyl (Np) ester group on the A–B linker in structure **2**. Alternatively, traceless Staudinger ligation enables the coupling reaction of azido-modified lysine residue with phosphino (thioester) group on linkers **3**–**5**. Linker **4** contains a lysine-rich octapeptide KGKGKGGG (designated as K3G5 in this paper), while an extended linker **5** is devised for incorporation of multiple LFPS immunogens by connection of **4** and A–B linkers.

In our first approach, the PS − protein conjugates **6a** and **6b** having QXO linker were synthesized. The detailed procedures for the syntheses of PS–protein vaccines are described in Scheme S1. We later learned that heterocycles (QXO and 3-thiosuccinimide in this case) are also highly immunogenic, and may interfere with the desired immune response [39, 40].

We then used decarboxylative amidation to obtain the PS–protein conjugates **7a**–**9b** containing straight-chain linkers (Scheme S2–S4) [34]. By this method, the immuno-compatible amide groups were used in the linkers. In brief, LFPS was stirred with excess amount of an appropriate amine linker in DMSO solution at room temperature for 23 h in the presence of iodine and Cs_2_CO_3_ to furnish the decarboxylative amidation reaction. For the synthesis of conjugates **7a**–**7c**, the lysine residues of carrier protein (BSA, OVA and FliC) were used in the coupling reactions with the hinged *p*-nitrophenyl (Np) ester group on linker. The coupling reaction was usually performed in phosphate buffer (pH 7.4), albeit the efficiency was appreciably increased by adding 10% of DMF as a cosolvent to improve the solubility of substrates. For the synthesis of conjugates **8a**–**9b**, traceless Staudinger ligation enabled the coupling reaction of the azido-modified lysine residue with the phosphino (thioester) group on linker to form an amide bond [41].

FliC (MW 30–60 kDa) has multiple lysine residues for derivatization. *S.* Typhimurium FliC (UniProt coding: P06179) contains 28 lysine residues that are distributed to all the D0, D1, D2 and D3 domains (Figure S4). The D0 and D1 domains of FliC are conserved and form a spoke region inside the assembled tubular structure of flagellar filament [42]. The D1 domain of FliC provides the most binding sites for recognition with TLR5 via hydrogen bonds and salt-bridge interactions [42–44]. The D0 domain is unrelated to TLR5 binding but required to activate the flagellin-mediated cellular activity [42]. In contrast, the hypervariable D2 and D3 domains are arranged outside the flagellar filament [44], away from the binding interface of the FliC−TLR5 complex [43]. FliC monomers are directly subjected to chemical modification with imidazole-1-sulfonyl azide by the conventional method [45], so that the lysine residues in all domains may be modified in non-selective manner. Since such excessive modification of FliC may cause steric hindrance and interfere with its binding to TLRs as to reduce its adjuvant effect, a site-selective modification method was also carried out [46]. FliC monomers are first treated in high concentration of Na_2_SO_4_ solution to afford flagellar filaments, so that only the lysine residues of the exposed D2 and D3 domains can be easily modified. For the synthesis of PS–FliC conjugate **7c** (Scheme S2). *S.* Typhimurium FliC monomers were conjugated with PS via the coupling of the ε-NH_2_ groups of lysine residues with the Np ester group on the linkers in a non-selective manner. On the other hand, selective modification of amino groups on flagellar filament in high salt condition was applied to prepare the azido-FliC for subsequent synthesis of conjugates **8b** (Scheme S3) and **9b** (Scheme S4).

### Chemical analysis of LFPS–linker–protein conjugates

**Fig. 3:**
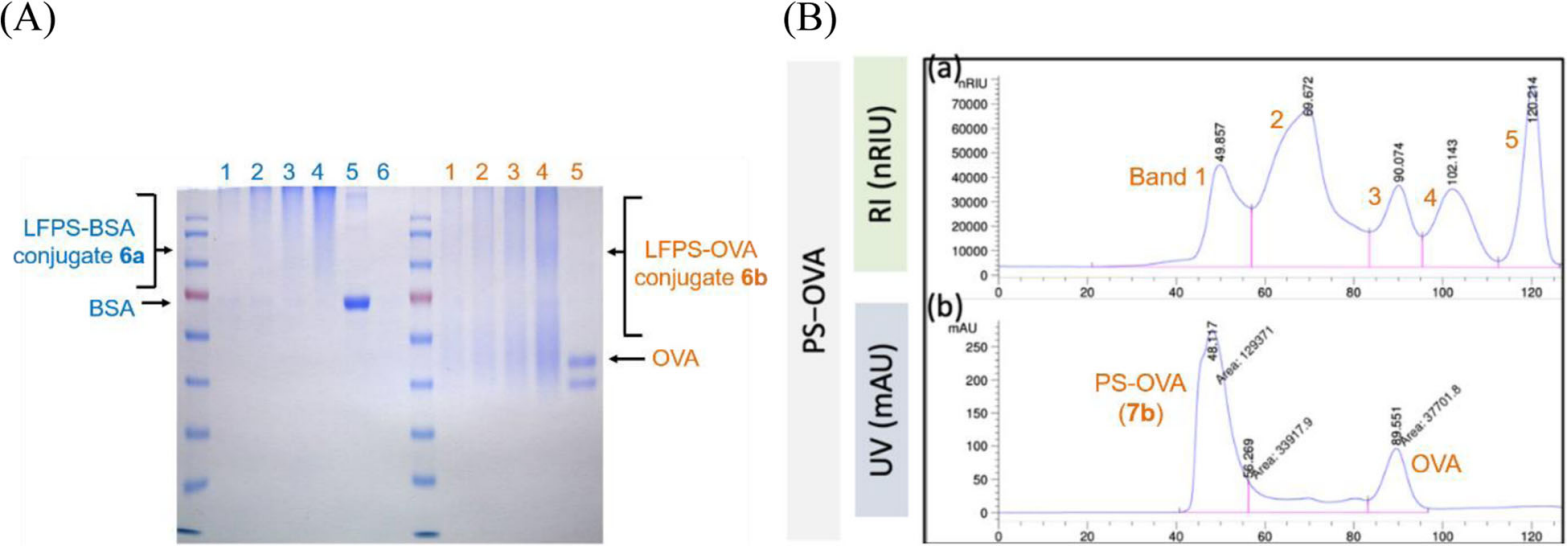
Chemical analysis of PS–protein conjugates. **a** SDS-PAGE of *S.* Typhimurium PS–BSA (6a) and PS–OVA (6b) conjugates using Coomassie brilliant blue. Lanes 1–4 are PS–protein conjugate in 4, 10, 20 and 40 μg, respectively. Lane 5 is unmodified protein (2 μg), and lane 6 is unmodified LFPS (40 μg). **b** FPLC diagram of PS − A–B–OVA (7b) conjugate, which was prepared from *S.* Typhimurium fraction-1 LFPS. Column: Superdex-200 pg; eluent: PBS at a flow rate of 1 mL/min. **a** and **b** are the diagrams using RI and UV-vis (λ = 280 nm) detectors, respectively

The LFPS−protein conjugates **6a** and **6b** appeared as diffuse bands in sodium dodecyl sulfate polyacrylamide gel electrophoresis (SDS-PAGE), indicating the heterogeneity of shape and mass-to-charge ratio (Fig. 3a). The smear tailing bands might be also related to varied numbers of PS attached to a carrier protein. The staining color became deeper as the loading quantity increased; however, some products with large molecular mass were unable to enter the stacking gel (Figure S5). In this case, mass spectrometry is inadequate for analysis of the synthesized PS − protein conjugates due to their high molecular weights (> 50 kDa) and low ionization intensity.

Fast performance liquid chromatography (FPLC) is a good method for glycoprotein analysis. Protein and its conjugates are detected by absorption at 280 nm (A_280_), whereas polysaccharide is UV transparent. For example, fraction-1 LFPS having a median molecular weight of 85.8 kDa was used to synthesize the PS − A–B–OVA conjugate **7b**. FPLC of the crude product on a Superdex-200 pg column (Fig. 3b) revealed that conjugate **7b** occurred at band 1 (*t*_R_ ≈ 48.5 min) and unreacted OVA at band 3 (*t*_R_ ≈ 90 min). Based on the integrated area of UV signals for bands 1 and 3, about 64% OVA was converted to PS − A–B–OVA conjugate. Comparison of refractive index (RI) and UV signals suggests band 2 could be attributable to partially degraded PS with protein contaminant, and bands 4 and 5 might be saccharide fragments. The PS − OVA conjugate **7b** (band 1) was isolated by FPLC on a size exclusion chromatography column (FPLC–SEC), and the contents of polysaccharide (342.2 μg/mL) and protein (123.0 μg/mL) were determined by PSA method and bicinchoninic acid assay (BCA), respectively. The PS/protein molar ratio was calculated to be 1.4, indicating that 1 to 2 PS chains were linked to each OVA. By similar procedures, the conjugation numbers for other synthesized PS–protein conjugates were deduced (Table 1). FPLC diagrams of other PS–protein conjugates (**7a**, **7c**, **8a**–**9b**) are shown in Figure S6.

**Table 1:** Carbohydrate-to-protein molar ratio and estimated conjugation number of the synthesized PS − protein conjugates

Entry	PS–protein conjugate^*a*^	PS μg/mL (μM)^*b*^	Protein μg/mL (μM)^*c*^	Conjugation number^*d*^
1	**7a** (BSA)^*e*^	168.7 (1.97)	62.4 (0.95)	2–3 (2.1)^*d*^
2	**7a** (BSA)^*f*^	98.2 (4.27)	13.2 (0.20)	20–23 (21.8)^*d*^
3	**7b** (OVA)^*e*^	342.2 (3.99)	123.0 (2.80)	1–2 (1.4)^*d*^
4	**7c** (FliC)^*f, g*^	168.4 (7.32)	24.0 (0.47)	14–17 (15.6)^*d*^
5	**8a** (BSA)^*f*^	94.1 (4.09)	13.8 (0.21)	18–21 (19.5)^*d*^
6	**8b** (FliC)^*f, h*^	235.5 (10.24)	83.1 (1.63)	5–7 (6.3)^*d*^
7	**9a** (BSA)^*f*^	95.4 (4.15)	15.2 (0.23)	17–19 (18.0)^*d*^
8	**9b** (FliC)^*f, h*^	221.0 (9.61)	96.8 (1.90)	4–6 (5.1)^*d*^

^*a*^ PS−protein conjugates prepared from *S.* Typhimurium LFPS, and isolated by FPLC−SEC

^*b*^ Data derived from PSA assay by calibration of the absorbance at 490 nm

^*c*^ Data derived from BCA assay by calibration of the absorbance at 562 nm based on the molecular weights of OVA (44 kDa), BSA (66 kDa) and FliC (51 kDa)

^*d*^ Estimated number of LFPS on each PS–protein conjugate. Data in parenthesis are calculated from the molar ratio of PS over protein

^*e*^ The PS−protein conjugate was prepared from fraction-1 LFPS with a median molecular weight of 85.8 kDa

^*f*^ The PS−protein conjugate was prepared from unfractionated LFPS with average molecular weight of 23 kDa

^*g*^ Conjugate **7c** was prepared by the coupling reaction of PS−A−B/Np with FliC

^*h*^ The azido-modified FliC was prepared by site-selective method, and used in synthesis of **8b** and **9b**

### hTLR5 a by FliC-based self-adjuvanting vaccines

**Fig. 4:**
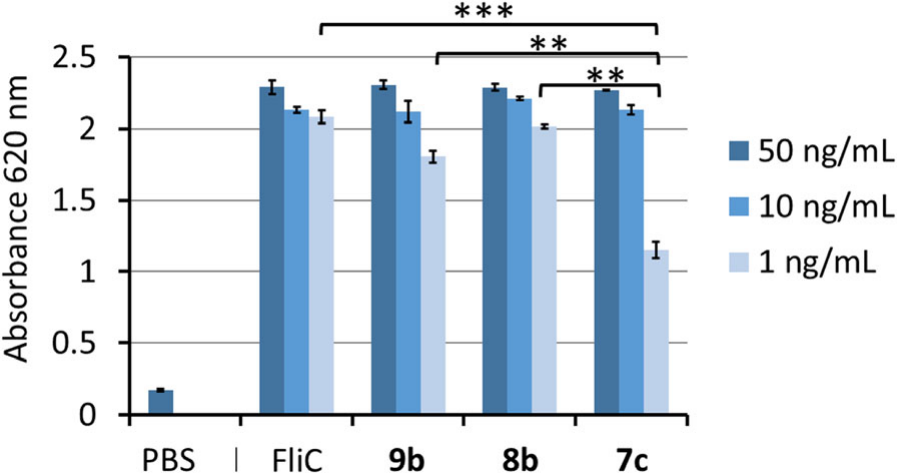
hTLR5 activation by PS–linker–FliC conjugates 7c, 8b and 9b. Unmodified FliC was used to prepare 7c, whereas site-selective azido-modified FliC was used to prepare 8b and 9b. The concentration is estimated by the sum of the mass of carbohydrates and proteins. Native FliC is positive control, and 1× PBS is negative control. ** *p* < 0.01 and *** *p* < 0.001. The data are presented as mean ± standard deviation (*n* = 3). A comparison of paired samples was performed by using Student’s t-test

The capability in TLR5 activation was evaluated by a secreted embryonic alkaline phosphatase (SEAP) reporter assay using the HEK-Blue hTLR5 cell line (InvivoGen) derived from the HEK293 cells with co-transfection of hTLR5 and SEAP genes. Figure 4 shows that the PS-FliC conjugates enable to activate hTLR5 even at a low incubation concentration of 1 ng/mL. The conjugates **8b** and **9b**, which were prepared by site-selective modification of FliC, exhibited the similar potency as native FliC for hTLR5 activation. Though conjugate **7c** prepared from unmodified FliC showed comparable ability in hTLR5 activation at 10 and 50 ng/mL concentrations, it was inferior at 1 ng/mL concentration (*p* < 0.01).

### Immunization and challenge experiments

**Fig. 5:**
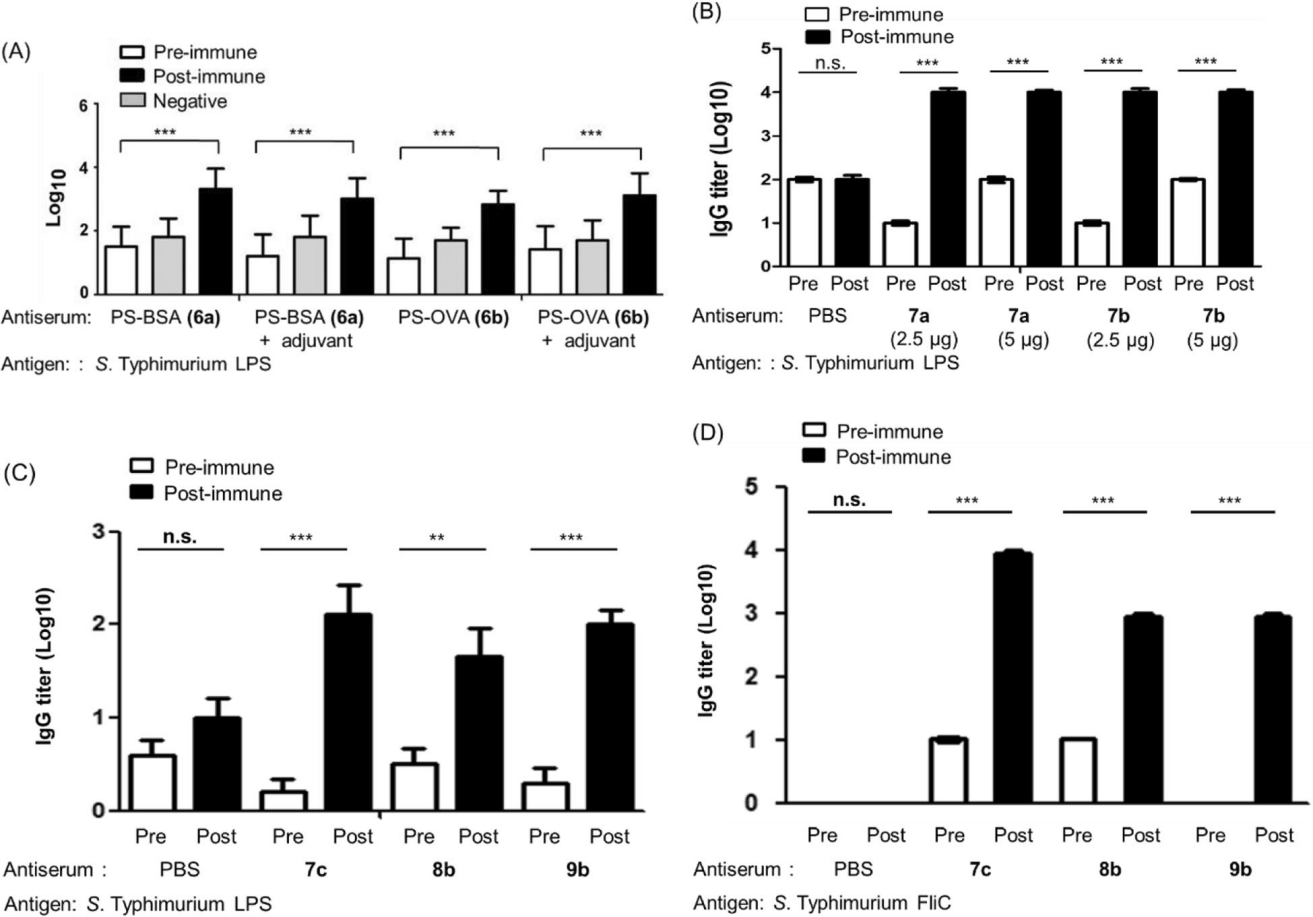
Endpoint titers of immune serum IgG specific to *S.* Typhimurium LPS (A–C) or FliC (D). BALB/c mouse (*n* = 5 for each group) was immunized: (**a**) using PS–QXO–BSA (6a) or PS–QXO–OVA (6b) at 2.5 μg dosage. Freund’s complete adjuvant was injected on week 0, and the Freund’s incomplete adjuvant was injected on weeks 2, 4 and 6. (**b**) using PS–A − B − BSA (7a) or PS–A − B − OVA (7b) prepared from fraction-1 LFPS at 2.5 μg or 5.0 μg dosage, and (**c**) using PS–A − B − FliC (7c), PS − A − B − C − FliC (8b) or PS − A − B − K3G5 − FliC (9b) at 2.5 μg dosage. The data were derived from two independent experiments. PBS was negative control. The cut-off value was defined as two-fold of the absorbance reading of negative control. * *p* < 0.05, ** *p* < 0.01 and *** *p* < 0.001. The data are presented as mean ± standard deviation (*n* = 5). A comparison of paired samples was performed by using Paired t-test

Initially, we aimed to determine the appropriate dosage and adjuvant for mice experiments. The PS − QXO–protein immunogen **6a** (or **6b**) was administered to BALB/c mice by subcutaneous (s.c.) injection. The dosage of 2.5 μg per mouse was applied for four times on weeks 0, 2, 4 and 6 to induce antibody. Sera were collected from immunized mice by eye-bleeding method before and after immunization on week 10. Blood of mouse was drawn on week 10 to determine the antibody titer by enzyme-linked immunosorbent assay (ELISA) against the LPS antigen. To evaluate the effect of adjuvant, the Freund’s complete and incomplete adjuvants were used in the experiments. Freund’s complete adjuvant was injected on week 0, and the Freund’s incomplete adjuvant was injected on weeks 2, 4 and 6. For comparison, blood of mouse before immunization was also examined. Figure 5a shows the ELISA results of the immunization experiments using the PS − BSA (**6a**) and PS − OVA (**6b**) conjugates as vaccines. We found that injection of the PS–protein conjugate at a dosage of 2.5 μg was sufficient to elicit serum IgG specific to *S.* Typhimurium LPS. No obvious difference in the antibody titer was observed when the injection dosage was increased from 2.5 μg to 5 μg. The effect between Freund’s complete and incomplete adjuvants was insignificant.

In the next experiment, mice were subcutaneously injected with PS − A − B − BSA (**7a**) and PS − A − B − OVA (**7b**) vaccines, which were prepared from fraction-1 LFPS, at the dosage of 2.5 μg or 5.0 μg without using adjuvant. After three injections at an interval of every 2 weeks, blood of the mouse was drawn on week 7 to determine the antibody titer against *S.* Typhimurium LPS. The IgG titers of four immunized groups (Fig. 5b) revealed that both vaccines induced the desired immune response in mice. The endpoint titer of serum IgG post immunization with **7a** and **7b** was 100-fold higher than the titer elicited by the endogenous antibody. Again, no apparent difference in antibody titer was observed as the dosage increased from 2.5 μg to 5 μg. By the similar protocol, the conjugates **7a** and **7b** were also prepared from unfractionated *S*. Typhimurium LFPS, and used to immunize mice. Though the high-mass portion of LFPS appeared to benefit antibody induction (Figure S3B), immunogen **7a** (or **7b**) prepared from fraction-1 and unfractionated LFPS showed equal efficacy in induction of IgG antibodies (compared Fig. 5b with Figure S8B).

The mice immunized with three different FliC-based vaccines (**7c**, **8b** and **9b**) all elicited significant immune responses against *S.* Typhimurium LPS (Fig. 5c) and FliC (Fig. 5d). Among them, PS − A − B − FliC (**7c**) vaccination induced the strongest immune response against LPS and FliC.

**Fig. 6:**
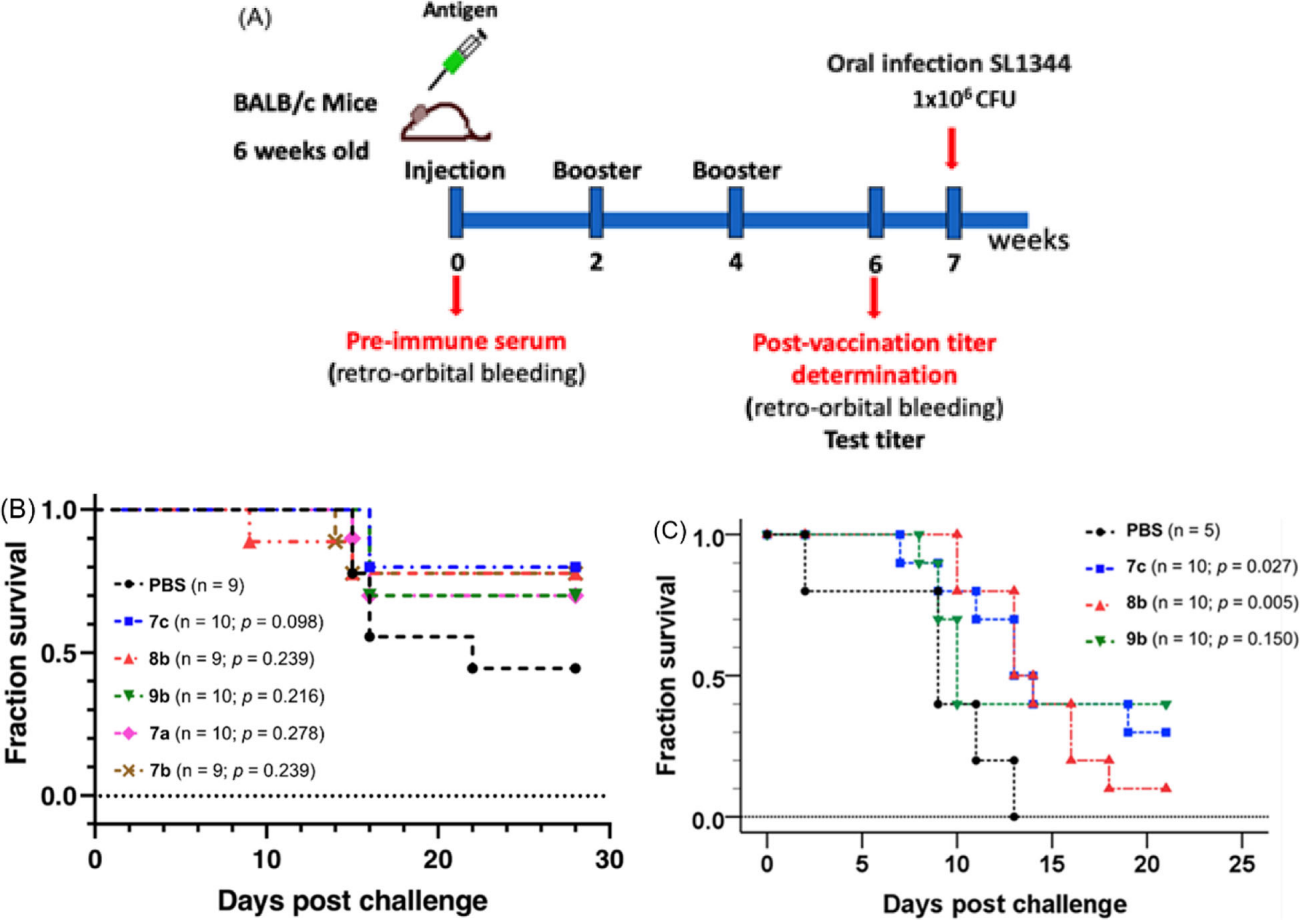
Immunization scheme (**a**) and Kaplan-Meier survival curves (**b**&**c**) for the challenge experiments of mice immunized with PS–protein vaccines. Each group was conducted with 9 or 10 BALB/c mice, which were s.c. administered with the vaccine at a dosage of 2.5 μg according to the immunization scheme. The immunized mice were then orally challenged with 1 × 10^6^ CFU LD_50_ (**b**) or lethal dose (**c**) of *S.* Typhimurium SL1344 bacteria. The data were derived from two independent experiments. PS was unfractionated LFPS prepared from the LPS of *S.* Typhimurium ATCC7823. The log-rank test was used to compare the survival of mice in the immunized and control groups

The O-antigens of *Salmonella* serotypes within the same serogroup are highly conserved. Both SL1344 and ATCC7823 belong to *S.* Typhimurium, a serotype of serogroup B. According to Kauffmann–White classification scheme, *S.* Typhimurium SL1344 and ATCC7823 have the same O-antigen profile of (1,4,5,12). We thus used SL1344, which has the same O-antigen profile as ATCC7823, to challenge the immunized mice. As transmission of *Salmonella* bacteria proceeds through an orofecal route, we conducted the challenge experiments by oral gavage of pathogens on week 7 to the mice immunized with PS–protein vaccines **7a**, **7b**, **7c**, **8b** and **9b**. Figure 6b shows the immunization scheme and Kaplan-Meier survival curves of mice challenged with half maximal lethal dose (LD_50_) of *S.* Typhimurium. The results are collected in Table 2. At day 28 post administration with PBS as a control, 4 out of 9 mice survived (44.4%). Immunization with the PS–protein vaccines **7a**, **7b**, **7c**, **8b** and **9b** all improved the survival rate (70–80%). The log-rank test was used to compare the survival of mice in the immunized and control groups. Among them, the PS–A–B–FliC vaccine (**7c**) appeared to exhibit the best efficacy of 74.1% according to Cox proportional hazard model [47], though the *p* value of 0.098 cannot be considered statistically significant.

**Table 2:** Survival of BALB/c mice after vaccination and challenge with LD_50_ of *S.* Typhimurium SL1344^*a*^

entry	PS–protein vaccine^*b*^	mortality at day 28^*c*^	survival rate (%)	hazard ratio (95% CI)^*d*^	*p*-value^*e*^	vaccine efficacy (%)^*f*^
1	PBS	5/9	44.4	–	–	–
2	**7a** (BSA)	3/10	70.0	0.435	0.278	56.5
3	**7b** (OVA)	2/9	77.8	0.395	0.239	60.5
4	**7c** (FliC)	2/10	80.0	0.259	0.098	74.1
5	**8b** (FliC)	2/9	77.8	0.395	0.239	60.5
6	**9b** (FliC)	3/10	70.0	0.378	0.216	62.2

^*a*^ Mice were orally challenged with 1 × 10^6^ CFU (LD_50_) *S.* Typhimurium SL1344 bacteria

^*b*^ The PS − protein conjugates **7a**–**9b** were prepared from unfractionated LFPS of 23 kDa

^*c*^ Number of dead mice over total number of test mice

^*d*^ Cox proportional hazard model results for the risk of death after challenge of *S.* Typhimurium SL1344 bacteria. CI is confidence interval

^*e*^ Comparison of survival distribution with the PBS group (negative control). The statistics are performed by log-rank test

^*f*^ Vaccine efficacy = (1 – hazard ratio) × 100%

In another experiment, mice were challenged with lethal dose of *S.* Typhimurium (Fig. 6c). As shown in Table 3, no mouse in the control group administrated with PBS survived at day 13. In comparison, 30% of mice survived at day 21 by immunization with PS−A−B−FliC (**7c**, *p* = 0.027), and 10% of mice survived by immunization with PS–A–B–C–FliC (**8b**, *p* = 0.005). The protective effect of **7c** is statistically significant. Immunization of PS–A–B–K3G5–FliC vaccine (**9b**) also provided protection for mice against the challenge of *S. typhimurium* SL1344, showing 40% survival rate at day 21 (*p* = 0.150).

**Table 3:** Survival of BALB/c mice after vaccination and challenge with a lethal dose of *S.* Typhimurium SL1344

entry	PS–protein vaccine	mortality at day 21	survival rate (%)	hazard ratio (95% CI)	*p*-value^*e*^	vaccine efficacy (%)^*f*^
1	PBS	5/5	0	–	–	–
2	**7c** (FliC)	7/10	30	0.273	0.027	72.7
3	**8b** (FliC)	9/10	10	0.344	0.005	65.6
4	**9b** (FliC)	6/10	40	0.285	0.150	71.5

## Discussion

According to the above-described results, the efficacy of glycan−protein conjugate vaccines is affected by the length and number of polysaccharide immunogen, as well as the types of linker and carrier protein. *S.* Typhimurium lipopolysaccharide undergoes a selective acidic hydrolysis to give LFPS with exposure of the terminal Kdo saccharide as a specific point to attach *ortho*-phenylenediamine and amine linkers via condensation reaction and oxidative amidation, respectively, to give the PS − protein conjugates **6a**/**6b** and **7a**–**9b**. Modification of LFPS at the terminal Kdo preserves the remainder of the sugar chain, including O-antigen and core polysaccharide. This approach has advantage over other random activation methods that may interfere with the immune recognition of carbohydrate antigen. Compared to the conjugation method devised by oxime formation with hydroxylamine-modified BSA [38], the formation of QXO and amide derivatives is irreversible and relatively robust. The method of reductive amination commonly used for linkage with aldose is not suitable for the α-ketoacid group of Kdo in LFPS due to low reactivity.

The efficiency of ligation can be inferred from the conjugation number. Compared entries 1 and 3 in Table 1, BSA has more accessible lysine residues than OVA to conjugate with fraction-1 LFPS chains. The conjugation number of unfractionated LFPS to BSA greatly increases (entries 2, 5 and 7), indicating that smaller PS fragments in unfractionated LFPS have higher conjugation efficiency than fraction-1 LFPS of high molecular weight. Comparison of non-selective method (entry 4) with site-selective method (entries 6 and 8) for modification of FliC shows that the former renders more azido-modified lysine residues for conjugation with LFPS. In our original design, more PS chains are expected to attach to the K3G5-incorporated linker. However, the steric demanding substrate of PS−A−B−K3G5/PPh_2_ derived from linker **5** is less efficient in the Staudinger ligation with the azido-modified BSA and FliC proteins for preparation of the conjugates **9a** and **9b**, than with the conjugates **8a** and **8b** that are prepared from PS−A−B−C/PPh_2_.

The PS-FliC conjugates **8b** and **9b**, which are prepared by using the FliC with site-selective modification, enable to activate hTLR5 as potent as native FliC. In contrast, the PS–FliC conjugate **7c** prepared from unmodified FliC has inferior ability in hTLR5 activation at a low incubation concentration of 1 ng/mL. This result may reflect that some lysine residues in the D0 and D1 domains are modified with LFPS and thus detrimental to TLR5 activation. However, the self-adjuvant activity of conjugate **7c** may be underestimated because it contains a relatively low level of FliC (compared entry 4 with entries 6 and 8 in Table 1), and too many polysaccharides on FliC may be interfering with self-adjuvant activity.

The mice experiments show that injection of the PS–protein conjugate at a dosage of 2.5 μg is sufficient to elicit serum IgG specific to *S.* Typhimurium LPS. Although the PS−protein conjugates **6a** and **6b** elicit high titer of immunogenicity in mice, the serum antibodies also show appreciable affinity to a synthesized QXO linker (Figure S7A). This result indicates that the QXO linker in the PS−protein conjugates **6a** and **6b** may interfere with the desired immune response. On the other hand, the ELISA experiments using a synthesized biotin−A−B linker indicate that the A−B linker only exhibits low immunogenicity (Figure S7B). Thus, the IgG antibodies elicited by **7a** and **7b** mainly recognize the polysaccharide immunogen. As immunogen **7a** (or **7b**) prepared from fraction-1 and unfractionated LFPS shows equal efficacy in induction of IgG antibodies, the results indicate that the conjugate vaccine with clustered high-mass PS immunogen can provide multivalent interactions with immunocytes [48–50], whereas unfractionated LFPS can mimic the heterogeneous bacterial surface to elicit potent immune response. Among all the synthesized vaccines in this study, PS−A−B−FliC (**7c**) vaccination induces the strongest immune response against LPS and FliC. This result is consistent with that **7c** has the high conjugation number (14–17) of unfractionated LFPS onto FliC (Table 1, entry 4), so that the abundant and heterogeneous decorations of polysaccharide immunogens can facilitate the production of IgG antibodies. The high vaccine efficacy of **7c** can be attributable to the combined PS immunogenicity and FliC adjuvant effect.

## Conclusions

*S.* Typhimurium is a foodborne pathogen that causes numerous diarrheal infections. As drug-resistant *S.* Typhimurium emerges, development of nontyphoidal *Salmonella* vaccines is needed to control and prevent this infectious disease. *S.* Typhimurium is a Gram-negative bacterium that possesses LPS as the recognition target of immune cells. We first removed the toxic lipid-A moiety from LPS to obtain LFPS as the polysaccharide immunogen, which contains an exposed Kdo as a unique point to link carrier protein. We designed several bifunctional linkers to connect LFPS and carrier protein for preparation of the conjugate vaccines **6a**–**9b** (Fig. 2). The linker comprising an *o*-phenylenediamine moiety underwent the condensation reaction with Kdo to form quinoxalinone, while the amine linker is specifically attached to LFPS via decarboxylative amidation. In addition to BSA and OVA, the *S.* Typhimurium flagellin was also used as a self-adjuvanting protein carrier. FPLC equipped with UV and RI detectors was especially useful in chemical analysis of the PS–protein conjugates, and the contents of polysaccharides and protein were routinely determined by phenol–sulfuric acid assay and bicinchoninic acid assay, respectively.

We carried out mice immunization experiments using the synthetic PS–protein vaccines, and determined the antibody titers by ELISA. Injection of the PS–protein vaccines at a dosage of 2.5 μg was sufficient to elicit serum IgG specific to *S.* Typhimurium LPS. Our study indicated that straight-chain amide linkers in conjugates **7a**–**9b** did not interfere with the desired immune response, while the QXO heterocycle in conjugates **6a** and **6b** might complicate the antibody titer. The synthetic PS–protein vaccines **7a** and **7b** derived from either unfractionated LFPS or the high-mass portion showed equal efficacy in induction of IgG antibodies. This result might reflect a compromise between the cluster effect of high-mass PS immunogen and the display of diversified glycans similar to that on bacterial surface.

By the similar protocol, the conjugates **7a** and **7b** were also prepared from unfractionated *S*. Typhimurium LFPS, and used to immunize mice. Though the high-mass portion of LFPS appeared to benefit antibody induction (Figure S3B), immunogen **7a** (or **7b**) prepared from fraction-1 and unfractionated LFPS showed equal efficacy in induction of IgG antibodies (compared Fig. 5B with Figure S8B). These results indicated that the conjugate vaccine with clustered high-mass PS immunogen might provide multivalent interactions with immunocytes [47–49], whereas unfractionated LFPS might mimic the heterogeneous bacterial surface to elicit potent immune response. The LFPS conjugate vaccines **7c**, **8b** and **9b** using FliC as carrier protein also showed high potency in hTLR5 activation even at a low incubation concentration of 1 ng/mL. This effect was beneficial to enhance immune responses of the PS–FliC vaccines. The challenge experiments by oral gavage of *S*. Typhimurium pathogen, immunization of mice with the PS–A–B–FliC conjugate (**7c**), without external adjuvant, exhibited the best vaccine efficacy of 74.1% with 80% mice survival rate. This good vaccine efficacy may be attributable to the combined PS immunogenicity and FliC adjuvant effect [20, 21].

## Supplementary information

**Additional file 1: Schemes S1–S4** for the synthesis of PS–protein conjugates **6a**–**9b**. **Figure S1.** Structure of LPS and preparation of LFPS. **Figure S2.** Cytotoxicity test of LPS and LFPS. **Figure S3.** Fractionation of *S*. Typhimurium LFPS and IgG titer. **Figure S4.** Non-selective and site-selective modifications of flagellin (FliC). **Figure S5.** SDS-PAGE diagram of *S*. Typhimurium PS–FliC conjugates. **Figure S6.** FPLC diagrams of PS−protein conjugates **7a** and **7c**–**9b**. **Figure S7.** Endpoint titers of immune serum IgG specific to linkers and FliC. **Figure S8.** Endpoint titers of immune serum IgG induced by **7a** and **7b**.

## Data Availability

Supporting Information (PDF file): synthesis of PS–protein conjugates (Scheme S1–S4), Figures S1–S8, experimental details, and NMR spectra.

## Abbreviations

Abe: Abequose
APC: Antigen presenting cells
BCA: Bicinchoninic acid assay
BCR: B-cell receptor
BSA: Bovine serum albumin
CFU: Colony-forming unit
CRM197: Cross-reacting material 197
DT: Diphtheria toxoid
ELISA: Enzyme-linked immunosorbent assay
FliC: Flagellin
FPLC: Fast performance liquid chromatography
GlcN: Glucosamine
IgG: Immunoglobulin G
K3G5: KGKGKGGG octapeptide
Kdo: 3-deoxy-D-manno-octulosonic acid
KLH: Keyhole limpet hemocyanin
LFPS: Lipid-A free polysaccharide
LPS: Lipopolysaccharide
MEP: Mucoid exopolysaccharide
OMPC: Meningococcal outer-membrane protein complex
OVA: Ovalbumin
PS: Polysaccharide
PSA: Phenol−sulfuric acid assay
QXO: Quinoxalinone
RI: Refractive index
SDS-PAGE: Sodium dodecyl sulfate polyacrylamide gel electrophoresis
SEAP: Secreted embryonic alkaline phosphatase reporter assay
SEC: Size-exclusion chromatography
TLR: Toll-like receptor
TT: Tetanus toxoid
