# Supplementary material for: Constructing conjugate vaccine against Salmonella Typhimurium using lipid-A free lipopolysaccharide
Source: J Biomed Sci. 2020 Aug 24;27:89. doi: 10.1186/s12929-020-00681-8 (PMC7443816; doi:10.1186/s12929-020-00681-8)
Supplement: Supplementary file 1 — Additional file 1: Schemes S1–S4 for the synthesis of PS–protein conjugates 6a–9b. Figure S1. Structure of LPS and preparation of LFPS. Figure S2. Cytotoxicity test of LPS and LFPS. Figure S3. Fractionation of S. Typhimurium LFPS and IgG titer. Figure S4. Non-selective and site-selective modifications of flagellin (FliC). Figure S5. SDS-PAGE diagram of S. Typhimurium PS–FliC conjugates. Figure S6. FPLC diagrams of PS−protein conjugates 7a and 7c–9b. Figure S7. Endpoint titers of immune serum IgG specific to linkers and FliC. Figure S8. Endpoint titers of immune serum IgG induced by 7a and 7b. [file 12929_2020_681_MOESM1_ESM.pdf]

Supporting Information

Constructing Conjugate Vaccine against *Salmonella* Typhimurium Using Lipid-A Free Lipopolysaccharide

Tzu-Wei Chiu[a,1], Chi-Jiun Peng[a,1], Ming-Cheng Chen[a,1], Mei-Hua Hsu[b], Yi-Hua Liang[b], Cheng-Hsun Chiu[b,c,d,2], Jim-Min Fang[a,e,2], and Yuan Chuan Lee[f]

[a] Department of Chemistry, National Taiwan University, 1, Sec. 4, Roosevelt Rd., Taipei 10617, Taiwan.

[b] Molecular Infectious Disease Research Center, Chang Gung Memorial Hospital, 5, Fuxing St., Guishan Dist., Taoyuan 33302, Taiwan

[c] Department of Pediatrics, Chang Gung Children's Hospital, 5, Fuxing St., Guishan Dist., Taoyuan 33302, Taiwan

[d] Graduate Institute of Biomedical Sciences, College of Medicine, Chang Gung University, 259 Wenhua 1st Road, Guishan Dist., Taoyuan 33302, Taiwan

[e] The Genomics Research Center, Academia Sinica, 128, Sec. 2, Academia Rd., Taipei 11529, Taiwan

[f] Department of Biology, Johns Hopkins University, 3400 North Charles St., Baltimore, MD 21218-2685, U.S.A.

Corresponding Authors:

J.-M.F.: phone, 8862-33661663; E-mail, jmfang@ntu.edu.tw.

C.-H.C.: phone, 8863-3281200 ext. 8896; E-mail: chchiu@adm.cgmh.org.tw

**Contents**	**Page number**
Schemes 1–4 for the synthesis of PS–protein conjugates **6a**–**9b**	S2−S6
Figure S1. Structure of LPS and preparation of LFPS	S7
Figure S2. Cytotoxicity test of LPS and LFPS	S8
Figure S3. Fractionation of *S.* Typhimurium LFPS and IgG titer	S9
Figure S4. Non-selective and site-selective modifications of flagellin (FliC)	S10
Figure S5. SDS-PAGE diagram of *S.* Typhimurium PS–FliC conjugates	S11
Figure S6. FPLC diagrams of PS−protein conjugates **7a** and **7c**–**9b**	S12
Figure S7. Endpoint titers of immune serum IgG specific to linkers and FliC	S13
Figure S8. Endpoint titers of immune serum IgG induced by **7a** and **7b**	S14
Experimental section	S15–S32
NMR spectra	S33–S41

Synthesis of PS−protein conjugates 6a and 6b (Scheme S1)

Starting with 3,4-dinitrobenzoic acid, compound **13** bearing an *o*-phenylenediamine group was prepared and subjected to the condensation reaction with LFPS in aqueous AcOH buffer to afford the PS–QXO/NH_2 product **14**.[34,35] The condensation reaction was performed in the presence of 2-mercaptoethanol to prevent oxidation of *o*-phenylenediamine. Because the QXO moiety was UV and FL active, the reaction was easily monitored, and purification of the PS−QXO product (λ_{abs} = 346 nm and λ_{em} = 413 nm) was facilitated. PS–QXO/NH_2 **14** was treated with dithiobis(succinimidyl propionate) (**15**) for the amide bond formation, followed by addition of tris(hydroxymethyl)aminomethane to eliminate the residual reagent of succinimide ester. After the disulfide bond was reduced by tris(2-carboxyethyl)phosphine (TCEP), the free thiol group was revealed at the terminal of the linker. Excess reagents were removed via 3 kD dialysis membrane. After lyophilization, the PS−QXO/SH substance (**16**) was obtained in a solid form. Ellman's assay was employed to quantify the amount of the PS−QXO/SH substance. In this assay, the thiol group in the PS−QXO/SH sample was reacted with 5,5'-dithio-bis(2-nitrobenzoic acid) (DTNB) to cleave the disulfide bond, and the released 2-nitro-5-thiobenzoate ion (NTB^-) was further ionized to NTB^{2-} dianion at neutral or alkaline pH. The yellow NTB^{2-} ions were quantified by measuring the absorbance of 412 nm.

On the other hand, a carrier protein (BSA or OVA) was treated with *m*-maleimidobenzoic *N*-hydroxysuccinimide ester (MBS, **17**) in a cosolvent of DMSO and phosphate buffer (pH 7.3) for 1 h to obtain a derivative **18** having maleimide modification at the ε-amine groups of lysine residues. The MBS reagent could be replaced by sulfo-MBS, which contained a sulfonic acid substituent on the *N*-hydroxysuccinimide (OSu) moiety to increase water solubility. The size-exclusion chromatography on a Sephadex G-25 column was utilized to purify the maleimide-modified protein, which was characterized by the UV absorption at 280 nm and the Coomassie brilliant blue assay showing absorption at 595 nm. The number of modified lysine residues, ~30 in BSA and ~19 in OVA, was estimated by the MALDI-MS measurements. The conjugation of PS–QXO/SH (**16**) with the maleimide-modified BSA and OVA was then carried out through the Michael reaction to obtain the PS−protein conjugates **6a** and **6b**, respectively.

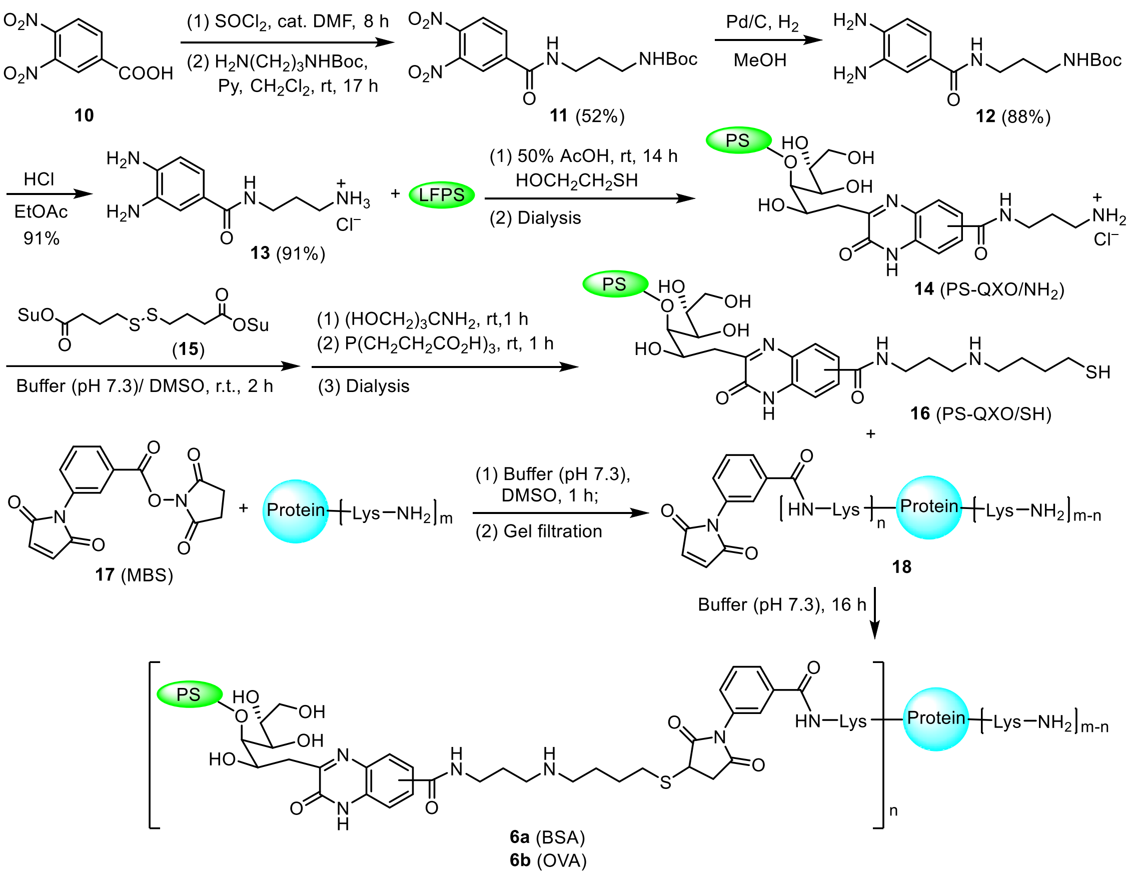

Scheme S1. Synthesis of PS−protein conjugates 6a and 6b. The condensation reaction of lipid-A free polysaccharide with an *o*-phenylenediamine derivative gives 14, which is subsequently elaborated to PS–QXO/SH (16) carrying a terminal thiol group for linkage with the maleimide modified proteins.

Synthesis of PS−protein conjugates 7a–7c (Scheme S2)

LFPS was treated with a DMSO solution containing iodine, Cs_2CO_3 and excess amount of linker A (**19**) bearing a terminal 2-nitrobenzenesulfonyl (Ns) group. The mixture was stirred at room temperature for 23 h, and $Na_2S_2O_3$ was added to eliminate the residual iodine. This procedure rendered conjugation of LFPS via an immune-compatible amide group.[34,37] The crude product was subsequently treated with sodium thiophenolate to remove the Ns protecting group, dialyzed and lyophilized to obtain PS−A/NH_2 (**21**). Although linker A/Ns could be replaced by mono-Boc derivative of 1,6-hexanediamine for the linkage with LFPS via a similar decarboxylative amidation reaction, the subsequent removal of the Boc protecting group in boiling water, or acidic media (e.g, TFA) caused degradation of PS in appreciable degree.

PS−A/NH_2 (**21**) reacted with excess amount of bis(4-nitrophenyl) adipate (**22**, i.e. linker B with two terminal Np groups) in DMF solution to afford the PS−A−B/Np product (**23**) after extraction with CH_2Cl_2 to remove the residual adipate reagent. The product **23** showed a characteristic absorption band of nitrophenoxy group at 387 nm.

The coupling reactions of PS−A−B/Np with BSA, OVA and FliC were performed in phosphate buffer (pH 7.4), respectively. The desired PS–A–B–BSA (**7a**), PS–A–B–OVA (**7b**) and PS–A–B–FliC (**7c**) conjugates were isolated by FPLC size exclusive chromatography. The efficiency of coupling reaction was appreciably increased by adding 10% of DMF as a cosolvent to improve the solubility of the linker-hinged PS.

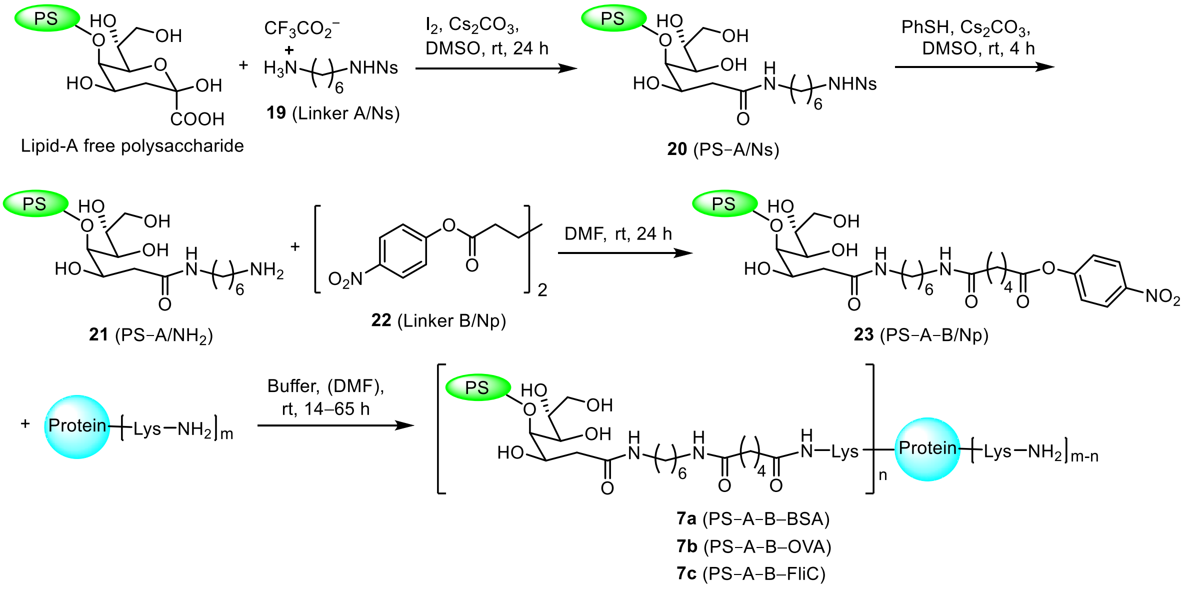

Scheme S2. Synthesis of PS–A–B–protein conjugates **7a**–**7c**. Decarboxylative amidation of lipid-A free polysaccharide with amine linker A (**19**) gives **20**, which carries a terminal 2-nitrobenzenesulfonyl (Ns) group. After removal of the Ns group, the tandem coupling reactions with linker B and the lysine residues of protein produce the conjugates **7a**–**7c**.

Synthesis of PS−protein conjugates 8a and 8b (Scheme S3)

Traceless Staudinger ligation is a biorthogonal conjugation method that enables the reaction of phosphinothioester and azide to form an amide bond.[41] This method was applied to prepare PS−A−B−C−BSA (**8a**) and PS−A−B−C−FliC (**8b**) conjugates. *S*-(Diphenylphosphino)methanethiol borane complex (**26**) was freshly prepared and reacted with the Boc-protecting 6-aminohexanoic acid (**25**) to give *S*-(diphenylphosphino)methyl 6-aminohexanethioate (**28**) after removal of the Boc protecting group. The coupling reaction of amine **28** with PS−A−B/Np (**23**) was readily carried out to afford PS−A−B−C/PPh_2 (**29**). The traceless Staudinger ligation of **29** with the azido-modified BSA (**30a**) and FliC (**30b**) thus produced the desired conjugates **8a** and **8b**.

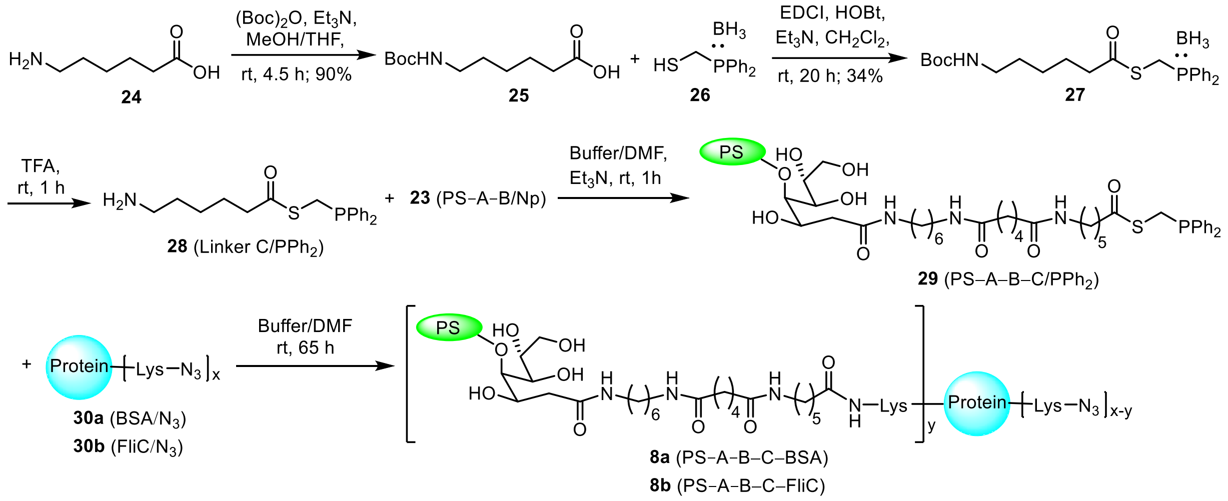

Scheme S3. Synthesis of PS−A−B−C−protein conjugates **8a** and **8b**. The linkers A, B and C are derived from 1,6-hexadiamine, 1,6-hexanedioic acid and 6-aminohexanoic acid, respectively. Traceless Staudinger ligation is in amide bond formation.

Synthesis of PS−protein conjugates 9a and 9b (Scheme S4)

A lysine-rich octapeptide KGKGKGGG (designated as K_3G_5 for convenience) was incorporated into the linker to assemble multiple PS immunogens for the synthesis of PS−A−B−K_3G_5−BSA (9a) and PS−A−B−K_3G_5−FliC (9b) conjugates. All the amino groups in K_3G_5 were protected with Boc groups, and then reacted with *S*-(diphenylphosphino)methanethiol (as a borane complex 26) to give a thioester. The K_3G_5/PPh_2 linker (32) was obtained by treatment with TFA to remove all the Boc and BH_3 protecting groups. The coupling reaction of K_3G_5/PPh_2 with PS−A−B/Np yielded a linker PS−A−B−K_3G_5/PPh_2 (33), which carried four units of polysaccharide immunogens. Ligation of 33 with azido-modified BSA and FliC provided the desired LFPS−protein conjugates 9a and 9b.

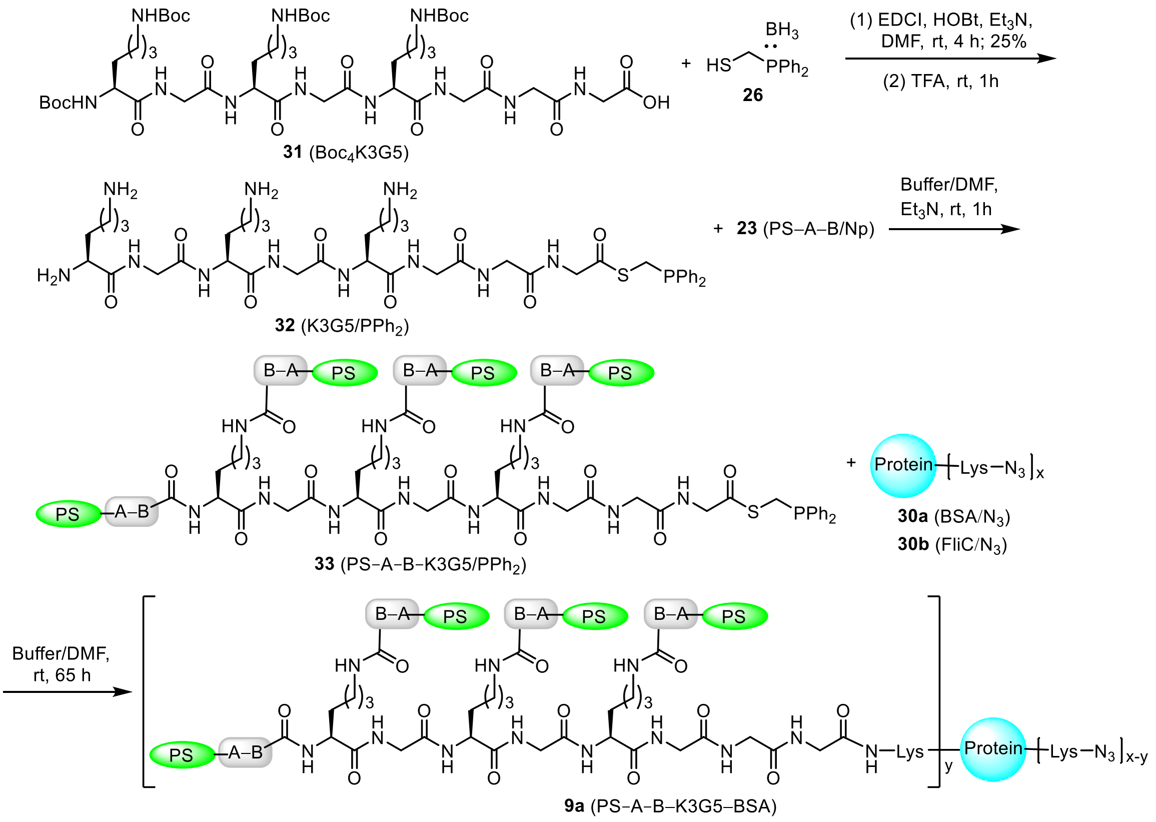

Scheme S4. Synthesis of PS−A−B−K_3G_5−BSA (9a) and PS−A−B−K_3G_5−FliC (9b) conjugates. The linkers A–B–K_3G_5 is derived from 1,6-hexadiamine, 1,6-hexanedioic acid and octapeptide KGKGKGGG for connection with multiple polysaccharide immunogens.

(A)

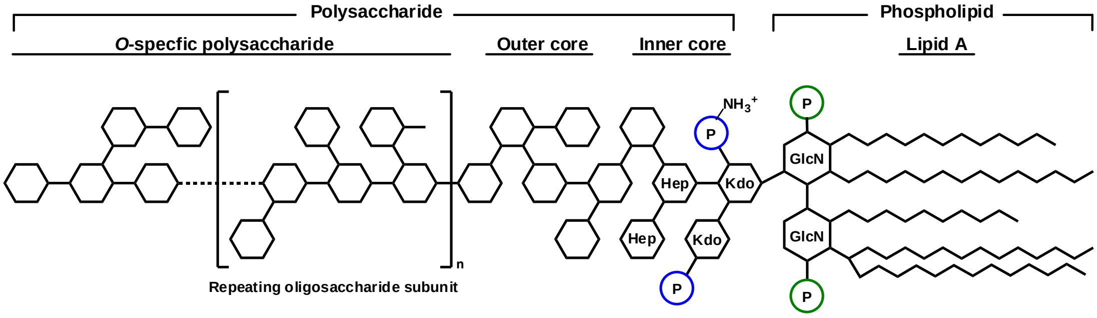

(B)

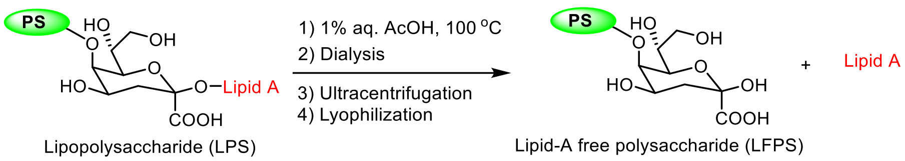

Figure S1. General structure of lipopolysaccharides (A) in Gram-negative bacteria and preparation of lipid-A free polysaccharide (B).

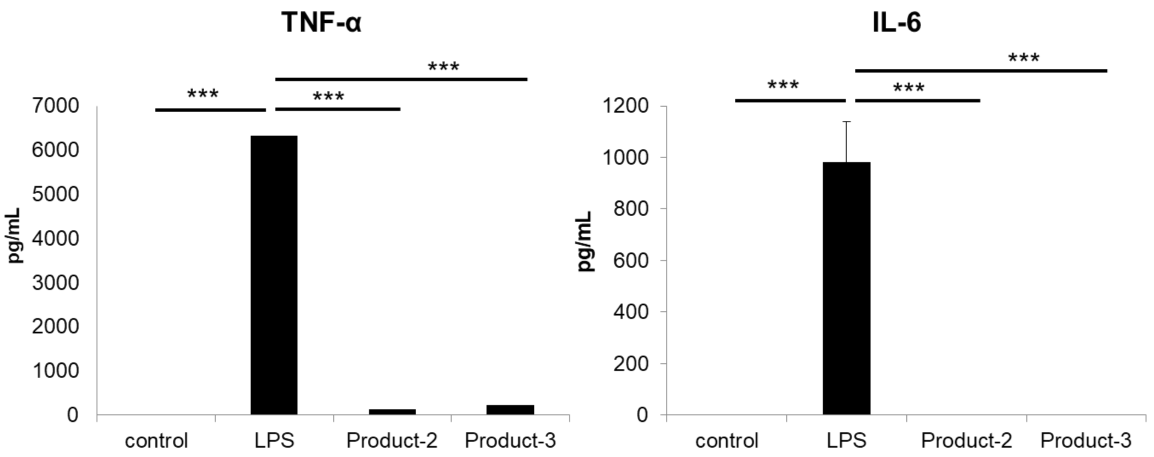

Figure S2. Cytotoxicity test of lipopolysaccharide (LPS) and the lipid-A free polysaccharide (LFPS) product prepared from the *S.* Typhimurium LPS provided by Chang Gung Memorial Hospital (Product-2) and from the purchased *E. coli* O111:B4 LPS (Product-3). PBS was used as control. *** $p < 0.001$.

Fractionation of *S*. Typhimurium LFPS.

The size-exclusion chromatography on a Superdex-200 pg column enabled fractionation of LFPS to give an overview analysis of the polysaccharides and protein contaminants. The *S*. Typhimurium LFPS (23 mg) was separated into three fractions in a period of 2 h by using 10 mM NaCl aqueous solution as eluent at a flow rate of 1 mL/min. Fraction-1 PS with high molecular weights (MW) was least retained in the Superdex column. The polysaccharide component of each fraction was monitored by refractive index (RI) detector, and the protein contaminant was detected by UV absorption at 280 nm. PSA assay was used to quantify the content of polysaccharides in each fraction. The total weight of the collected polysaccharides from three fractions was 12 mg (52% yield), comparable to the previously reported result.[28] Using pullulan as standard, the molecular weights of LFPS for fraction-1, 2 and 3 were estimated to be 85.8±31.8 kDa, 37.2±20.9 kDa and 10.4±5.8 kDa, respectively. The high-mass portion of LFPS induced higher IgG antibodies.

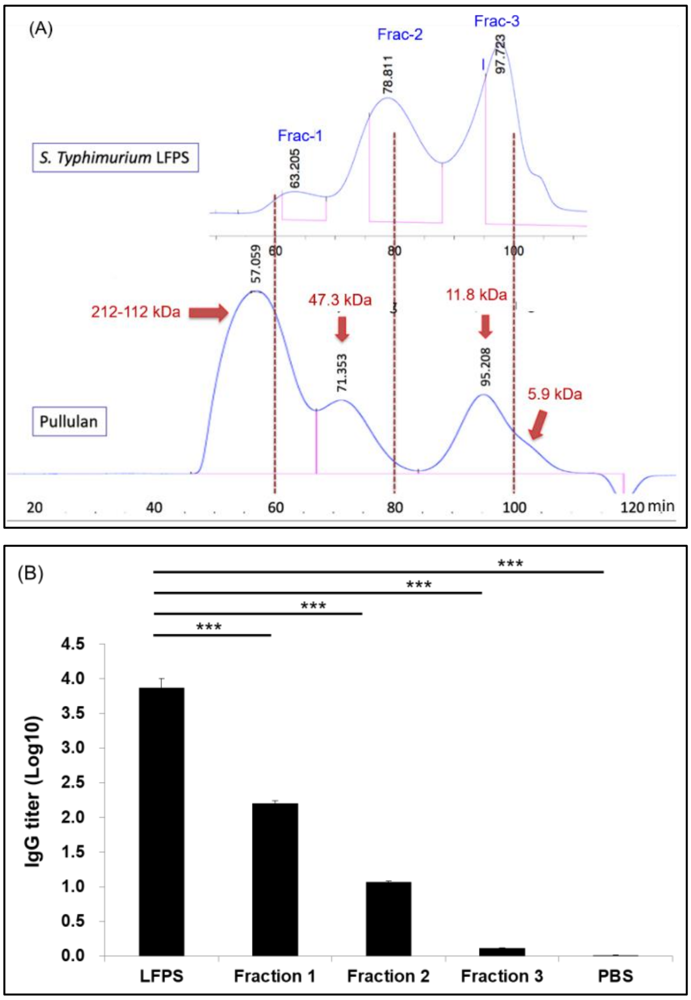

Figure S3. Fractionation of *S*. Typhimurium LFPS. (A) Size-exclusive chromatograph and determination of the molecular weights using pullulan standard. (B) IgG titer of each fraction. Column: Superdex-200. Eluent: 10 mM NaCl aqueous solution at a flow rate of 1 mL/min. *** $p < 0.001$.

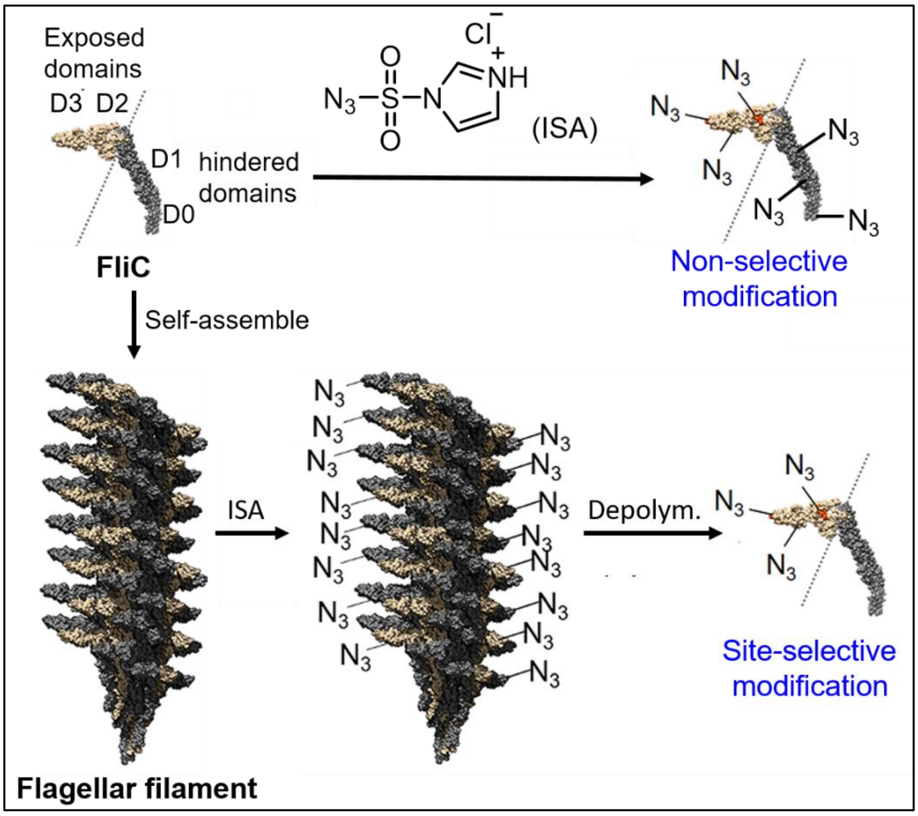

Figure S4. Non-selective and site-selective modifications of flagellin (FliC).[45, 46] Lysine residues in all the D0, D1, D2 and D3 domains in a FliC monomer can react with imidazole-1-sulfonyl azide. In high concentration of Na_2SO_4, FliC monomers undergo self-assembling to form flagellin filament. The chemical modifications mainly occur in the exposed D2 and D3 domains, while the D0 and D1 domains are compactly sheltered by each other inside the flagellar filament. The site-selective modified flagellin filament can be depolymerized in phosphate-buffered saline (pH 7) with brief heating at 65 ℃.

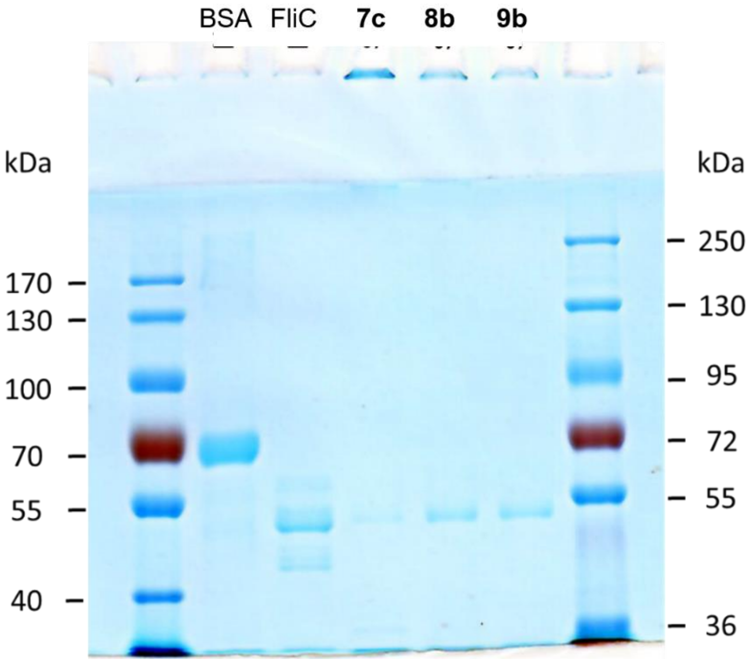

Figure S5. SDS-PAGE diagram of PS–FliC conjugates (**7c**, **8b** and **9b**) using 9% polyacrylamide gel and Coomassie blue staining. Most *S.* Typhimurium PS–FliC conjugates cannot enter the stacking gel due to their large molecular masses.

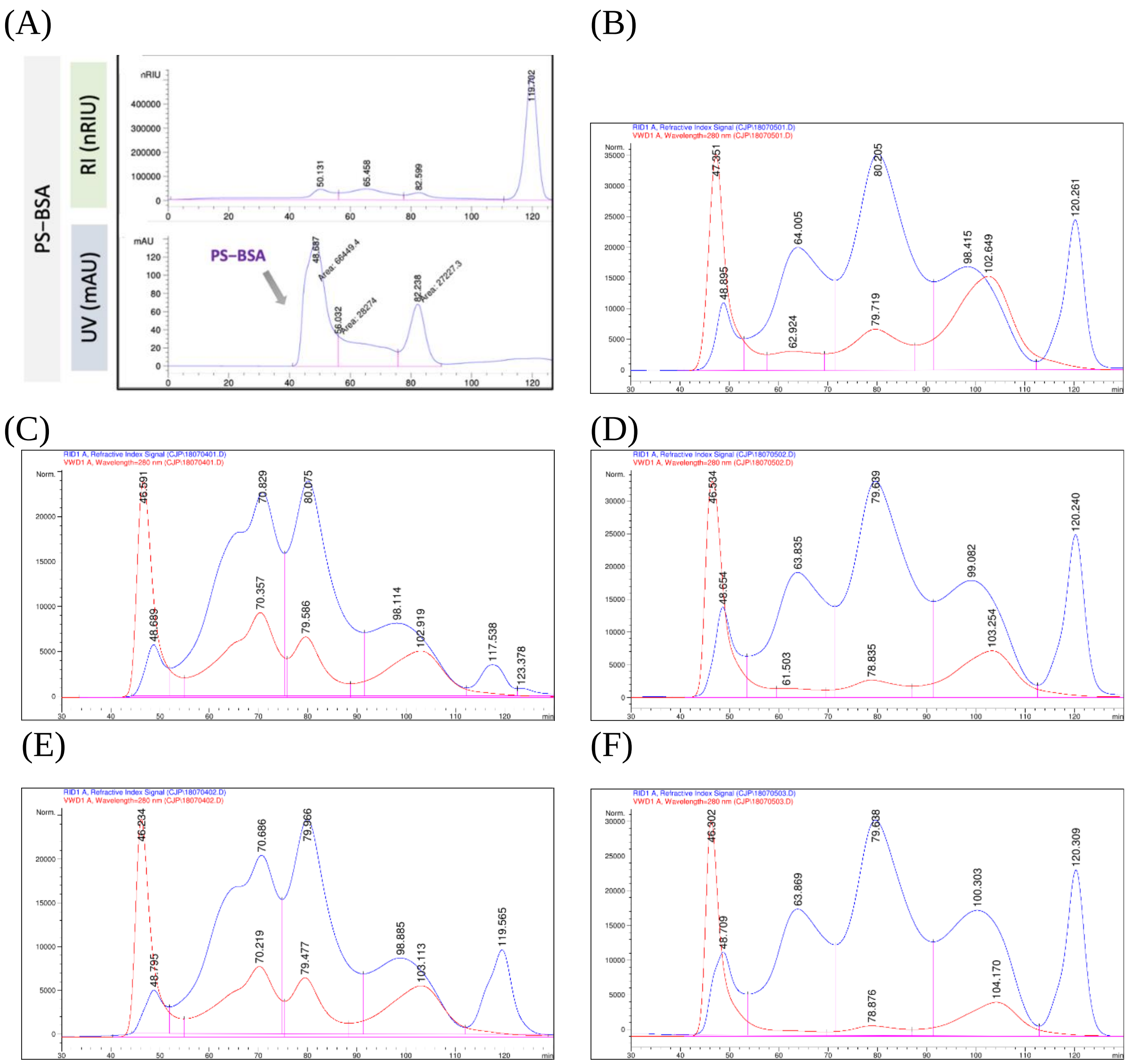

Figure S6. FPLC diagrams of PS−A–B–BSA **7a** (A), PS−A–B–FliC **7c** (B), PS−A–B–C–BSA **8a** (C), PS−A–B–C–FliC **8b** (D), PS−A–B–K3G5–BSA **9a** (E) and PS−A–B–K3G5–FliC **9b** (F). Conjugate **7a** is prepared from *S.* Typhimurium fraction-1 LFPS, whereas conjugates **7c**–**9b** are prepared from unfractionated LFPS. Column: Superdex-200 pg; eluent: PBS at a flow rate of 1 mL/min. For (A), the substance of signal at 120 min is not identified. For (B)–(C), RI signal (nRIU) and UV absorbance at 280 nm (mAU) are depicted in blue and red lines, respectively. The vertical scales of UV and RI signals are adjusted independently without correlation.

(A)

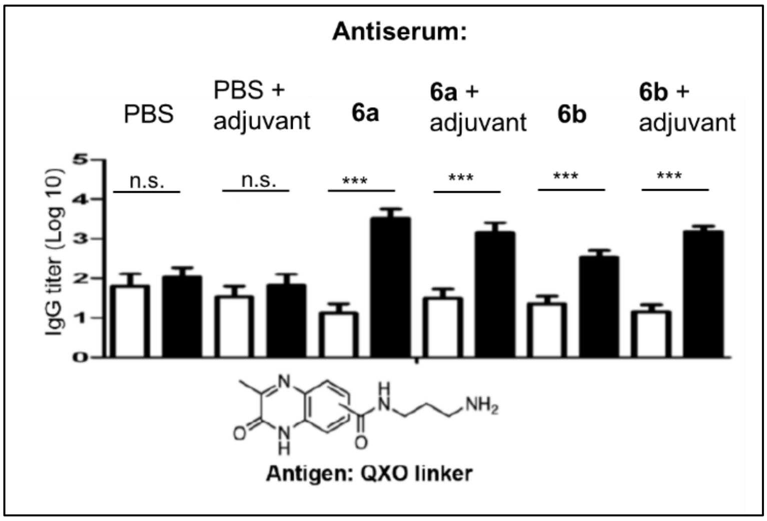

(B)

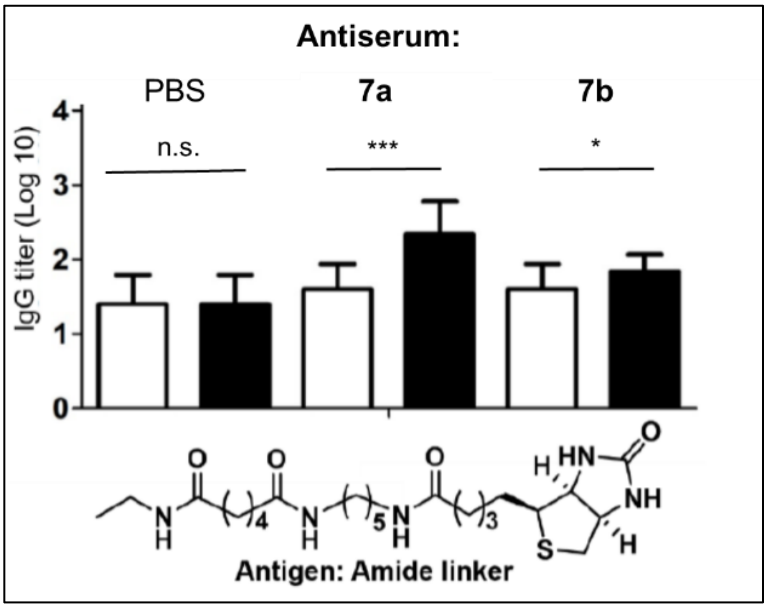

Figure S7. Endpoint titers of immune serum IgG specific to quinoxalinone (A) and amide linkers (B). The antigen is immobilized on a polystyrene microtiter plate. Blank bar is pre-immune serum, and filled bar is the post-immune serum with vaccination of the PS−protein conjugates **6a**–**7b**. * $p < 0.05$, *** $p < 0.001$.

(A)

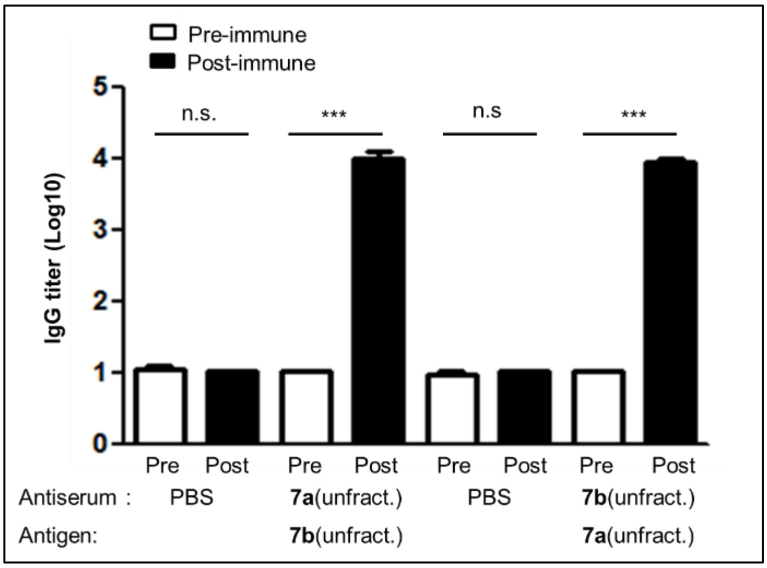

(B)

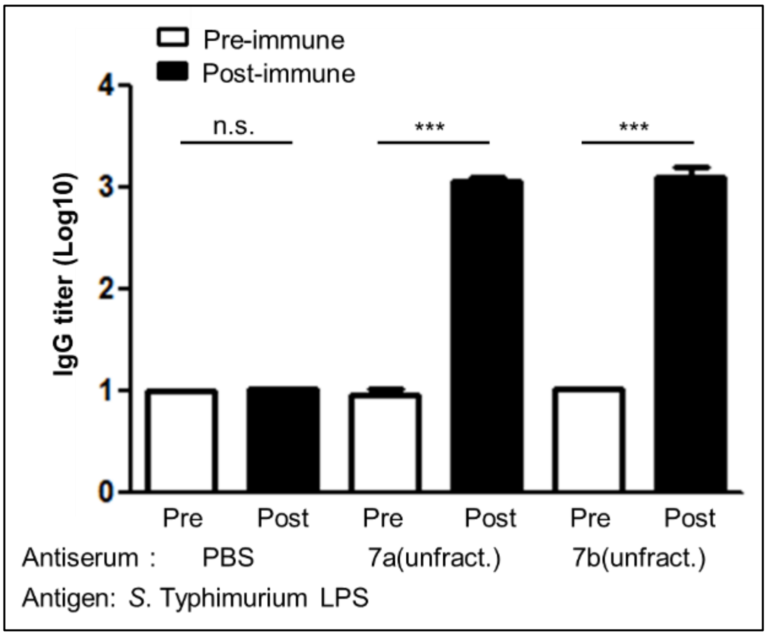

Figure S8. Endpoint titers of immune serum IgG induced by PS−A–B–BSA (**7a**) and PS−A–B–OVA (**7b**) conjugates, which were prepared from *S.* Typhimurium unfractionated LFPS, at a dosage of 2.5 μg. (A) The coating antigen was **7b** for PS–A−B−BSA anti-serum, and **7a** for PS–A−B−OVA anti-serum to minimize the crossed response of proteins. (B) specific to LPS. PBS was negative control. *** $p < 0.001$.

Experimental Section

Materials.

All the reagents and solvents were reagent grade and were used without further purification unless otherwise specified. CH_2Cl_2 was distilled from CaH_2. THF was distilled from sodium. MeOH was dried over CaH_2. Reactions were monitored by thin-layer chromatography on 0.25 mm E. Merck silica gel 60 F254 glass plates (Merck Millipore, Billerica, MA, USA; cat. 105554) using *p*-anisaldehyde, ninhydrin, cerium molybdate, phosphomolybdic acid, bromocresol green, potassium permanganate, or aqueous sulfuric acid as visualizing agents. E. Merck silica gel 60 (0.040–0.063 mm particle sizes), Fuji silysia silica gel MB 70–40/75 (0.040–0.075 mm particle sizes) and LiChroprep RP-18 (0.040–0.063 mm particle sizes) were used for column chromatography. Spetra/Pro Biotech cellulose ester membranes were used for dialysis. Disposable PD-10 desalting column was used for protein separation.

S. Typhimurium LPS (ATCC7823) was purchased from Sigma-Aldrich (St. Louis, MO, USA) or prepared in Chang Gung Memorial Hospital (CGMH), Taoyuan, Taiwan. *S.* Typhimurium FliC (SL1344 strain) was purchased from InvivoGen (San Diego, CA, USA) or prepared in CGMH. The HEK-Blue hTLR5 cells (cat. hkb-htlr5) and detection culture medium (cat. hb-det2) were also purchased from InvivoGen. Coomassie brilliant blue (CBB) G-250 was purchased from Biorad (Hercules, CA, USA). Bicinchoninic acid assay (BCA) kit for protein quantification was obtained from ThermoFisher (Waltham, MA, USA; cat. 23227). Lysine-rich Boc-protecting octapeptide Boc-K(Boc)GK(Boc)GK(Boc)GGG-OH was customized synthesis of Neogene (Taipei, Taiwan).

Instrumentation.

Melting points were recorded on a Yanaco micro apparatus and are uncorrected. Infrared (IR) spectra were recorded on a Thermo Nicolet iS5 FT-IR spectrometer (ThermoFisher). The UV absorbance for the BCA and PSA assays were recorded on a Synergy H1 Hybrid Multi-Mode Reader (BioTek, Winooski, VT, USA). Nuclear magnetic resonance (NMR) spectra were recorded on Varian Unity Plus-400 (400 MHz) or Bruker A VIII (500 MHz) NMR. Chemical shifts (δ) are given in parts per million (ppm) relative to residual solvent as internal standard: $CHCl_3$ (δ_H = 7.24), $CDCl_3$ (δ_C = 77.0 for the central line of triplet), CD_2HOD (δ_H = 3.31), CD_3OD (δ_C = 49.0), H_2O (δ_H = 4.81), $(CD_2H)_2CO$ (δ_H = 2.05), $(CD_3)_2CO$ (δ_C = 29.9), and $(CD_3)_2SO$ (δ_C = 39.5). Coupling constants (*J*) are given in hertz (Hz) and the splitting patterns are reported as s (singlet), d (doublet), t (triplet), q (quartet), m (multiplet), and br (broad). ESI–HRMS experiments were conducted on a Bruker Daltonics BioTOF III high-resolution mass spectrometer. High-performance liquid chromatography (HPLC) and fast protein liquid chromatography (FPLC) were both conducted on an Agilent 1100 series instrument equipped with Agilent G1314A ultraviolet detector and Agilent G1362A refractive index detector. Size

exclusion chromatography was conducted on HiLoad 16/600 Superdex 200 prep grade column (GE Healthcare, UK).

Isolation of *S.* Typhimurium lipopolysaccharide (LPS).

This experiment was conducted in Chang Gung Memorial Hospital. The bacterium *S.* Typhimurium (strain SL1344) was cultured in lysogeny broth at 37 °C for 16 h. The culture was added into fresh lysogeny broth with a dilution factor 1:100, and cultured at 37 °C for 5 h to reach an OD_{600} of 0.6−0.8. The LPS was isolated by a modified procedure according to Darveau−Hancock method.[27] Dried bacterial cells (1,200 mg) were suspended in tris(hydroxymethyl)aminomethane (Tris)-HCl buffer (60 mL, 10 mM, pH 8) supplemented with $MgCl_2$ (2 mM), DNase (100 μg/mL, DN25, Sigma) and RNase A (25 μg/mL, RNA888, Bioshop). The cell lysate was sonicated for two 30 s bursts at a probe intensity of 75. To ensure efficient nucleic acid digestion, DNase and RNase were added again to final concentrations of 200 and 50 μg/mL respectively. The suspension was then incubated at 37 °C for 2 h. After the incubation period, a solution of tetrasodium EDTA (5 mL, 0.5 M, ED4SS, Sigma) in Tris-HCl (10 mM, pH 8), a solution (5 mL) of 20% SDS in 10 mM Tris-HCl (pH 8) and 5 mL of Tris-HCl (10 mM, pH 8) were added to give a final volume of 50 mL for the sample containing 0.1 M EDTA, 2% SDS and 10 mM Tris-HCl at a pH about 9.5. The sample was vortexed to ensure solubilization of the components, and was then subjected to centrifugation at 50,000 *g* for 30 min at 20 °C to remove peptidoglycan. The supernatant was decanted, and pronase (P6911, Sigma) was added to give a final concentration of 250 μg/mL. After this, the sample was incubated for 16 h at 37 °C with constant shaking. After incubation in the presence of pronase, a precipitate sometimes developed. When this occurred, the precipitate was removed by centrifuging the sample in a clinical centrifuge at 1,000 rpm for 10 min (The supernatant was clear at this point). Two volumes of 0.375 M $MgCl_2$ in 95% ethanol were added, mixed and cooled to 0 °C by placing the flask in a dry ice bath (ethanol/water, 3:2) or in a –20 °C freezer. After the sample had cooled to 0 °C, it was centrifuged at 12,000 *g* for 15 min at 0 to 4 °C. The sample had to be kept as close to 0 °C as possible after the precipitate was formed and during the centrifugation. The pellet obtained was suspended in 35 mL of 2% sodium dodecylsulfate (SDS)–0.1 M tetrasodium ethylenediaminetetraacetic acid (EDTA), dissolved in 10 mM Tris-HCl (pH 8), and sonicated as described above (at this stage the solution was usually clear and the pH is about 9.5); however, the pH of the solution could be lowered to pH 7 by dropwise addition of 4 M HCl, to avoid slight saponification of lipids. The solution was then incubated at 85 °C for 30 min to ensure the denaturation of SDS-resistant proteins. After cooling, the pH was raised (if previously lowered) to 9.5 by addition of 4 M NaOH. Pronase (35 μg/mL) was added, and the sample was incubated for 16 h at 37 °C with constant shaking. After incubation, LPS was precipitated with 2 volumes of 0.375 M $MgCl_2$ in 95% ethanol at 0 °C as described above. The supernatant was followed by centrifugation at 12,000 *g* for 15 min at 0 to 4 °C. The

pellet after centrifugation was resuspended in 25 mL of 10 mM Tris-HCl (pH 8), sonicated as described above, and centrifuged in a clinical centrifuge at 1,000 rpm for 5 min to remove insoluble Mg^{2+}-EDTA crystals. The supernatant was then centrifuged at 100,000 *g* for 20 h at 15 °C in the presence of 25 mM $MgCl_2$. The pellet containing the LPS was resuspended in distilled water, and the DNA and protein contamination was checked by the UV absorbance at 260 nm and Coomassie brilliant blue assay.

Preparation of *S.* Typhimurium lipid-A free polysaccharide (LFPS).

S. Typhimurium LPS (200 mg) was dissolved in 1% AcOH (10 mL) and stirred at 100 °C for 2 h. The mixture was dialyzed with 3.5K membrane against H_2O four times at 1, 2, 4, and 16 h. After ultracentrifugation at 150,000 *g* for 5 h, the pellet of lipid A was removed, and the supernatant was purified by ultracentrifugation at 150,000 *g* for 17 h. The supernatant was lyophilized to get the desired LFPS. Alternatively, the dialyzed sample was subjected to ultracentrifugation once and purification by gel chromatography to give LFPS.

Non-selective azido modification of flagellin monomer and bovine serum albumin.

As a representative procedure, azido-modified FliC (FliC/N_3, **30b**) was prepared by adding an aqueous solution of imidazole-1-sulfonyl azide hydrochloride (60 mM, 5 μL) to a phosphate buffered saline (PBS solution, 60 μL, 50−167 mM) containing monomeric *S.* Typhimurium FliC (8−100 μg) and K_2CO_3 (125 mM, 8 μL). The mixture was gently shaken at room temperature for 1 h to give the FliC/N_3 having some lysine residues equipped with ε-azido groups. The azido-modified FliC were concentrated by spinning filtration (Amicon 10 kDa centrifugal filter, 7,000× *g*, 15 min) against 50 mM PBS (pH = 7) for two times, and the solution of FliC/N_3 monomers was stored at 4 °C until use. BSA was similarly modified to give BSA/N_3 (**30a**).

Site-selective azido modification of flagellin monomer.

To PBS (60 μL of 50−167 mM solution) containing 850 mM Na_2SO_4 and flagellar filaments (8−100 μg), the aqueous solutions of K_2CO_3 (125 mM, 8 μL) and imidazole-1-sulfonyl azide hydrochloride (60 mM, 5 μL) were successively added. The mixture was gently shaken at room temperature for 1 h to furnish the azido-modified flagellar filaments, which were concentrated by spinning filtration against 50 mM PBS (pH = 7) through Amicon 100 kDa centrifugal filter (14,000× *g*, 15 min) for two times to purify the site-selective modified flagellar filaments. The modified filaments in PBS solution were subjected to depolymerization by adding 1 M HCl until pH = 2 and gently shaking at room temperature for 30 min or simply by heating and gently shaking at 65 °C in PBS solution (pH = 7) for 30 min. The solution of site-selective modified FliC/N_3 monomers were concentrated by spinning filtration, and stored at 4 °C until use.

Phenol-sulfuric acid assay (PSA).

The content of carbohydrate was measured by phenol-sulfuric acid assay. A sample (25 μL) was pipetted into a well of 96-well plate, and freshly prepared phenol solution (30 μL 4% aqueous solution) was added. The mixture was gently shaken for 10 min, and concentrated H_2SO_4 (180 μL) was added rapidly. The mixture was shaken for another 5 min, and allowed to stand at room temperature for 25 min. A distinct change from colorless to yellow solution was observed. The UV absorbance at $\lambda = 490$ nm was measured with a plate reader, and the content of carbohydrate was quantified by interpolation to a standard line of mannose.

Bicinchoninic acid assay (BCA).

Bicinchoninic acid (BCA) assay was conducted following the procedure provided by manufacturer (Pierce Biotechnology Inc, Rockford, IL, USA) to quantify protein. A sample in sterilized H_2O (25 μL) was pipetted into a well of 96-well plate. A clear green solution of working reagent (200 μL) containing 50 parts of BCA Reagent A and 1 part of BCA Reagent B was added to each well. The mixture was shaken for 30 s, incubated at 37 °C for 30 min, and then cooled to room temperature. The absorbance of the produced BCA−Cu^+ purple-colored complex at 562 nm was measured, and the content of protein was quantified by interpolation to a standard curve of BSA.

Ellman's assay.

A mixture of KH_2PO_4 buffer (0.2 M, 5.3 mL), K_2HPO_4 buffer (0.2 M, 94.7 mL) and EDTA salt hydrate (76.0 mg) was diluted to 200 mL to obtain the reaction buffer. A sample of thiol-derived lipid-A free polysaccharide (25 μL) was pipetted into a well of 96-well plate. To which, 250 μL reaction buffer and 5 μL of diluted Ellman's reagent (containing 8 mg 5,5'-dithio-bis(2-nitrobenzoic acid (DTNB) in 1 mL reaction buffer) were added. The pH was adjusted to 8.0 with NaOH. After incubation for 15 min, the absorbance at 412 nm was measured, and the content of thiol was quantified by interpolation of a standard curve of 2-nitro-5-thiobenzoate dianion (TNB^{2-}).

Size-Exclusive Chromatography (SEC).

An Agilent 1100 high-pressure liquid chromatograph (HPLC) in our lab was modified in a way similar to fast performance liquid chromatograph (FPLC) for fractionation of LFPS. The stainless-steel pipelines in conventional HPLC system was changed into that of Teflon or polyether ether ketone (PEEK) material, which can sustain moderate pressure without deformation and also avoid adsorption of proteins. Size exclusion chromatography was conducted on an HiLoad 16/600 Superdex 200 prep grade column (GE Healthcare, Pittsburgh, USA). This instrumental system was equipped with RI and UV detectors.

In one experiment, *S.* Typhimurium LFPS sample (23 mg) was chromatographed on the Superdex-200 column by elution with 10 mM NaCl aqueous solution at a flow rate of 1 mL/min. LFPS was separated and collected as fraction-1 (0.9 mg), fraction-2 (5.5 mg) and fraction-3 (5.6 mg) after desalting and lyophilization. The total weight of recovered polysaccharide components was 12 mg (52% yield).

Fast performance liquid chromatography (FPLC).

An Agilent 1100 HPLC system was equipped with RI and UV detectors and a size-exclusion column (HiLoad 16/600 Superdex 200, GE Healthcare) for analysis and purification of LFPS-modified proteins. The eluent was 0.22 μm filtered 1× PBS (pH 7.4), and elution was performed at a flow rate of 1 mL/min (average backpressure 3-4 bar). The UV absorption of proteins was measured at $\lambda = 280$ nm. All chromatographic analyses were performed using the Chemstation software (v. A.10.02, Agilent, CA, USA).

LPS-induced human macrophage cell line assay.

THP-1 cells were treated with 100, 500 or 1,000 ng/mL of sample for 6 h. The secretion of TNF-α and IL-6 was determined by ELISA (R&D system) according to the manual. Briefly, ELISA plates were coated with capture antibody at room temperature for 14 h. The coated plates were washed 3 times with PBS containing 0.05% Tween 20 (PBST-20) and then blocked with 1% BSA in PBS at room temperature. After blocking for 1 h, plates were washed as above. Appropriate dilution of the cell supernatant was added to the well and incubated for 2 h at room temperature. After washing as above, detection antibody was added to the well for further 2 h incubation. Streptavidin–HRP was added to the well and incubated at room temperature for 20 min. Following the washing steps as described above, a TMB substrate solution was added to the well for 20 minutes and the reaction was stopped with 2 M H_2SO_4. The optical density at 450 nm of each well was measured.

Sodium dodecyl sulfate polyacrylamide gel electrophoresis (SDS-PAGE).

The LFPS–protein conjugates sample were subjected to SDS-PAGE to determine the molecular weights. The ladder was BR0671 from Biomate. The composition of stacking gel: 10% polyacrylamide (acrylamide/bisacrylamide = 29:1), 0.375 M Tris-HCl (pH 8.8), 0.1% SDS, 0.1% ammonium persulfate and 0.04% (*N*,*N*,*N′*,*N′*-tetramethyl)ethylenediamine (EDTA). The composition of running gel: 4% polyacrylamide (acrylamide/bisacrylamide = 29:1), 0.13 M Tris-HCl (pH 6.8), 0.1% SDS, 0.1% ammonium persulfate and 0.04% EDTA. Each sample (20 μL) was mixed with 5 μL of 5× diluted solution from SDS-PAGE sample buffer containing 300 mM Tris (pH 6.8), 12 mM EDTA, 12% SDS, 0.05% bromophenol blue, 60% glycerol and 864 mM β-mercaptoethanol. The mixed sample was incubated at 95 °C for 5 min, followed by cooling on ice for 20 min to denature protein. The electrophoresis was performed with SDS-

PAGE running buffer containing 25 mM Tris (pH 8.8), 250 mM glycine and 0.1% SDS at 70 volts for 20 min, followed by 100 volts for 1.5 h.

In another operation, SDS-PAGE was performed with standard procedures in a 1.5 mm, 9% polyacrylamide gel. Briefly, a protein sample was mixed with loading buffer (sample/loading buffer = 4:1 (v/v); GeneMark, cat. GM-47b). The sample was vortexed briefly and heated at 95−100 °C for 5−10 min. The sample (20 μL) was loaded into the well. The electrophoresis was run at 60 V through the stacking part of the gel and up to 150 V after the proteins have migrated into the resolving gel until the dye front reached the bottom of gel. The gel was rinsed with distilled water (dH_2O) to remove the running buffer, and then placed in fixing solution (isopropanol/dH_2O/acetic acid = 2.5:6.5:1 (v/v)) for 1−2 h with gentle shaking. After the fixing solution was poured off, the gel was rinsed with dH_2O, and Coomassie blue G-250 was applied for 1−2 h with gentle shaking. After the staining solution was removed, the gel was rinsed with dH_2O, and placed in a destaining solution (10% acetic acid) with gentle shaking until the clearly visible bands appeared (ca. 1−3 h).

Evaluation of hTLR5 activity.

The ability of FliC-based slef-adjuvanting vaccines to stimulate TLR5 was evaluated by a SEAP reporter cellular assay using an HEK-Blue hTLR5 cell line (InvivoGen). According to the protocol, a suspension of HEK-Blue hTLR5 cells was prepared at a concentration of 1.4×10^4 cells/mL in the HEK-Blue detection medium. Then, the cell suspension was added to a 96-well plate (180 μL/well, ≈ 25,000 cells per well). The native *S.* Typhimurium flagellin was used as a positive control, and 1× PBS buffer was used as a negative control. In the presence of different concentrations of the indicated FliC samples (50, 10 and 1 ng/mL), the cells were incubated at 37 °C for 24 h; then, TLR5 activation was evaluated by the absorbance at λ = 620 nm due to SEAP-catalyzed hydrolysis of the substrate. The data are presented as mean ± standard deviation (n = 5). The comparison of paired samples was performed by using Student's t-test.

Sandwich ELISA immunological assay.

The immunological assays were performed with general sandwich ELISA to measure the titer of linker-specific, hapten-specific, and carrier-specific antibodies. Sera were taken from immunized BALB/c mice by the retro-orbital bleeding method. ELISA plates (Corning, cat. 9018) were coated with distinct testing antigen at 4 °C for 14 h (5 μg LFPS, 2 μg linker–BSA conjugate, or 2 μg native FliC per well). The coated plates were blocked with 2% BSA at room temperature for 2 h. Plates were washed 3 times in PBS containing 0.05% Tween 20 (PBST-20). Then an appropriate diluted solution of mice serum was added to the well. Plates were incubated at room temperature for 2 h. After washing as described above, HRP-conjugated goat anti-mouse antibody (Millipore) was added to each well, and the plates were incubated at room temperature for 1 h. Following the washing steps as described above, a 3,3',5,5'-

tetramethylbenzidine (TMB) substrate solution was added to the well for 20 minutes, and the reaction was stopped with 2 M H_2SO_4. The optical density at 450 nm of each well was measured.

Mice immunization experiment.

The animal study was carried out in the laboratory animal center of Chang Gung University and compliance with the policy of animal care and use. Each group in the immunization experiments has 5 mice (BALB/c mice aged 6-8 weeks). BALB/c mice were immunized with the LFPS–protein conjugate (e.g. PS–A–B–BSA, **7a**) at a dose of 2.5 or 5 μg for four times on weeks 0, 2, 4 and 6. Sera were collected from immunized mice by eye-bleeding method (bleeding from the retroorbital venous plexus of mice) before immunization and after immunization on week 8. For the first immunization on week 0, the LFPS–protein conjugate was mixed with equal volume of Freund's complete adjuvant (Sigma). For the rest of immunization, the LFPS–protein conjugate (or LFPS) was mixed with equal volume of Freund's incomplete adjuvant (Sigma).

Alternatively, BALB/c mice aged 6-8 weeks were randomly assigned to one control group ($n = 5$) and three experimental groups of 10 mice. Mice were immunized with the LFPS–protein conjugate (e.g. PS–A–B–FliC, **7c**) at a dose of 2.5 μg by subcutaneous administration for three times on weeks 0, 2, and 4. No additive adjuvant was used for both initial vaccination and booster immunization. Sera were collected from immunized mice by retro-orbital bleeding method (bleeding from the retro-orbital venous plexus of mice) before immunization and after immunization on week 6.

Serum antibody titer test.

Anti-LFPS antibodies in BALB/c mice were determined by ELISA. Mouse sera were taken from immunized BALB/c mice by eye-bleeding method. ELISA plates were coated with 3 μg antigen (e.g. purified *S.* Typhimurium LFPS) at 4 ℃ for 16 h. The coated plates were blocked with 2% BSA at room temperature for 2 h. Plates were washed 3 times in PBS containing 0.05% Tween 20 (PBST-20). Then appropriate diluted solution of mouse serum was added to the well. Plates were incubated for 2 h at room temperature. After washing as described above, horseradish peroxidase (HRP) – conjugated goat anti-mouse antibody (Millipore) was added to each well and the plates were incubated at room temperature for 2 h. Following the washing steps as described above, a 3,3′,5,5′-tetramethylbenzidine (TMB) solution was added to the well for 20 min, and the reaction was stopped with 2 M H_2SO_4. The optical density at 450 nm of each well was measured.

Bacterial challenge test.

The immunized mice received oral or intravenous challenge with virulent pathogen (e.g. *S.* Typhimurium SL1344) equivalent to 10^6 CFU (oral challenge) and 500 CFU (intravenous

challenge) on day 14 after the last immunization. The mortality was recorded daily for 14 days.

Mice survival analysis.

The Kaplan-Meier method and the log-rank test were used to compare the survival of mice in the immunized and control groups. The survival times were also modeled using Cox proportional hazards regression to estimate the hazard ratios and protective factors of vaccination with distinct vaccines compared to the controls.[50] Data analysis was performed using IBM SPSS Statics (version 24).

Synthetic procedures and compound characterization

***Tert*-Butyl 3-(3,4-dinitrobenzamido)propylcarbamate (11).**

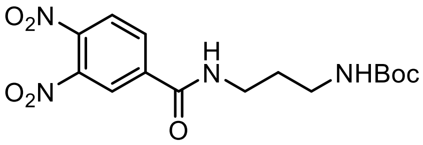

A mixture of 3,4-dinitrobenzoic acid (424 mg, 2.0 mmol), thionyl chloride (8 mL) and a small amount of DMF (2 drops) was heated under reflux for 8 h. The mixture was concentrated by rotary evaporation to remove excess $SOCl_2$. The residue was cooled in an ice bath, and anhydrous CH_2Cl_2 (8 mL) and pyridine (0.6 mL) were added, followed by dropwise addition of a solution of *N*-Boc-1,3-propylenediamine (523 mg, 3.0 mmol) in anhydrous CH_2Cl_2 (2 mL). The mixture was warmed to room temperature, stirred for 17 h, and partitioned between 1 M HCl (30 mL) and CH_2Cl_2 (30 mL). The organic phase was washed once with brine (40 mL), concentrated under reduced pressure, and purified by silica gel column chromatography (EtOAc/hexane, 1:1.5) to afford the desired product **11** (383 mg, 52% yield). $C_{15}H_{20}N_4O_7$; yellow solid, mp = 150.6–152.0 ℃; TLC (EtOAc/hexane, 1:1.5) R_f = 0.25; ^{1}H NMR ($CDCl_3$, 400 MHz) δ 8.52 (1 H, s), 8.38 (1 H, br), 8.28 (1 H, dd, *J* = 8.1, 1.4 Hz), 7.96 (1 H, d, *J* = 8.1 Hz), 4.90 (1 H, br), 3.50 (2 H, dd, *J* = 12.0, 6.2 Hz), 3.25 (2 H, dd, *J* = 12.0, 6.2 Hz), 1.74–1.68 (2 H, m), 1.43 (9 H, s). ^{13}C NMR ($CDCl_3$, 100 MHz) δ 162.5, 157.8, 144.0, 142.8, 139.6, 132.0, 125.3, 124.3, 80.4, 36.9, 36.2, 29.7, 28.4, 28.3 (2 ×). ESI–HRMS (negative mode) calcd for $C_{15}H_{19}N_4O_7$: 367.1254; found: *m/z* 367.1252 $[M - H]^-$.

***Tert*-Butyl 3-(3,4-diaminobenzamido)propylcarbamate (12).**

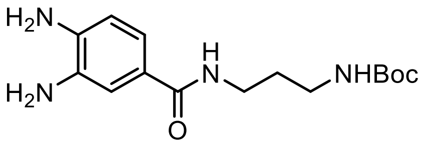

A mixture of compound **11** (250 mg, 0.68 mmol) and 10% Pd/C (25 mg) in MeOH (15 mL)

was stirred under an atmosphere of H_2 for 3 h at room temperature. The palladium catalyst was removed by filtration through Celite. The filtrate was concentrated under reduced pressure, and purified by silica gel column chromatography (MeOH/EtOAc, 3:97) to afford the desired product **12** (184 mg, 88% yield). $C_{15}H_{24}N_4O_3$; dark red foam; TLC (MeOH/EtOAc, 3:97) R_f = 0.18; ^{1}H NMR ($CDCl_3$, 400 MHz) δ 7.16 (1 H, s), 7.11 (1 H, d, J = 8.0 Hz), 6,57 (1 H, d, J = 8.0 Hz), 5.22 (1 H, br), 3.6 (2 H, br), 3.11 (2 H, br), 1.63–1.58 (2 H, m), 1.39 (9 H, s). ^{13}C NMR ($CDCl_3$, 100 MHz) δ 167.9, 156.8, 138.6, 133.8, 125.5, 119.3, 115.9, 115.1, 79.3, 37.1, 36.1, 30.2, 28.4 (3 ×). ESI–HRMS calcd for $C_{15}H_{25}N_4O_3$: 309.1923; found: m/z 309.1928 $[M + H]^+$.

***N*-(3-Aminopropyl) 3,4-diaminobenzamide (13, as the hydrochloric salt).**

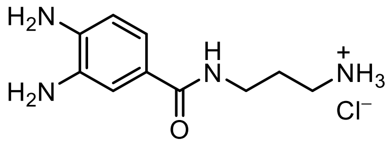

Compound **12** (180 mg, 0.58 mmol) was dissolved in EtOAc (2 mL), followed by dropwise addition of 3 M HCl (5 mL). The mixture was stirred at room temperature for 2 h, and then concentrated under reduced pressure. The crude product was purified by C18 reverse-phase silica gel column chromatography with elution of water, and then lyophilized to afford the desired produce **13** (130 mg, 91% yield). $C_{10}H_{17}ClN_4O$; white solid; mp = 199.6–202.1 ℃ TLC (10% MeOH in EtOAc) R_f = 0.1; ^{1}H NMR (D_2O, 400 MHz) δ 7.63 (1 H, s), 7.59 (1 H, d, J = 8.4 Hz), 7.07 (1 H, d, J = 8.4 Hz), 3.50 (2 H, t, J = 7.2 Hz), 3.09 (2 H, t, J = 7.2 Hz), 2.00 (2 H, m). ^{13}C NMR (D_2O, 100 MHz) δ 169.5, 140.6, 127.1, 124.9, 122.8, 119.6, 118.4, 37.1, 36.5, 26.8. ESI–HRMS calcd for $C_{10}H_{17}ClN_4O$: 209.1402; found: m/z 209.1403 $[M+H]^+$.

Conjugation of LFPS with *o*-phenylenediamine derivative (13), giving PS–QXO/NH_2 (14).

LFPS (45 mg) and *o*-phenylenediamine derivative **13** (40 mg, 0.16 mmol) were dissolved in aqueous AcOH buffer (4 mL, AcOH/ddH_2O = 1: 1, v/v), and 2-mercaptoethanol (23.07 μL, 0.32 mmol) was added. The mixture was stirred for 14 h at room temperature and then at 55 ℃ for 15 min under an atmosphere of argon. The mixture was dialyzed with 3.5 kDa membrane against ddH_2O four times at 1, 2, 4 and 14 h. The retentate was lyophilized to afford the desired PS–QXO/NH_2 product (**14**).

Bis(2,5-dioxopyrrolidin-1-yl) 4,4'-disulfanediyldibutyrate (15).

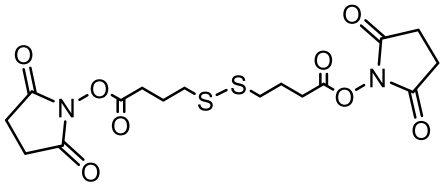

A mixture of 4,4'-dithiodibutyric acid (179 mg, 0.75 mmol), *N*-hydroxysuccinimide (190

mg, 1.65 mmol) and EDCI (315 mg, 1.65 mmol) was dried under vacuum for 1 h. The mixture was dissolved in anhydrous CH_2Cl_2 (4 mL), and stirred for 2 h at room temperature under an atmosphere of argon. The solution was then washed with saturated $NaHCO_3$ (3 mL × 2) and brine (3 mL). The organic phase was dried over $MgSO_4$, filtered and concentrated under reduced pressure. The crude product was purified by silica gel column chromatography (CH_2Cl_2/acetone, 30:1) to afford the desired product **15** (116 mg, 81% yield). $C_{16}H_{20}N_2O_8S_2$; white solid, mp = 126.4–127.8 ℃; TLC (CH_2Cl_2/acetone, 30:1) R_f = 0.25; ^{1}H NMR ($CDCl_3$, 400 MHz) δ 2.81 (8 H, s), 2.74 (8 H, m), 2.13 (4 H, m). ^{13}C NMR ($CDCl_3$, 100 MHz) δ 169.1, 167.9, 36.5, 29.2, 25.4, 23.7. ESI–HRMS calcd for $C_{16}H_{20}N_2O_8S_2Na$: 455.0559; found: *m/z* 455.0559 $[M + H]^+$.

Coupling reaction of PS–QXO/NH_2 with di-OSu compound 15, giving PS–QXO/SH (16).

PS–QXO/NH_2 **14** (65 mg) was dissolved in 50 mM phosphate buffer (pH 7.3, 4 mL), and a solution of di-OSu compound **15** (108.1 mg, 0.25 mmol) in DMSO (8 mL) was added. The mixture was stirred for 2 h at room temperature, and Tris base (84.8 mg, 0.7 mmol) in ddH_2O (0.6 mL) was added. The mixture was stirred for 1 h, then a solution of tris(2-carboxyethyl)phosphine (TCEP, 143 mg, 0.5 mmol) in ddH_2O (0.2 mL) was added. The mixture was stirred for 1 h, and dialyzed with 3.5 kDa membrane against ddH_2O four times at 1, 2, 4 and 14 h. The retentate was lyophilized to afford PS–QXO/SH product (**16**).

Modification of the lysine residues of protein by *m*-maleimidobenzoyl-*N*-hydroxysuccinimide ester, giving modified protein (18).

BSA (5.7 mg, 86 nmol, 30 lysine residues on its surface) was dissolved in 50 mM phosphate buffer (pH 7.3, 0.6 mL), and a solution of *m*-maleimidobenzoyl-*N*-hydroxysuccinimide ester (**17**, MBS) (3.2 mg, 10.32 μmol) in DMSO (0.4 mL) was added. The mixture was stirred for 1 h at room temperature, and then separated on a PD-10 desalting column using ddH_2O as eluent. Fractions of 0.5 mL per Eppendorf were collected. UV absorption at 280 nm and Coomassie brilliant blue assay were applied to determine the fraction that contained maleimide-activated BSA. The appropriate fractions were then lyophilized to afford the desired product of maleimide-activated BSA. The maleimide-activated OVA was similarly prepared.

Conjugation of PS–QXO/SH (16) with maleimide-activated carrier protein (18), giving PS–protein conjugates (6a and 6b).

A mixture of thiol-terminated LFPS–QXO/SH substance (**16**, 21.5 mg) and tris(2-carboxyethyl)phosphine (TCEP, 0.1 equiv to LFPS) in 50 mM phosphate buffer (pH 7.3, 2 mL) was stirred for 20 min at room temperature. A solution of maleimide-activated carrier protein (BSA or OVA, 9 mg) in 50 mM phosphate buffer (pH 7.3, 2 mL) was added. The mixture was

stirred for 14 h, and dialyzed with 3.5 kDa membrane against ddH_2O four times at 1, 2, 4 and 14 h. The retentate was lyophilized to afford the desired product of LFPS–protein conjugate **6a** (or **6b**).

***N*-(6-Aminohexyl) 2-nitrobenzenesulfonamide (19, linker A/Ns as the trifluoroacetate salt).**

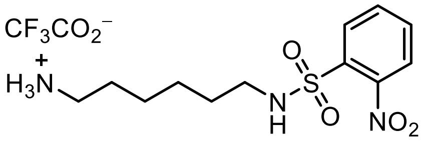

A solution of *N*-*tert*-butoxycarbonyl-1,6-hexanediamine (100 mg, 0.49 mmol) in dioxane/H_2O (6 mL, 1:1 (v/v)) was cooled and stirred in an ice bath. Et_3N (140 μL) was added, followed by dropwise addition of a solution of 2-nitrobenzenesulfonyl chloride (166 mg, 0.75 mmol) in dioxane (3 mL). The mixture was allowed to warm to room temperature, and stirred for additional 1 h. Most of dioxane was removed under reduced pressure, and H_2O was added to a volume of 10 mL. The aqueous mixture was extracted with CH_2Cl_2 (10 mL, 3×). The organic phase was dried over Na_2SO_4, filtered and concentrated under reduced pressure. The residue was chromatographed on a silica gel column by elution with hexane (100 mL) and EtOAc/hexane (1:3) to give *tert*-butyl (6-((2-nitrophenyl)sulfonamido)hexyl)carbamate (136 mg, 70% yield). $C_{17}H_{27}N_3O_6S$; white solid, mp = 82–83 ℃. TLC (EtOAc/hexane, 1:1) R_f = 0.58; ^{1}H NMR (400 MHz, CD_3OD) δ 8.08–8.06 (1 H, m), 7.84–7.78 (3 H, m), 3.03 (2 H, t, J = 7.0 Hz,), 3.00–2.95 (2 H, m), 1.54–1.46 (3 H, m), 1.42 (9 H, s), 1.41–1.21 (5 H, m); ^{13}C NMR (100 MHz, CD_3OD) δ 158.6, 149.7, 135.0, 135.0, 133.6, 131.6, 125.9, 79.9, 44.4, 41.3, 41.2, 30.5, 28.9, 24.8.

The above-prepared Boc-protecting compound (114 mg, 0.29 mmol) was treated with trifluoroacetic acid (TFA, 0.5 mL) in CH_2Cl_2 (1 mL). The mixture was stirred at room temperature for 1 h under an atmosphere of argon. The mixture was concentrated under reduced pressure to give the amine product **19** (as the TFA salt, 141 mg, quantitative yield). $C_{12}H_{19}N_3O_4S(CF_3CO_2H)$; dark brown oil; ^{1}H NMR (400 MHz, CD_3OD) δ 8.08–8.05 (1 H, m), 7.86–7.79 (3 H, m), 3.06 (2 H, t, J = 6.8 Hz), 2.89 (2 H, t, J = 7.7 Hz), 1.68–1.54 (5 H, m), 1.46–1.39 (2 H, m), 1.33–1.16 (1 H, m); ^{13}C NMR (100 MHz, CD_3OD) δ 149.6 135.1, 134.8, 133.7, 131.5, 125.9, 44.0, 40.6, 30.3, 28.1, 28.1, 24.4.

Conjugation of LFPS with linker A/Ns (19) via decarboxylative amidation of Kdo, followed by removal of Ns group, giving PS–A/NH_2 (21).

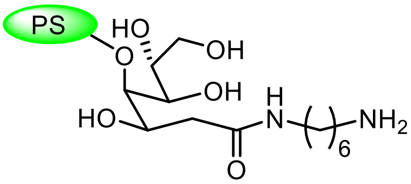

A suspension of *S.* Typhimurium LFPS (92.6 mg, 4.0 μmol, based on the average molecular weight of 23 kDa) in DMSO (2.5 mL) was sonicated at room temperature for 10 min to dissolve all solid particles. A solution of *N*-(6-aminohexyl)-2-nitrobenzenesulfonamide (**19**, linker A/Ns as the TFA salt, 14.4 mg, 35 μmol) in DMSO (0.5 mL) was added to a vial containing iodine (19.9 mg, 78.3 μmol) and Cs_2CO_3 (57.8 mg, 177 μmol). The mixture was added to the above-prepared DMSO solution of LFPS, and stirred at room temperature for 23 h under an atmosphere of argon. The reaction was quenched by addition of $Na_2S_2O_3$ (24.8 mg, 156.9 μmol) with stirring at room temperature for 10 min to give a crude product of PS–A/Ns (**20**).

Sodium thiophenolate (7.1 mg, 53.7 μmol) was added to the crude product **20**, and the mixture was stirred at room temperature for 3 h under an atmosphere of argon to remove the nosyl protecting group. The mixture was dialyzed with a mini-dialysis device (MWCO 3500 Da, Slide-A-Lyzer, ThermoFisher, MA, USA) against MeOH (30 min, 2×), MeOH/dH_2O = 1:1 (30 min, 2×), and then dH_2O (16 h). The retentate was lyophilized to give the PS–A/NH_2 product (**21**) as a yellow oil (122 mg).

Bis(4-nitrophenyl) adipate (22)

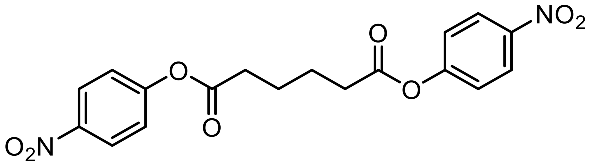

To a solution of adipoyl chloride (500 mg, 2.7 mmol) in anhydrous pyridine (2.7 mL) was added 4-nitrophenol (798 mg, 5.7 mmol) at 0 ℃. The mixture was stirred at room temperature for 15 min under an atmosphere of argon, and then concentrated under reduced pressure. The residue was quickly extracted with saturated $NaHCO_{3(aq.)}$ and EtOAc. The organic phase was dried over $MgSO_4$, and filtered. The filtrate was triturated with hexane, and the precipitate was collected to afford the desired product **22** (570 mg, 54% yield). $C_{18}H_{16}N_2O_8$; pale yellow solid; mp 123.5–124.5 ℃; TLC (hexane/EtOAc, 1:2) R_f = 0.70; IR ν_{max} (neat) 1750, 1594, 1517, 1489, 1347, 1249, 1215, 1162, 1149, 1122, 1107, 846, 852 cm^{-1}; ^1H NMR (400 MHz, $CDCl_3$) δ 8.27–8.23 (4 H, m), 7.28–7.24 (4 H, m), 2.69–2.66 (4 H, m), 1.90–1.86 (4 H, m); ^{13}C NMR (100 MHz, $CDCl_3$) δ 170.6 (2 ×), 155.3 (2 ×), 145.3 (2 ×), 125.2 (4 ×), 122.4 (4 ×), 33.8 (2 ×), 24.0 (2 ×); ESI−HRMS calcd for $C_{18}H_{17}N_2O_8$: 389.0979, found *m/z* 389.0998 $[M + H]^+$.

Conjugation of PS–A/NH_2 with bis(4-nitrophenyl) adipate, giving PS–A–B/Np (23).

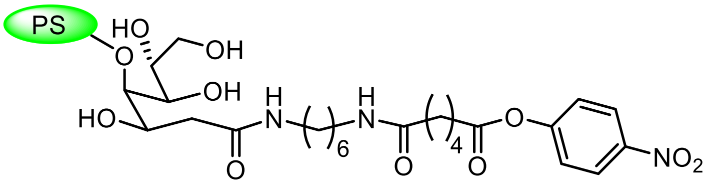

A suspension of *S.* Typhimurium PS–A/NH_2 (**21**, 113.6 mg) in DMF (5 mL) was sonicated at room temperature for 5 min to completely dissolve the solid particles. The PS–A/NH_2 solution was added dropwise (5 mL/15 min) to bis(4-nitrophenyl) adipate (34 mg, 87.5 μmol) in DMF (1 mL). The mixture was stirred at room temperature for 23 h under an atmosphere of argon, and then concentrated under reduced pressure to give a yellow oil. After successive washes with CH_2Cl_2 (3 mL), yellow solids were obtained. The solids were collected by centrifugation (5,000 *g*, 2 min), and then washed with CH_2Cl_2 (3 mL, 2×). After removal of CH_2Cl_2 under reduced pressure, the PS–A–B/Np product **23** was obtained as pale-yellow powder (96.2 mg).

Preparation of PS–A–B–BSA (7a) and PS–A–B–OVA (7b) conjugates.

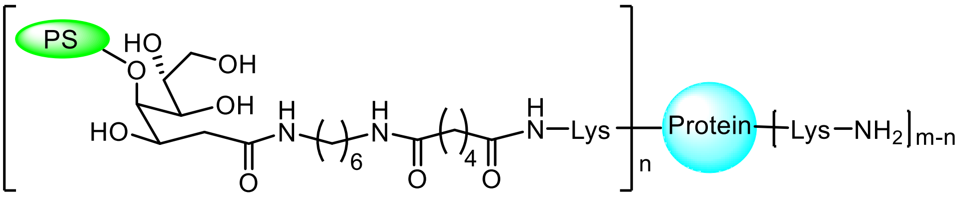

To a solution of BSA (1.4 mg) in phosphate buffer solution (PB, 0.5 mL, 400 mM, pH 7) was added *S.* Typhimurium PS–A–B/Np (**23**, 10.7 mg) in PB/DMF (2:1) solution (2 mL, 400 mM pH 8–9). The mixture was diluted by addition of PB solution (400 mM, pH 7) to a final volume of 7 mL. The mixture was gently shaken at room temperature for 65 h under an atmosphere of argon to furnish the PS–A–B–BSA conjugate (**7a**). The crude product was concentrated by spinning filtration against 1× PBS (pH 7) through an Amicon Ultra-15 10 kDa centrifugal filter (5,000 *g*, 30 min, 3×), sterilized through a 0.22 μm PES filter (Acrodisc Supor, Pall), and purified by FPLC (1× PBS) to give pure PS–A–B–BSA product (**7a**). The conjugate was assessed for the contents of carbohydrate and protein using PSA method and BCA assay, respectively.

PS–A–B–OVA (**7b**) and PS–A–B–FliC (**7c**) conjugates were prepared by the similar procedure.

6-((*Tert*-butoxycarbonyl)amino)hexanoic acid (25).

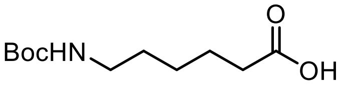

Boc anhydride (0.8 mL, 3.48 mmol) and Et_3N (20 μL) were added to a suspension of 6-aminohexanoic acid (300 mg, 2.29 mmol) in MeOH/THF (10 mL, 1:1 (v/v)). The mixture was stirred at room temperature for 4.5 h to obtain a clear solution. The mixture was concentrated under reduced pressure to give a yellow oil, which was subjected to silica gel column chromatography (MeOH/CH_2Cl_2, 1:9) to give the Boc derivative **25** (474 mg, 90% yield). $C_{11}H_{21}NO_4$; colorless oil; TLC (MeOH/CH_2Cl_2, 1:9) R_f = 0.55; ^1H NMR (400 MHz, $CDCl_3$) δ

3.08 (2 H, br d, J = 6.8 Hz), 2.31 (2 H, t, J = 7.4 Hz), 1.61 (2 H, quint, J = 7.4 Hz), 1.50−1.45 (2 H, m), 1.41 (9 H, s), 1.37−1.31 (2 H, m).

Diphenylphosphinomethanethiol borane complex (26).

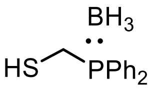

Thioacetic acid (1.5 mL, 21.3 mmol) was added to paraformaldehyde (750 mg), and the mixture was heated at 85 ℃ with stirring for 19 h. The mixture was cooled in an ice-bath under an atmosphere of nitrogen. Phosphorous tribromide (1.5 mL, 15.8 mmol) was added dropwise, and the mixture was kept below 5 ℃ with stirring for 10 min. The mixture was poured into ice water (15 mL), and extracted with Et_2O (15 mL). The ethereal layer was dried over anhydrous Na_2SO_4, and concentrated under reduced pressure. The crude product was purified by silica gel column chromatography by elution with hexane, followed by EtOAc/hexane (1:10), to give *S*-bromomethyl ethanethioate (748 mg, 21%). C_3H_5BrOS; yellow oil; TLC (EtOAc/hexane, 1:10) R_f = 0.48; ^{1}H NMR (400 MHz, $CDCl_3$) δ 4.69 (2 H, s), 2.38 (3 H, s).

NaH (60% dispersion in oil, 91.7 mg, 2.29 mmol) was placed in an oven-dried round-bottomed flask containing a stirring bar, and then sealed and connected to an argon balloon. Anhydrous DMF (1.2 mL) was added dropwise while the flask was cooled in an ice-bath. No bubbling was observed during addition of DMF. A solution of borane diphenylphosphine complex (458 mg, 2.29 mmol) in anhydrous DMF (0.6 mL) was added dropwise to the suspension of NaH in DMF with stirring until bubbling ceased. A solution of *S*-bromomethyl ethanethioate (374 mg, 2.21 mmol) in anhydrous DMF (0.6 mL) was then added to the mixture, which was cooled in an ice bath. The mixture was stirred for 10 min, allowed to warm to room temperature, and stirred for 24 h. EtOAc (5 mL) was added to the mixture, and the insoluble solids were filtered off using a cotton filter. The filtrate was collected, concentrated under reduced pressure, and subjected to silica gel column chromatography using a gradient of EtOAc/hexane (1:10, 100 mL; 1:4, 400 mL) to give diphenylphosphinomethyl thioacetate borane complex (247 mg, 39%). The product was stored at 4 ℃ until use. $C_{15}H_{18}BOPS$; colorless oil; TLC (EtOAc/hexane, 1:10) R_f = 0.20; (EtOAc/hexane, 1:4) R_f = 0.44; ^{1}H NMR (400 MHz, $CDCl_3$) δ 7.72–7.67 (4 H, m), 7.54–7.43 (6 H, m), 3.71 (2 H, d, J = 6.9 Hz), 2.25 (3 H, s), 1.45–0.55 (3 H, br m,); ^{13}C NMR (100 MHz, $CDCl_3$) δ 193.4 (d, J = 2.8 Hz), 132.6 (d, J = 9.5 Hz), 131.9 (d, J = 2.5 Hz), 129.0 (d, J = 10.2 Hz), 127.8 (d, J = 55.6 Hz), 23.9 (d, J = 35.5 Hz); ^{31}P NMR (162 MHz, $CDCl_3$) δ 20.0 (d, J = 77.6 Hz).

Diphenylphosphinomethyl thioacetate borane complex (110.6 mg, 0.38 mmol) was dissolved in anhydrous MeOH (1.6 mL), followed by addition of a suspension of sodium methoxide in anhydrous MeOH (30% (w/w), 100 µL). The mixture was stirred at room temperature for 10 min under an atmosphere of argon. An aqueous HCl solution (1 M) was

added to adjust the pH to 7, and the mixture was extracted with EtOAc against saturated aqueous NaCl (3×). The organic phase was collected, dried over $MgSO_4$, and then concentrated under reduced pressure to give the desired product **26** (101.5 mg, quantitative yield). This compound was not stable for long time storage at 4 ℃. $C_{13}H_{16}BPS$; colorless oil; TLC (EtOAc/hexane, 1:4) R_f = 0.48; ^{1}H NMR (400 MHz, $CDCl_3$) δ 7.73−7.68 (4 H, m), 7.53−7.43 (6 H, m), 3.19 (2 H, dd, J = 8.1, 6.1 Hz), 1.88 (1 H, m), 1.50−0.60 (3 H, br m); ^{13}C NMR (100 MHz, $CDCl_3$) δ 132.8 (d, J = 9.1 Hz), 132.0 (d, J = 2.4 Hz), 131.4 (d, J = 2.5 Hz), 129.2 (d, J = 9.9 Hz), 19.9 (d, J = 32.6 Hz); ^{31}P NMR (162 MHz, $CDCl_3$) δ 23.0 (d, J = 68.2 Hz).

S-((Diphenylphosphanyl)methyl) 6-((*tert*-butoxycarbonyl)amino)hexanethioate borane complex (27).

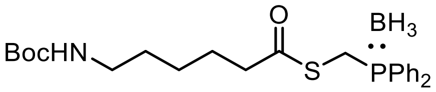

A solution of 6-((*tert*-butoxycarbonyl)amino)hexanoic acid (**25**, 35.6 mg, 0.15 mmol), HOBt•H_2O (49.5 mg, 0.32 mmol) and EDCI•HCl (129 mg, 0.66 mmol) in CH_2Cl_2 (3 mL) was stirred at room temperature for 10 min. A solution of freshly prepared diphenylphosphinomethanethiol borane complex (**26**, 46 mg, 0.19 mmol) in CH_2Cl_2 (3 mL) was added, followed by addition of Et_3N (74 μL). The mixture was stirred at room temperature for 20 h under an atmosphere of argon. The mixture was diluted with CH_2Cl_2 (7 mL), and extracted with saturated aqueous NaCl (3 × 10 mL). The organic phase was dried over anhydrous Na_2SO_4, and concentrated under reduced pressure. The crude product was purified by silica gel column chromatography (EtOAc/hexane, 1:4) to give the borane complex **27** (21 mg, 34%). $C_{24}H_{35}BNO_3PS$; colorless oil; TLC (MeOH/CH_2Cl_2, 1:9) R_f = 0.85; (EtOAc/hexane, 1:4) R_f = 0.25; ^{1}H NMR (400 MHz, $CDCl_3$) δ 7.85−7.80 (1 H, m), 7.70−7.64 (3 H, m), 7.51−7.40 (6 H, m), 3.69−3.64 (2 H, m), 3.08−3.00 (2 H, br m), 2.46−2.41 (2 H, m), 1.74−1.44 (7 H, m), 1.41 (9 H, s), 1.39−1.29 (4 H, m); ^{31}P NMR (162 MHz, $CDCl_3$) δ 20.3 (d, J = 72.4 Hz).

S-((Diphenylphosphanyl)methyl) 6-aminohexanethioate (28, linker C/PPh_2)

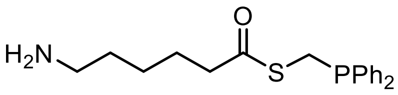

TFA (0.2 mL) was added to the brane complex **27** (2.1 mg, 4.6 μmol). The mixture was stirred at room temperature for 1 h under an atmosphere of argon to remove the Boc and borane protecting groups. TFA was removed under reduced pressure to give compound **28** as a pale-yellow oil. This product without further workup was redissolved in DMF (2 mL) and used for conjugation with PS–A–B/Np (**23**) in the next step.

Preparation of PS–A–B–C/PPh2 conjugate (29)

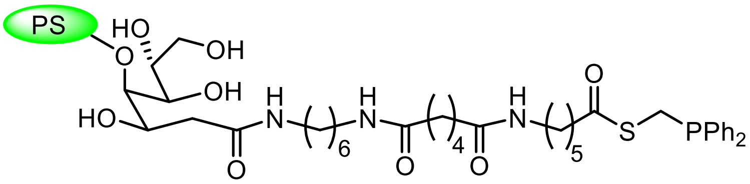

The freshly prepared linker C/PPh2 (28) from the borane complex (2.1 mg, 4.57 μmol) was dissolve in DMF (2 mL), and added to a solution of PS–A–B/Np **23** (32.1 mg, 1.4 μmol, based on the average molecular weight of 23 kDa) in PB (4 mL, 400 mM, pH 7). Triethylamine (40 μL, 0.29 mmol) was added, and the mixture was stirred at room temperature for 1 h. A solution of glycine (4.4 mg, 58.6 μmol) in PB (300 μL, 400 mM, pH 7) was added, and the mixture was stirred at room temperature for additional 10 min to consume the residual PS–A–B/Np (**23**). Without further purification, the solution containing PS–A–B–C/PPh2 (**29**) was used to prepare the PS–A–B–C–BSA (**8a**) and PS–A–B–C–FliC (**8b**) conjugates via traceless Staudinger ligation.

Preparation of PS–A–B–C–BSA (8a) and PS–A–B–C–FliC (8b) conjugates.

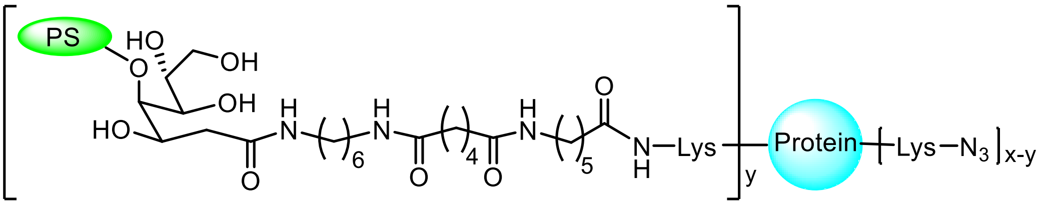

To a solution of N_3-modified BSA **30a** (1.4 mg) in degassed PB solution (0.5 mL, 400 mM, pH 7) was added *S.* Typhimurium PS–A–B–C/PPh2 (**29**) in a PB/DMF (2.2:1) solution (3.2 mL, 400 mM, pH 8–9). To the mixture was added degassed PB solution (400 mM, pH 7) to a final volume of 7 mL. The mixture was gently shaken at room temperature for 65 h under an atmosphere of argon to furnish the PS–A–B–C–BSA conjugate (**8a**). The crude product was concentrated by spinning filtration against 1× PBS (pH 7) through an Amicon Ultra-15 10 kDa centrifugal filter (5,000 *g*, 30 min, 3×), sterilized through a 0.22 μm PES filter (Acrodisc Supor, Pall), and purified by FPLC (1× PBS elution) to give pure PS–A–B–C–BSA conjugate (**8a**). By the similar procedure, PS–A–B–C–FliC conjugate (**8b**) was prepared. The conjugates were assessed for the contents of carbohydrate and protein using PSA method and BCA assay, respectively.

Boc-protected octapeptide Boc-K(Boc)GK(Boc)GK(Boc)GGG-OH (31, Boc4K3G5)

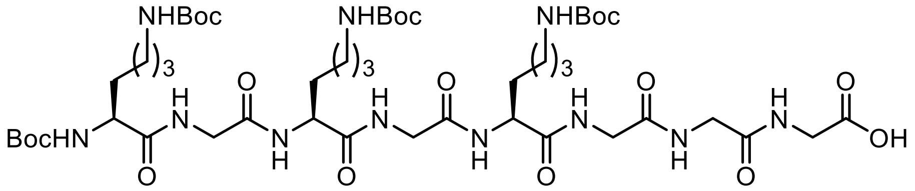

Et_3N (50 µL) was added to a solution of octapeptide H_2N-K(Boc)GK(Boc)GK(Boc)GGG-OH (105 mg, 0.11 mmol; Neogene, Taipei, Taiwan) in DMF (22 mL). The mixture was stirred in an ice bath for 10 min without precipitation. Boc anhydride (37 µL, 0.17 mmol) was added, and the mixture was stirred for another 30 min. The mixture was allowed to warm to room temperature, and stirred for 4 h. Most of DMF was removed under reduced pressure, and then cold Et_2O (20 mL) was added to the residue, followed by sonicating the mixture to form a suspension containing white particles. The suspension was allowed to stand at 4 ℃ for 16 h to provide white precipitates, which were collected by centrifugation (7,000 *g*) and washed with cold Et_2O (4×). The solvent was removed under reduced pressure to give the fully Boc protecting compound **31** (Boc_4K3G5, 76.3 mg, 66% yield). TLC ($MeOH/CH_2Cl_2$, 1:1) $R_f = 0.75$; ^1H NMR (400 MHz, CD_3OD) δ 4.28−4.22 (2 H, br m), 4.01−3.82 (11 H, m), 3.03 (6 H, m), 1.45−1.42 (36 H, m), 1.88−1.35 (18 H, br m).

Boc and borane protecting precursor of compound 32 (K3G5/PPh_2).

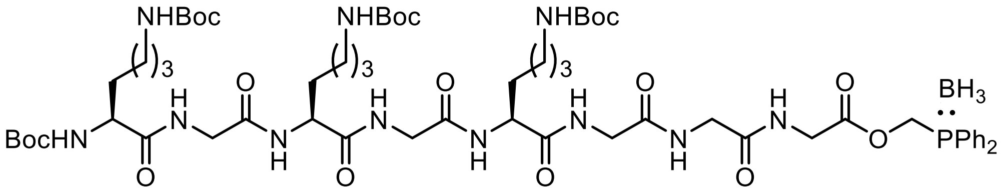

A solution of freshly prepared diphenylphosphinomethanethiol borane complex (**26**, 22.6 mg, 0.08 mmol) in EtOAc (5 mL) was added to Boc-K(Boc)GK(Boc)GK(Boc)GGG-OH (**31**, 44 mg, 0.04 mmol). The solvent was removed under reduced pressure. HOBt•H_2O (20.8 mg, 0.14 mmol) and EDCI•HCl (38.0 mg, 0.20 mmol) were added to the mixture, followed by addition of DMF (15 mL) and Et_3N (60 µL). The mixture was stirred at room temperature for 4 h under an atmosphere of argon. The mixture was concentrated by rotary evaporation under reduced pressure to give a crude product as colorless viscous oil, which was purified by silica gel column chromatography ($MeOH/CH_2Cl_2$, 1:9) to give the title compound (13 mg, 25% yield). TLC ($MeOH/CH_2Cl_2$, 1:9) $R_f = 0.57$; ^1H NMR (400 MHz, CD_3OD) δ 7.73−7.68 (4 H, m), 7.56−7.47 (6 H, m), 4.23 (2 H, br s), 4.04−3.81 (11 H, m), 3.73 (2 H, m), 3.01 (6 H, br s), 1.95−1.48 (18 H, br m), 1.45−1.43 (36 H, br m); ^{31}P NMR (162 MHz, $CDCl_3$) δ 20.2 (s).

For global removal of the Boc and borane protecting groups, the above-prepared complex (2.5 mg, 1.9 µmol) was stirred with trifluoroacetic acid (TFA, 0.2 mL) at room temperature for 1 h under an atmosphere of argon. After TFA was removed under reduced pressure,

K3G5/PPh_2 product (**32**) was obtained as a pale-yellow oil. Without further workup, the product was redissolved in DMF (2 mL) and used for conjugation with PS-A-B/Np (**23**) in the next step.

Preparation of PS–A–B–K3G5/PPh_2 (33)

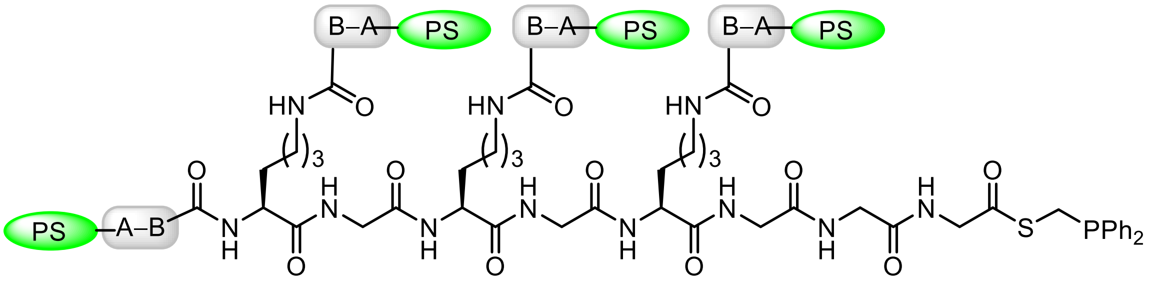

The K3G5/PPh_2 product (**32**) freshly prepared from the Boc and borane protecting precursor (2.5 mg, 1.9 μmol) was dissolved in DMF (2 mL). A solution of PS–A–B/Np (**23**, 32.1 mg, 1.4 μmol based on the average molecular weight of 23 kDa) in PB (4 mL, 400 mM, pH 7) and Et_3N (40 μL, 0.29 mmol) were added. The mixture was stirred at room temperature for 1 h. A solution of glycine (4.4 mg, 58.6 μmol) in PB (300 μL, 400 mM, pH 7) was added, and the mixture was stirred at room temperature for additional 10 min to consume the residual PS–A–B/Np (**23**). Without further purification, the solution containing PS–A–B–K3G5/PPh_2 (**33**) was used to prepare the PS–A–B–K3G5–BSA (**9a**) and PS–A–B–K3G5–FliC (**9b**) conjugates via traceless Staudinger ligation.

Preparation of PS–A–B–K3G5–BSA (9a) and PS–A–B–K3G5–FliC (9b) conjugates.

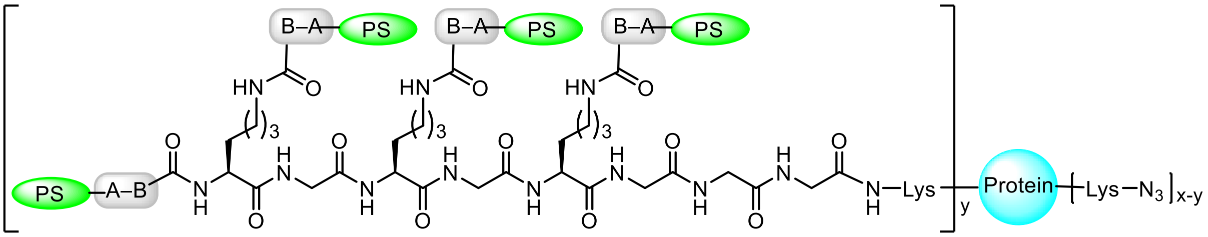

To a solution of N_3-modified BSA (1.4 mg) in degassed PB (0.5 mL, 400 mM, pH 7) was added a PB/DMF (2.2:1) solution (3.2 mL, 400 mM, pH 8–9) containing PS–A–B–K3G5/PPh_2 (**33**). To the mixture was added degassed PB (400 mM, pH 7) to a final volume of 7 mL. The mixture was gently shaken at room temperature for 65 h under an atmosphere of argon to furnish the PS–A–B–K3G5–BSA (**9a**) conjugate. The crude product was concentrated by spinning filtration against 1× PBS (pH 7) through an Amicon Ultra-15 10 kDa centrifugal filter (5,000 *g*, 30 min, 3×), sterilized through a 0.22 μm PES filter (Acrodisc Supor, Pall), and purified by FPLC (1× PBS elution) to give pure PS–A–B–K3G5–BSA (**9a**). By the similar procedure, PS–A–B–K3G5–FliC (**9b**) was prepared. The conjugates were assessed for the contents of carbohydrate and protein using PSA method and BCA assay, respectively.

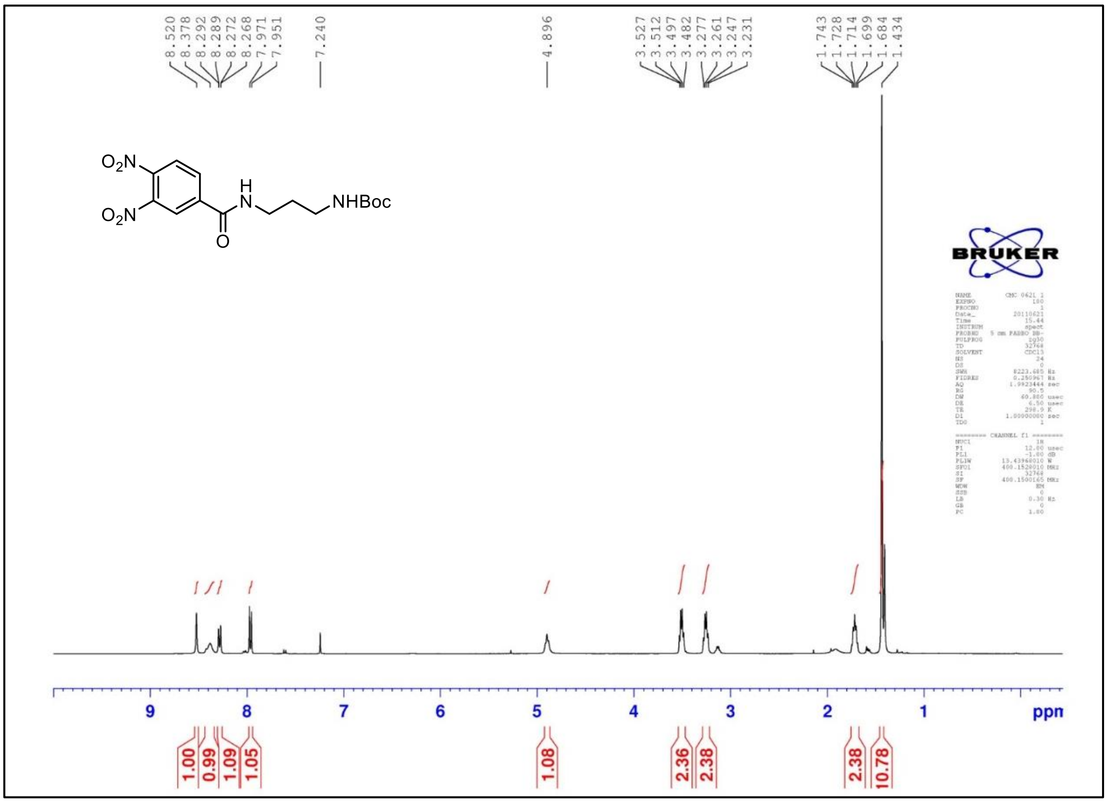

^{1}H NMR spectrum of compound **11** ($CDCl_3$, 400 MHz)

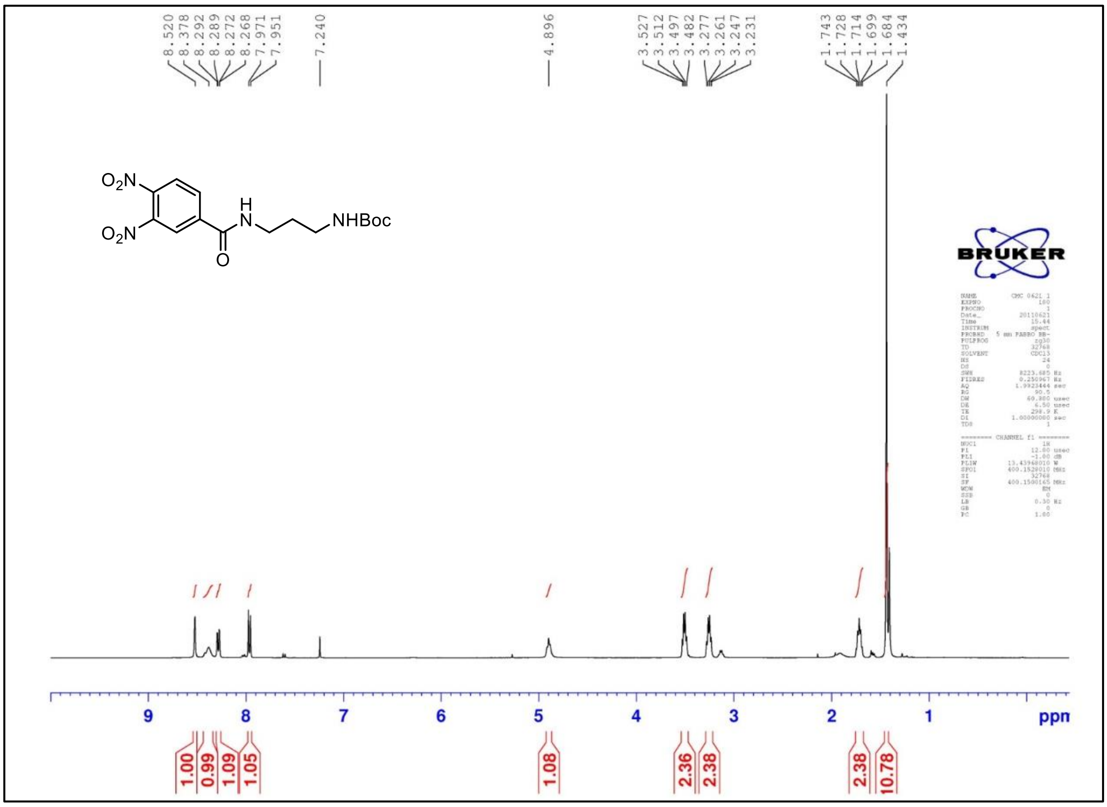

^{13}C NMR spectrum of compound **11** ($CDCl_3$, 100 MHz)

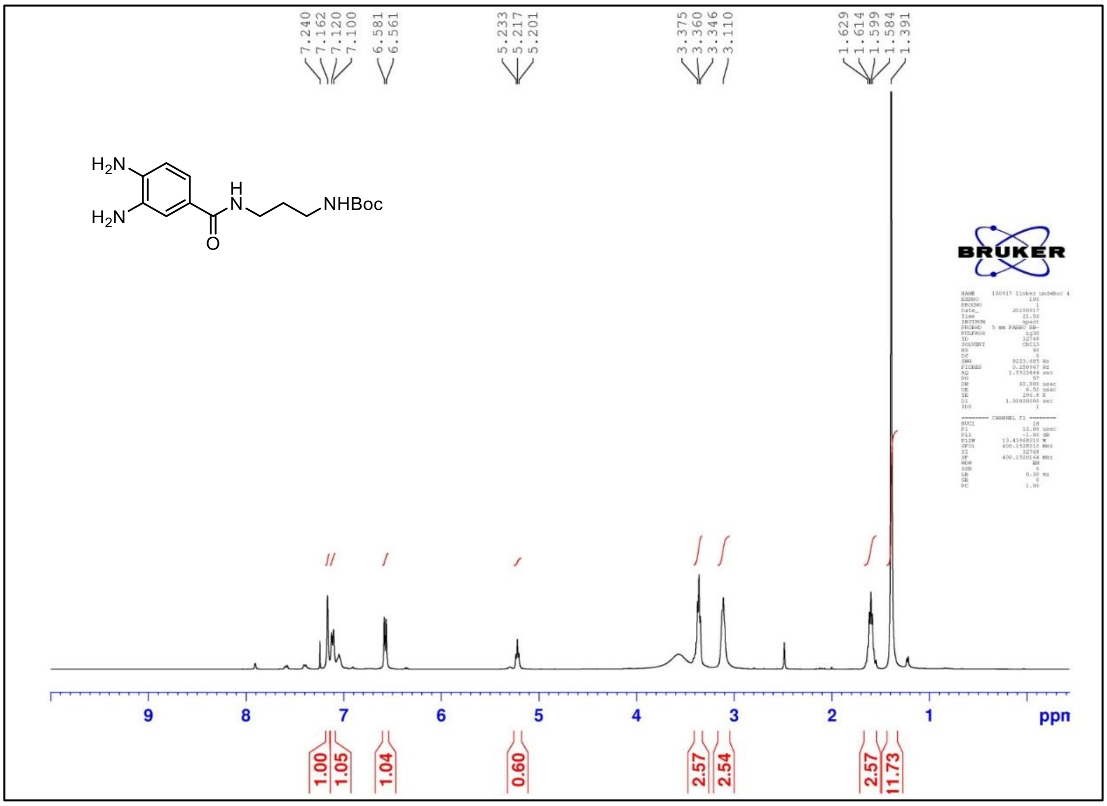

^{1}H NMR spectrum of compound **12** ($CDCl_3$, 400 MHz)

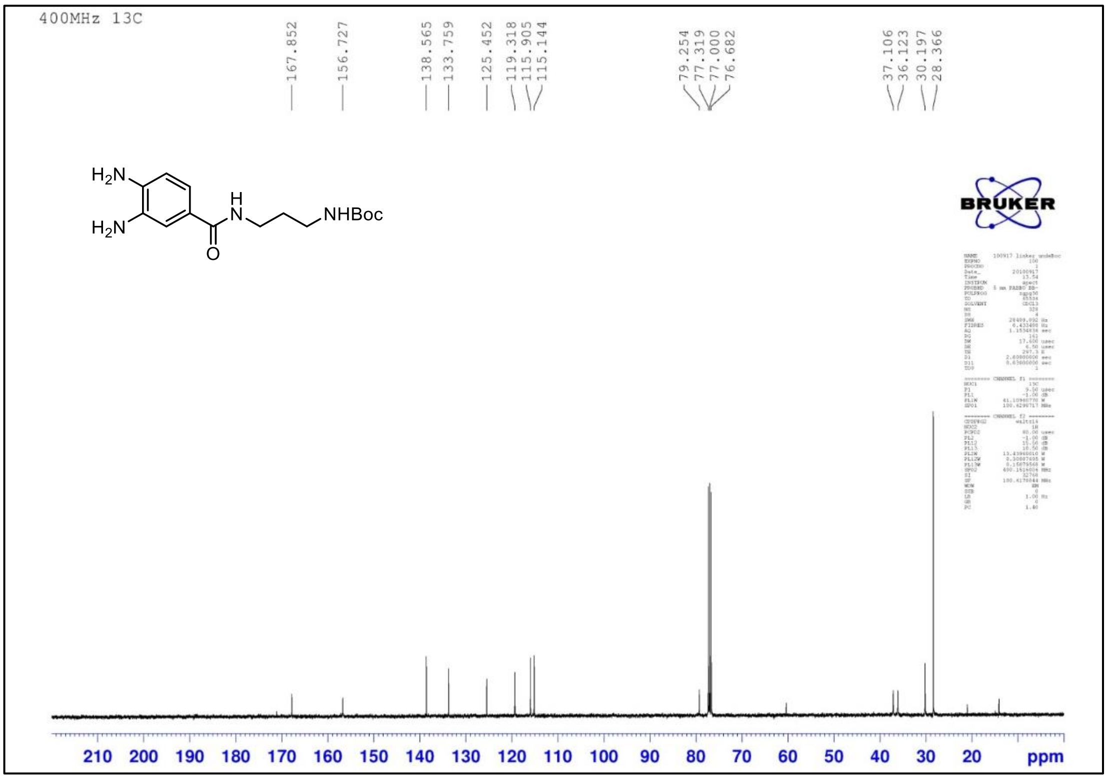

^{13}C NMR spectrum of compound **12** ($CDCl_3$, 100 MHz)

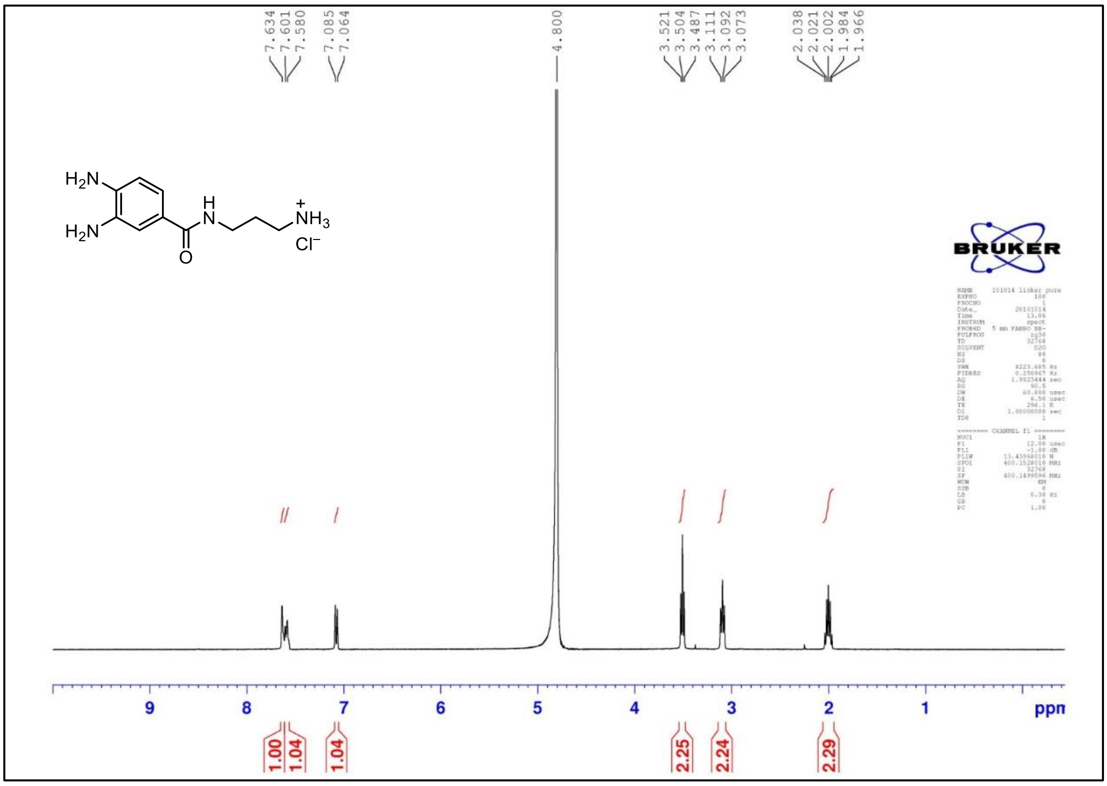

^{1}H NMR spectrum of compound **13** (D_2O, 400 MHz)

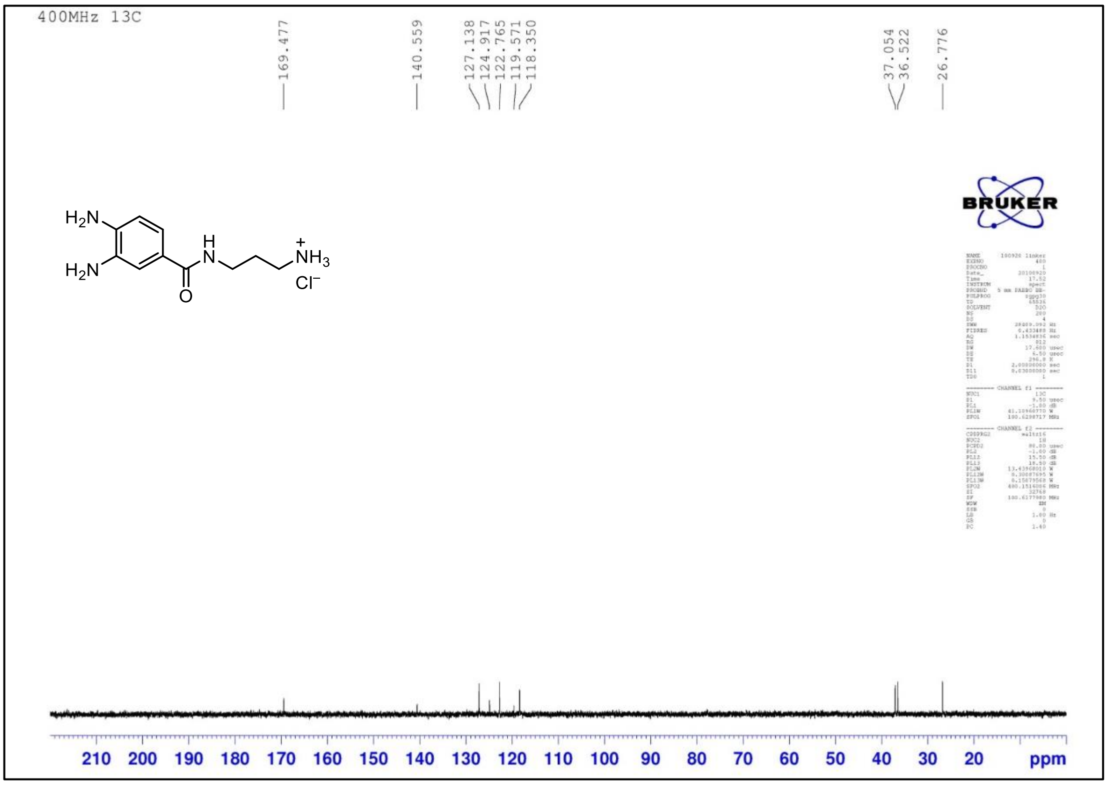

^{13}C NMR spectrum of compound **13** (D_2O, 100 MHz)

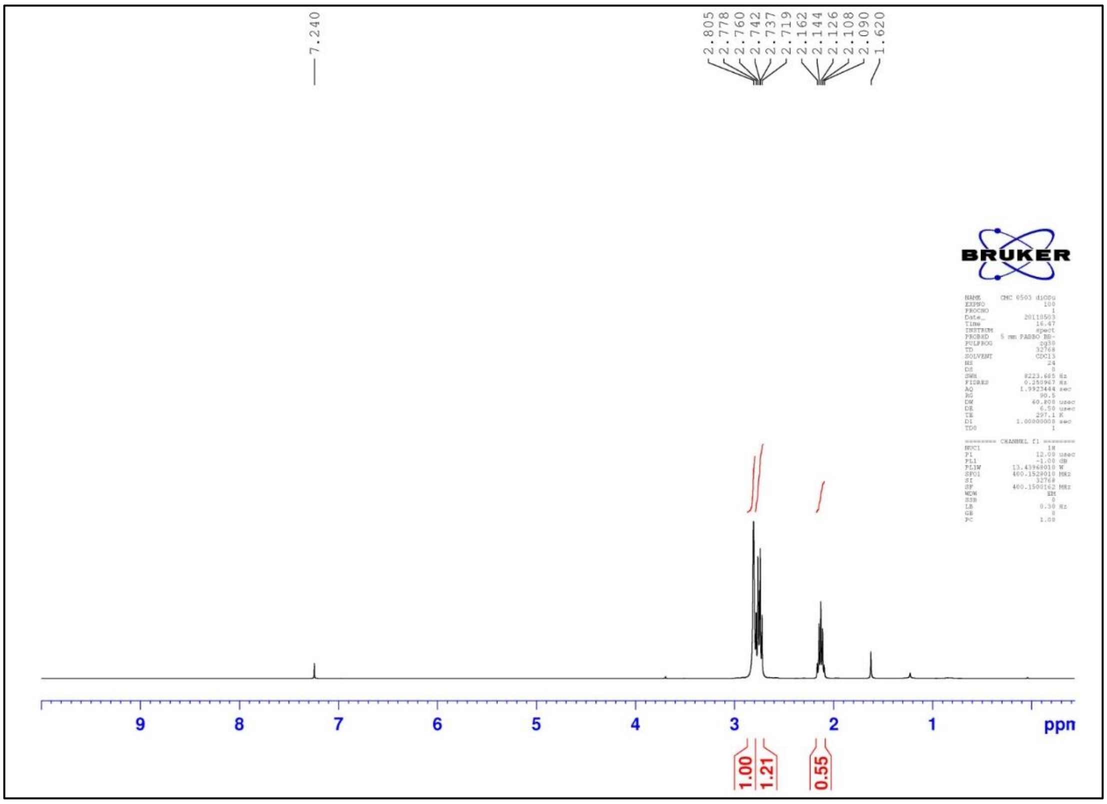

^{1}H NMR spectrum of compound **15** ($CDCl_3$, 400 MHz)

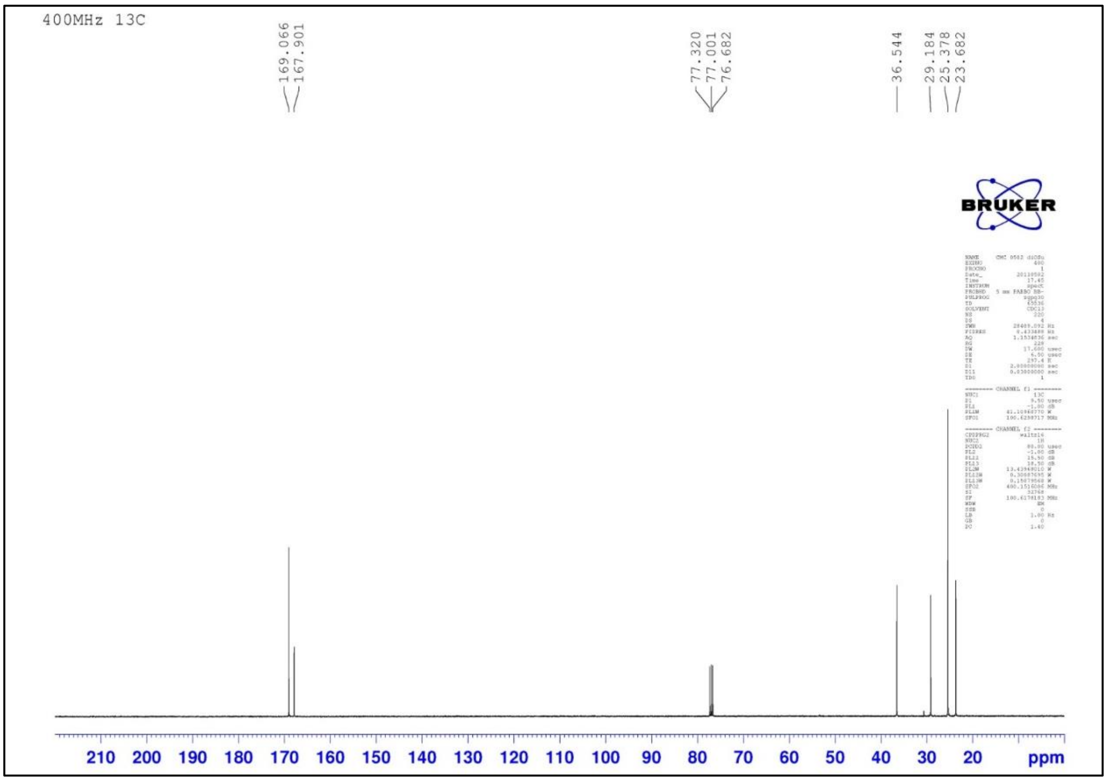

^{13}C NMR spectrum of compound **15** ($CDCl_3$, 100 MHz)

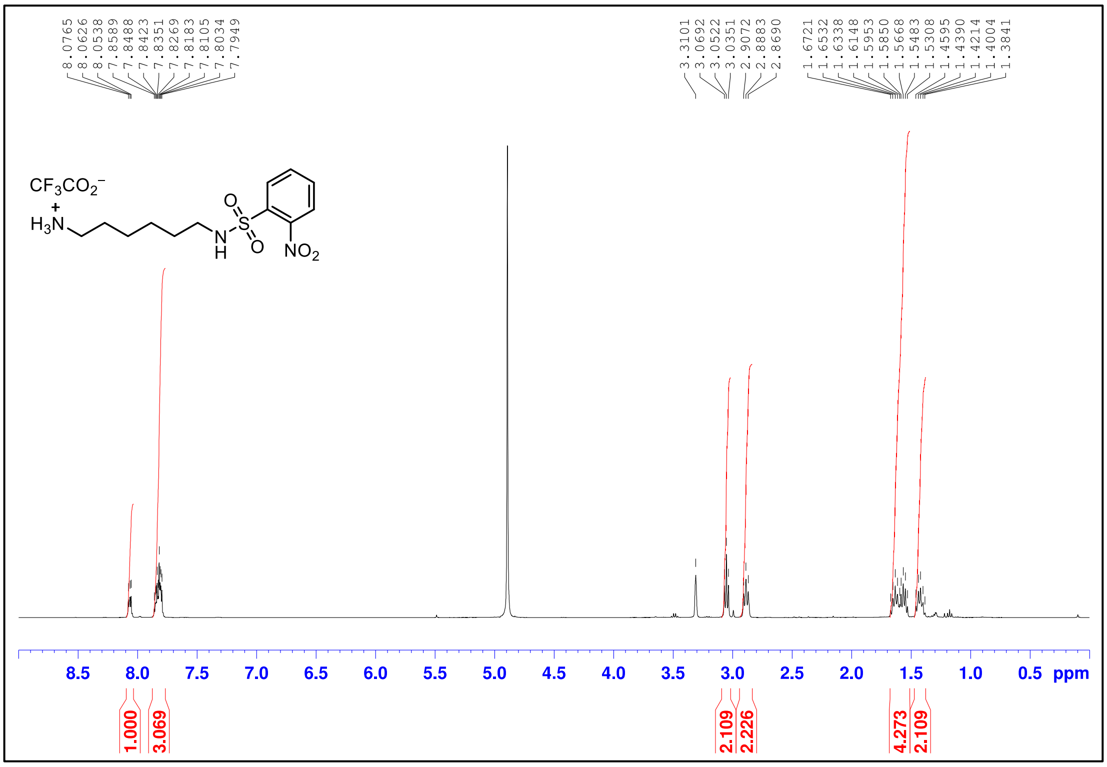

^{1}H NMR spectrum of compound **19** (CD_3OD, 400 MHz)

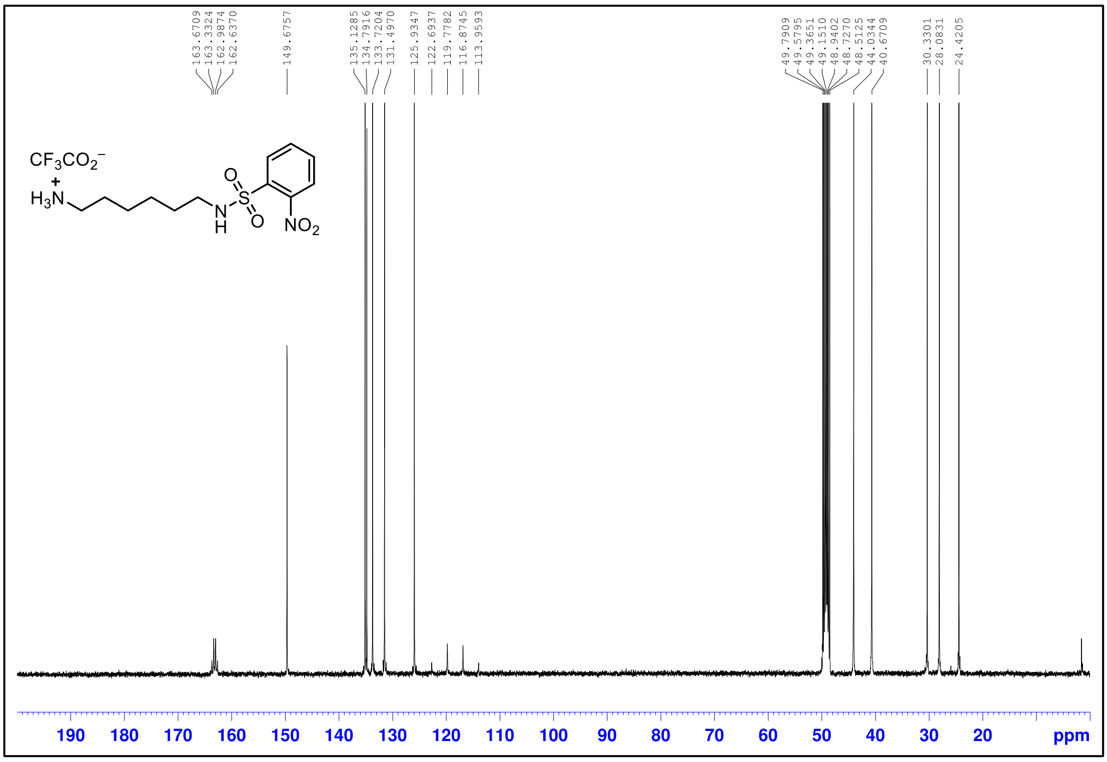

^{13}C NMR spectrum of compound **19** (CD_3OD, 100 MHz)

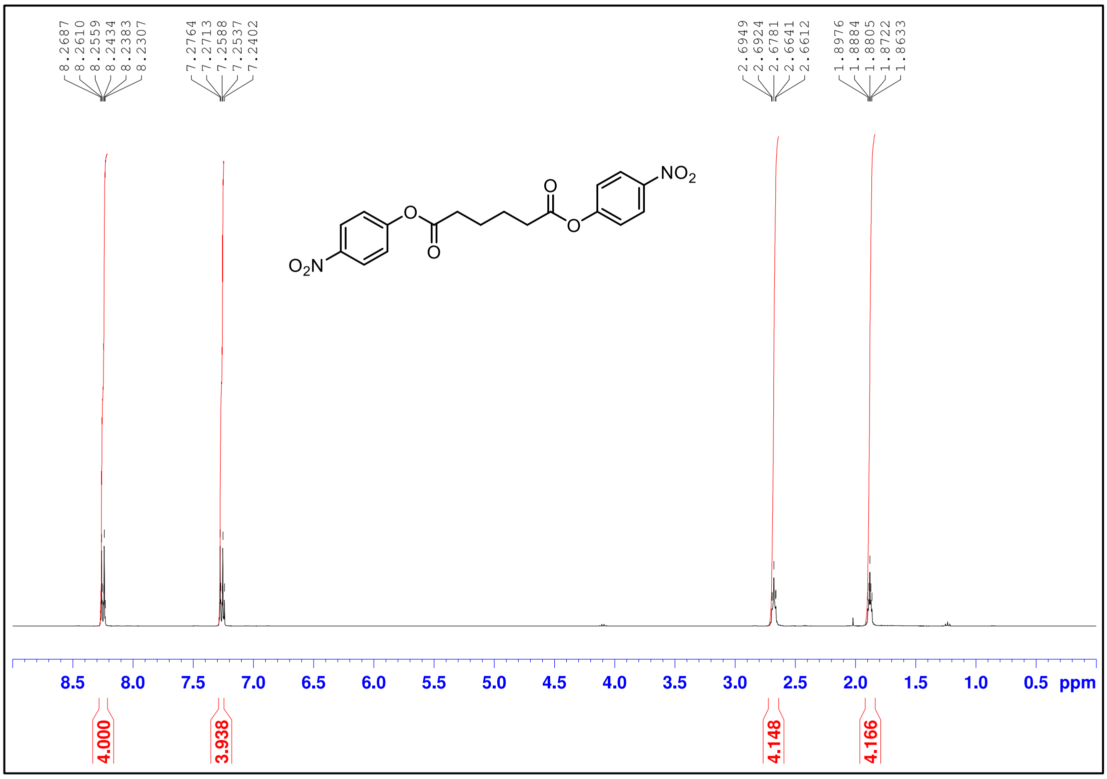

^{1}H NMR spectrum of compound **22** ($CDCl_3$, 400 MHz)

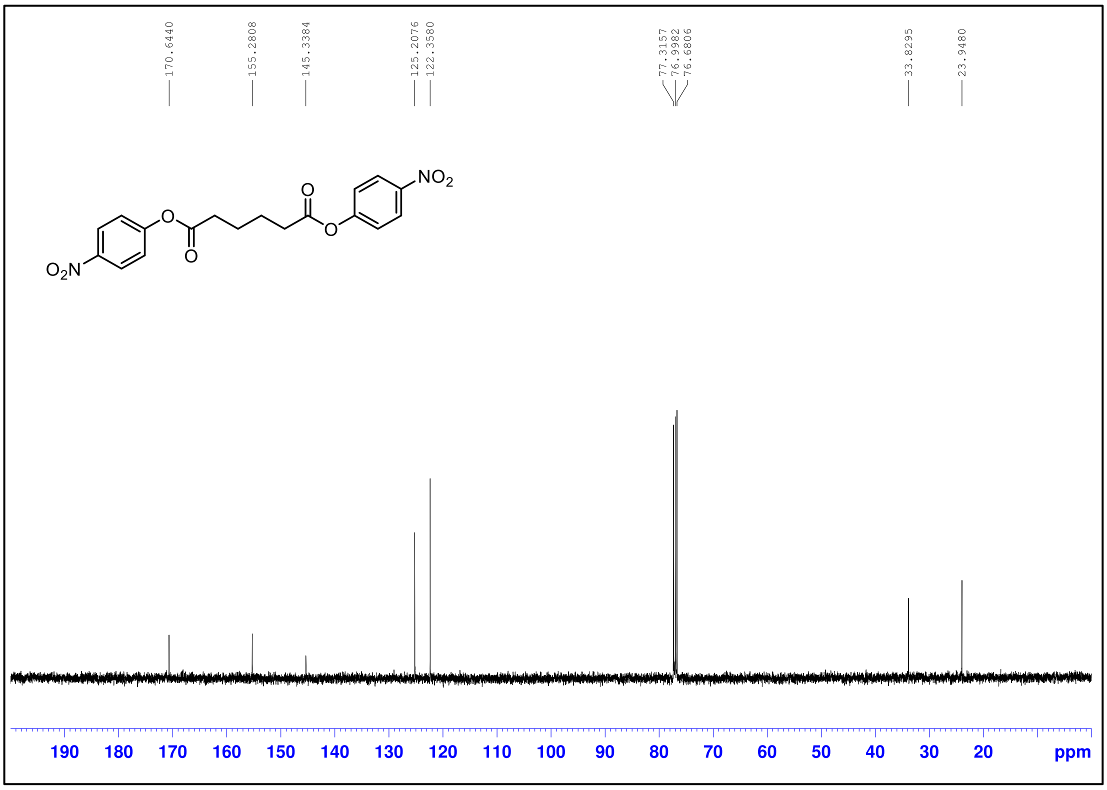

^{13}C NMR spectrum of compound **22** ($CDCl_3$, 100 MHz)

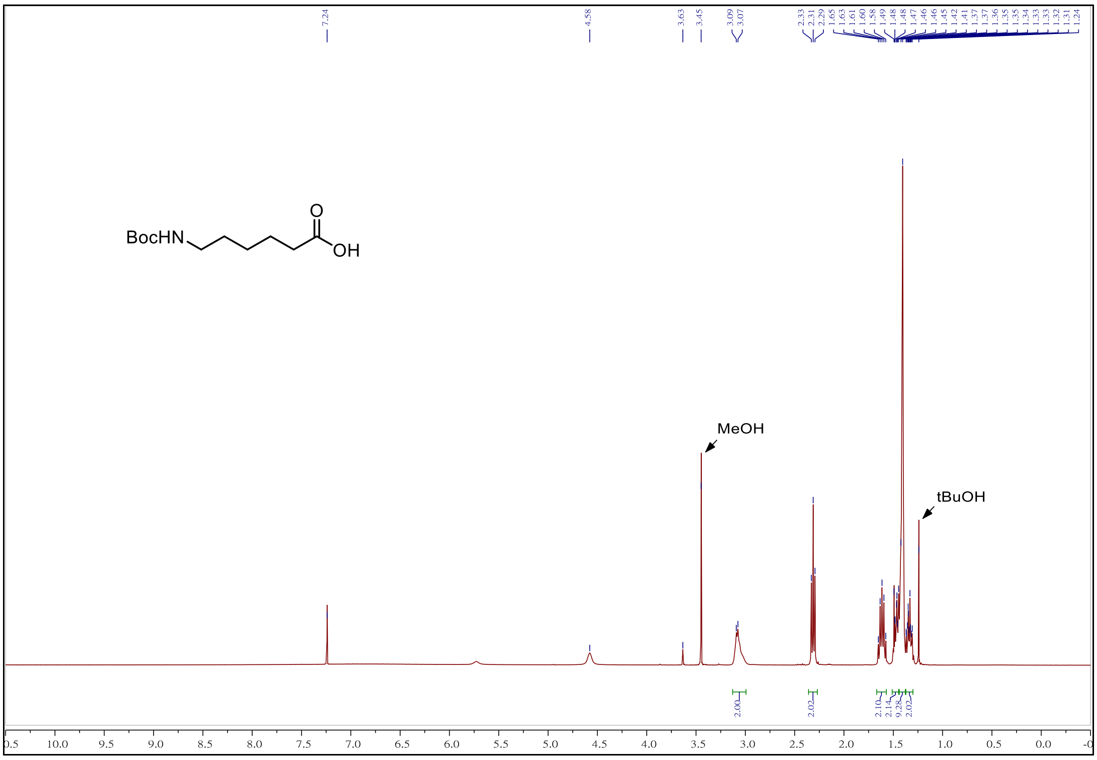

^{1}H NMR spectrum of compound **25** ($CDCl_3$, 400 MHz)

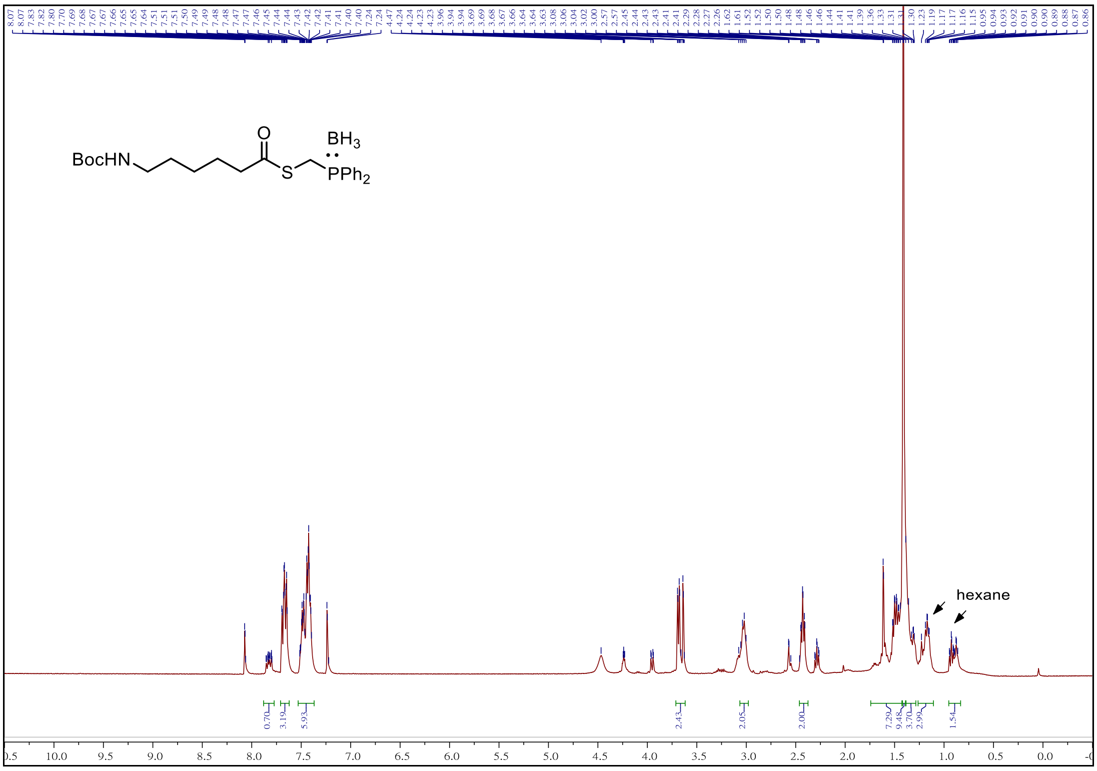

^{1}H NMR spectrum of compound **27** ($CDCl_3$, 400 MHz)

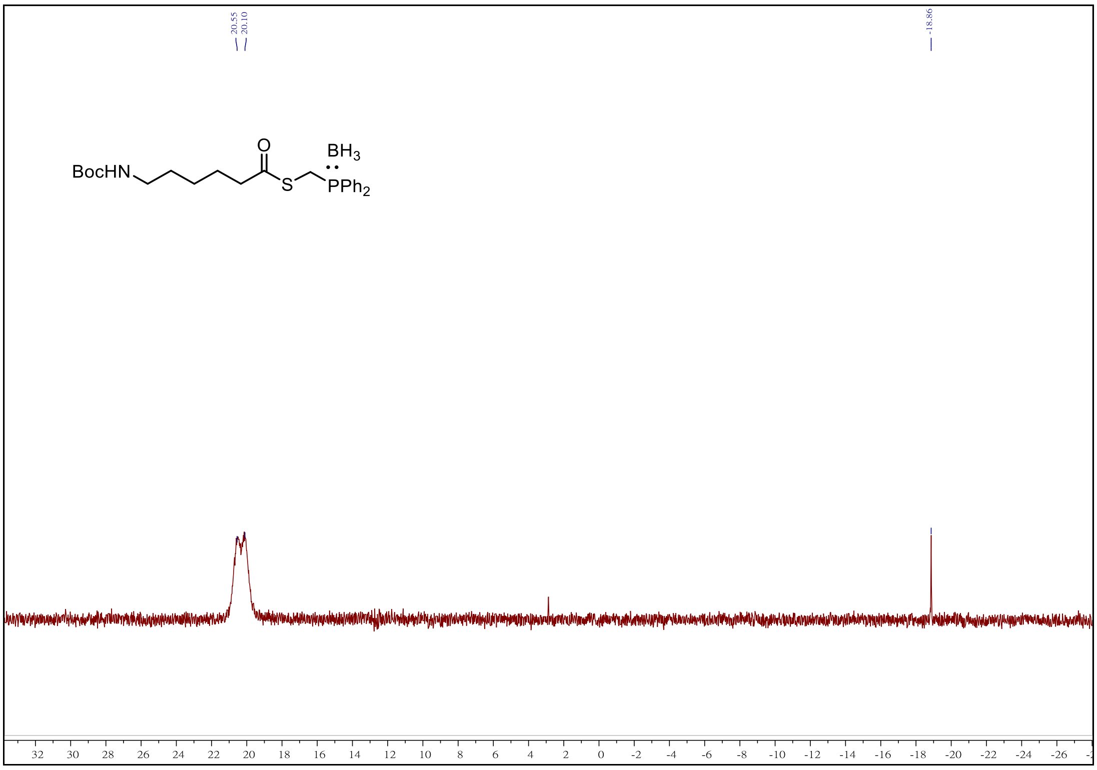

^{31}P NMR spectrum of compound **27** ($CDCl_3$, 162 MHz)

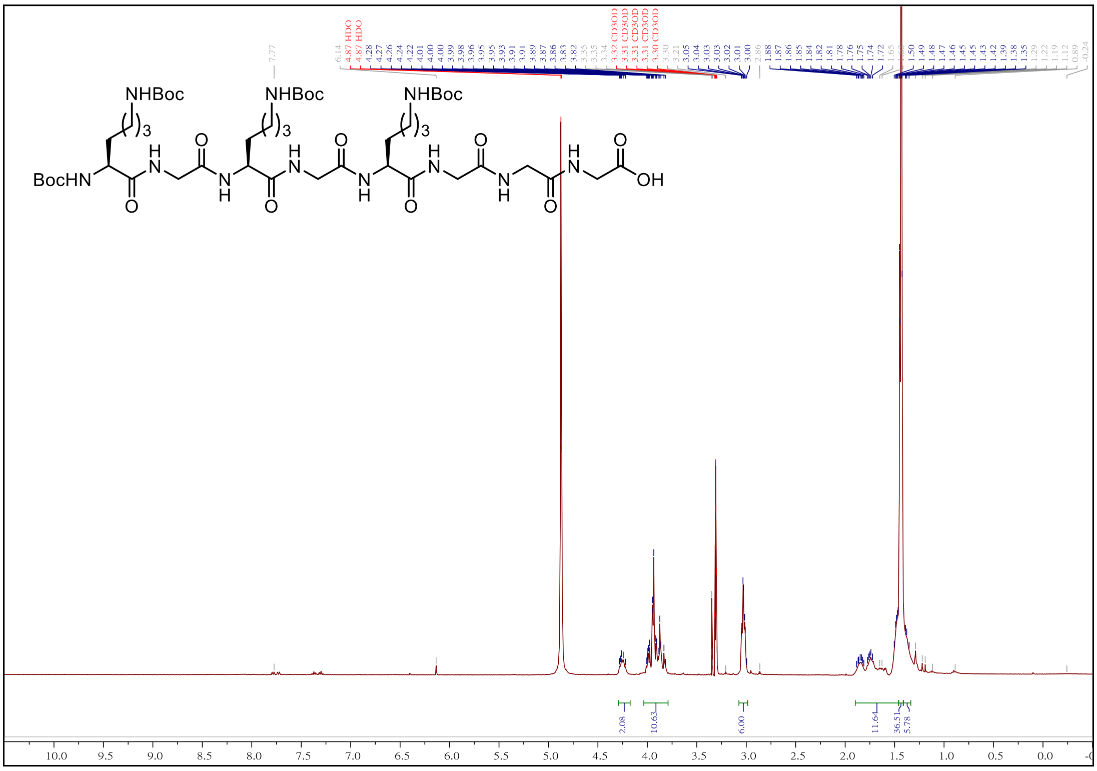

^{1}H NMR spectrum of Boc-protected octapeptide **31** (CD_3OD, 400 MHz)

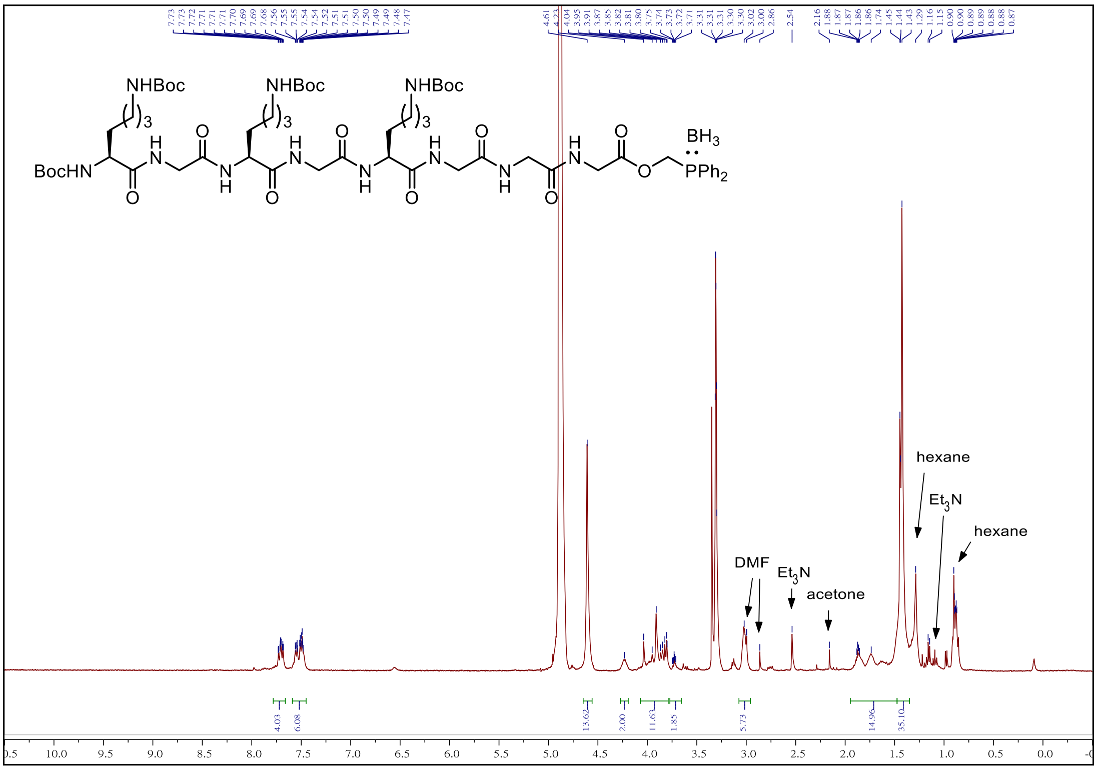

^{1}H NMR spectrum of Boc and borane protected octapeptide **32** (CD_3OD, 400 MHz)
